# QTL Mapping of Traits Associated with Dual Resistance to the African Stem Borer (*Busseola fusca*) and Spotted Stem Borer (*Chilo partellus*) in Sorghum (*Sorghum bicolor*)

**DOI:** 10.1155/2021/7016712

**Published:** 2021-01-15

**Authors:** Phyllis W. Muturi, Mary Mgonja, Patrick Rubaihayo, James K. Mwololo

**Affiliations:** ^1^Department of Agricultural Resource Management, University of Embu, P.O. Box 60100, Embu, Kenya; ^2^Alliance for a Green Revolution in Africa, P.O. Box 34441, Dar es Salaam, Tanzania; ^3^Department of Agricultural Production, Makerere University, P.O. Box 7062, Kampala, Uganda; ^4^East and South African Research Program, International Crops Research Institute of the Semi-Arid Tropics (ICRISAT), P.O. Box 1096 Lilongwe, Malawi

## Abstract

Sorghum (*Sorghum bicolor* (L.) Moench) is an important food crop in semi-arid tropics. The crop grain yield ranges from 0.5 t/ha to 0.8 t/ha compared to potential yields of 10 t/ha. The African stem borer *Busseola fusca* Fuller (Noctuidae) and the spotted stem borer *Chilo partellus* Swinhoe (Crambidae), are among the most economically important insect pests of sorghum. The two borers can cause 15% - 80% grain yield loss in sorghum. Mapping of QTLs associated with resistance traits to the two stem borers is important towards marker-assisted breeding. The objective of this study was to map QTLs associated with resistance traits to *B. fusca* and *C. partellus* in sorghum. 243 F_9:10_ sorghum RILs derived from ICSV 745 (S) and PB 15520-1 (R) were selected for the study with 4,955 SNP markers. The RILs were evaluated in three sites. Data was collected on leaf feeding, deadheart, exit holes, stem tunnels, leaf toughness, seedling vigour, bloom waxiness, and leaf glossiness. ANOVA for all the traits was done using Genstat statistical software. Insect damage traits and morphological traits were correlated using Pearson's correlation coefficients. Genetic mapping was done using JoinMap 4 software, while QTL analysis was done using PLABQTL software. A likelihood odds ratio (LOD) score of 3.0 was used to declare linkage. Joint analyses across borer species and sites revealed 4 QTLs controlling deadheart formation; 6 controlling leaf feeding damage; 5 controlling exit holes and stem tunneling damages; 2 controlling bloom waxiness, leaf glossiness, and seedling vigour; 4 conditioning trichome density; and 6 conditioning leaf toughness. Joint analyses for *B. fusca* and *C. partellus* further revealed that marker *CS132-2* colocalised for leaf toughness and stem tunneling traits on QTLs 1 and 2, respectively; thus, the two traits can be improved using the same linked marker. This study recommended further studies to identify gene(s) underlying the mapped QTLs.

## 1. Introduction

Sorghum (*Sorghum bicolor* (L.) Moench) is an important food crop in drought prone areas in the tropics. The crop is cultivated by more than 500 million resource-challenged smallholder farmers, mostly women [1]. The crop is an important industrial crop in East Africa and has untapped potential in bioenergy production [2]. The African stem borer, *Busseola fusca* Fuller (Noctuidae) and the spotted stem borer *Chilo partellus* Swinhoe (Crambidae), are among the most economically important insect pests of sorghum and maize in Eastern and South Africa [3]. The two stem borer species cause leaf feeding, deadheart formation, exit holes, and stem tunneling damages in cereals [3]. Stem borers are associated with grain yield loss of 15% - 80% depending on borer species population and variety phenological stage at the time of attack [4]. Management approaches such as cultural practices and use of synthetic chemical pesticides have yielded little success in the management of the stem borers. Host plant resistance can reduce sorghum grain yield losses to stem borers and thus improve food security among rural households [5]. Breeding for host plant resistance is part of integrated pest management that can contribute in an economic way in the management of insect pests in indigenous cereals cultivated by resource-challenged smallholder farmers [5]. Breeding for stem borer resistance has been slow partly due to the inadequate understanding of inheritance of traits conditioning resistance to the pests. Stem borer resistance is a polygenic trait controlled by many genes of small effects [6]. Grain sorghum with dual resistance to *B. fusca* and *C. partellus* has been reported [7]. There is no information about genomic regions associated with dual resistance to *B. fusca* and *C. partellus* in grain sorghum. Traits such as leaf toughness, trichomes, bloom waxiness, leaf glossiness, and seedling vigour have been reported to condition resistance to borer and foliar insect resistance in cereals [5, 8–11].

Identification and mapping of quantitative trait loci (QTLs) associated with sorghum resistance to stem borers could enhance the efficiency and effectiveness in marker-assisted breeding [12]. QTL mapping enhances the biological understanding of inheritance of quantitative traits, and the markers identified can be used to select for a complex trait [13]. QTL mapping using recombinant inbred lines (RILs) increases the power of QTL detection compared to the F_2:3_ population because of complete homozygosity at QTLs and marker loci [14]. Marker-assisted selection for stem borer resistance can enhance breeding for *B. fusca* and *C. partellus* since traditional breeding has been unsuccessful. Genetic linkage maps are essential for localization of genes conferring resistance/tolerance to stem borer damage in sorghum [15]. Single nucleotide polymorphism (SNP) markers have become widely accepted as a tool for understanding complex genetic traits and evolution [16]. SNPs have been found to be the most efficient genetic marker for gene identification since they are codominant, highly polymorphic, and have good reproducibility [16]. SNPs represent the finest resolution of a DNA sequence [17]. The genotyping-by-sequencing approach has been employed in whole-genome sequencing to discover SNPs for mapping studies in crop plants [18, 19]. The objective of this study was to identify and characterize QTLs associated with resistance traits to *B. fusca* and *C. partellus* in sorghum.

## 2. Materials and Methods

### 2.1. Development of Mapping Population

243 F_9:10_ recombinant inbred lines (RILs) derived from a cross between the stem borer-susceptible cultivar ICSV 745 and stem borer-resistant PB 15520-1 were selected for the mapping study. The progenies and their parents had been developed following a single seed descent approach in ICRISAT, Patancheru, India [20]. The two parents were crossed, and the resulting F_1_ seeds were advanced to F_2_ by selfing of single F_1_ plants. The F_2_ seeds were selfed and the resulting F_3_ population grown in progeny rows [20]. Single sorghum plants in each of the progeny row were selfed and the process continued up to the F_9_ generation [20]. Seeds from the F_9_ sorghum plants of each row were bulked to produce the 243 F_9:10_ RILs used in this study.

### 2.2. Experimental Design, Site, and Source of Stem Borer Larvae

The 243 RILs along with their parents were imported from the International Crops Research Institute for the Semi-Arid Tropics (ICRISAT), Patancheru, India to Kenya. The sorghum plants were phenotyped at Embu and Kabete for resistance against *B. fusca* for one season in each environment and at Kiboko for resistance against *C. partellus* for two seasons thus totaling to four environments. Each experiment in each site was laid out in a 25 × 10 alpha-lattice design consisting of twenty-five plots in ten blocks, replicated twice. Each plot consisted of 2 m rows with plants spaced at 0.75 m × 0.25 m inter- and intra-rows, respectively. First-instar neonates of the two borer species were obtained from the International Centre of Insect Physiology and Ecology (ICIPE), Nairobi, Kenya. At 30 days after sowing, five plants in each row were tagged and artificially infested with five larvae of the respective stem borer species using a camel hair brush as described by Singh et al. [21] in all the three sites.

### 2.3. Phenotypic Data Collection and Data Analysis

Data on stem borer leaf feeding damage, deadheart incidence, number of exit holes, and stem tunnel length were scored as described by Muturi et al. [7]. Morphological traits measured included leaf toughness, seedling vigour, bloom waxiness, leaf glossiness, and total grain yield as described by Kumar et al. [22]. Data were subjected to analysis of variance using the residual maximum likelihood model (ReML) in Genstat Version 14 statistical package. The predicted means for each genotype were estimated with genotypes as fixed and reps as random effects in individual and across environment analyses. Phenotypic and genetic correlation coefficients were calculated from adjusted entry means across environments for each parameter. Estimates of variance components, including genotypic variance (*σ*^2^*g*), genotype × environment interaction (*σ*^2^*g* × *e*), and residual (*σ*^2^) were calculated by equating the mean squares to their expected values as described by Shimelis and Shiringani [23]. Heritability (*H*) was estimated using Meta-R software version 6 using *H* = (*∂*_*g*_^2^)/(*∂*_*g*_^2^ + *∂*_*g*×*e*/(*r*)_^2^ + *∂*_*e*/(*r* × *env*)_^2^), where *∂*_*g*_^2^ = genotypic variance, *∂*_*g*×*e*_^2^ = genotype by environment variance, *∂*_*e*_^2^ = error variance, *r* = number of replications, and env = number of environments as described by Hallauer and Miranda, [24]. Direct and indirect effect analyses were conducted using path analysis to study interrelationship among resistance parameters and their relationship to grain yield reduction [25]. Genotypic and phenotypic correlation coefficients were calculated following the method described by Holland [26].

### 2.4. DNA Extraction and Quantification

Leaf tissue from the parental lines and the 243 RILs were harvested from 10-day-old sorghum seedlings. DNA was extracted according to [27]. The quality of DNA in each sample was checked using 0.8% agarose gel stained with ethidium bromide. Each well of the agarose gel was loaded with 5 *μ*l of the sample, and the gel was allowed to run at 100 V for 5 minutes. After electrophoresis, DNA banding patterns on the gel were visualized under UV light. A smear of DNA indicated poor quality, and the DNA was re-extracted, whereas a clear band indicated good quality DNA. The quantity of DNA in each sample was assessed using a fluorescence spectrophotometer (Switzerland) by staining the DNA with PicoGreen™ (1/200 dilution). The DNA concentration of each sample was calculated and then normalized to 2.5 ng/*μ*l for the PCR.

### 2.5. Scoring of Sequenced Products and Construction of Linkage Map

**Figure 1:**
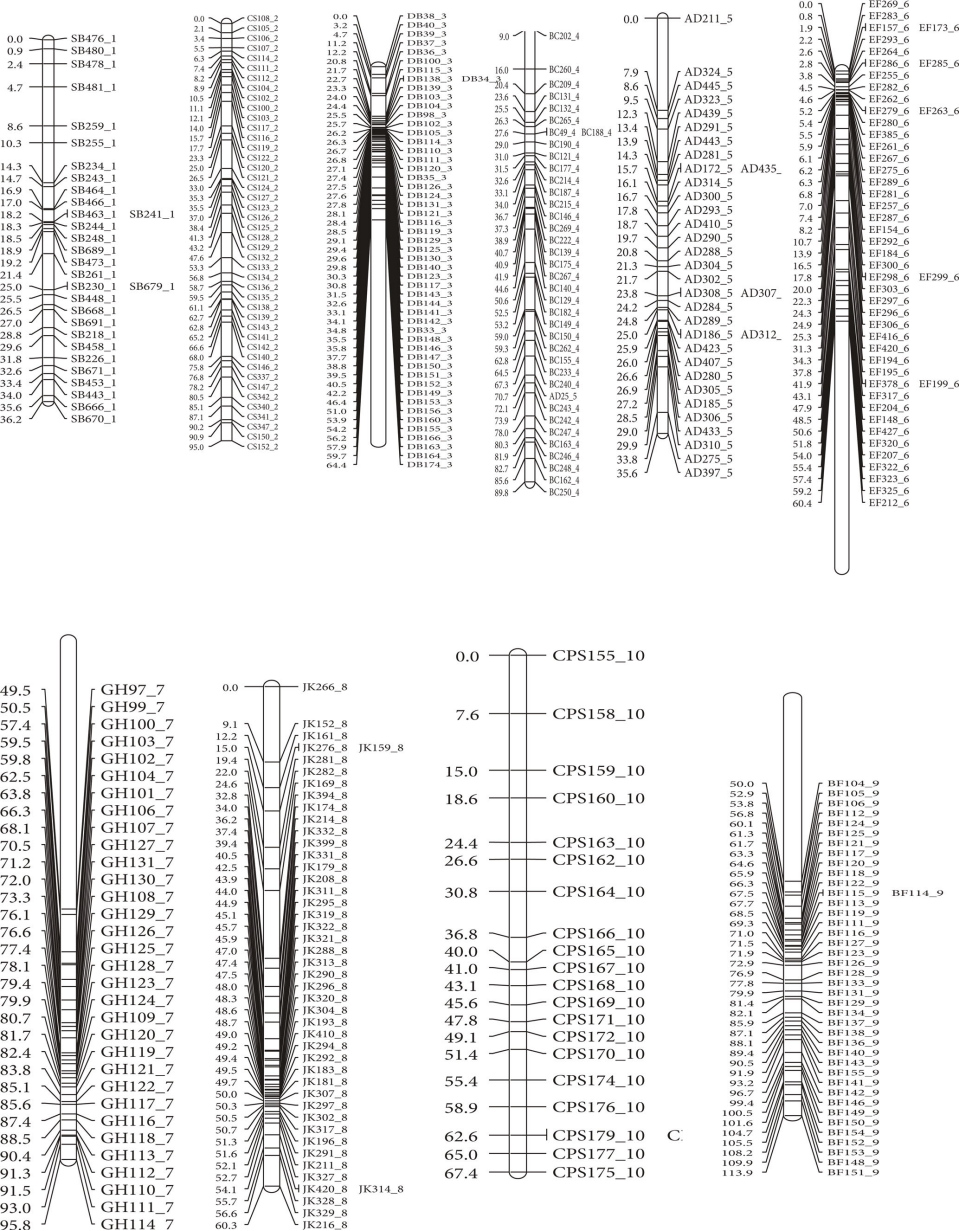
Genetic map of ICSV 745 × PB 15520 recombinant inbred lines of sorghum generated using 4,955 polymorphic SNP markers.

The SNP markers screened on the RILs were scored as follows: A = homozygote carrying allele from female parent (ICSV 745); B = homozygote carrying allele from male parent (PB 15520-1); and - = missing data for an individual at a locus. The genotypic data was used to construct a genetic linkage map, which spanned 4,692.4 cM, with a total of 4,955 SNP markers distributed into the 10 sorghum linkage groups (Figure 1).

Segregation of each marker was tested with a chi-square goodness of fit test to the expected Mendelian segregation ratio (1 : 1) of parental configuration. The names of the markers were coded for ease of analysis (Supplementary Material Table 14). The markers that did not conform to the expected segregation ratio were excluded from the analysis. Three-point linkage analysis was performed for each linkage group, and the most saturated linkage was adopted as described by Ooijen [28]. After the addition of each loci, a ripple was used to verify local locus orders [28].

### 2.6. QTL Analysis

QTL analyses were performed on morphological data and on leaf feeding, deadheart, exit holes, and stem tunneling damage under infestation with *B. fusca* at the Kabete and Embu sites and the same traits under infestation with *C. partellus* at the Kiboko site. Joint analysis was also done using averaged means across environments and insect species. Composite interval mapping (CIM) was performed on the data using PLABQTL software, version 1.2 [29]. Whole-genome scan with CIM was conducted using an automatic cofactor selection model to determine additive effects at individual QTL, and the *F* value of ten options were selected. Cofactors were chosen by step-wise regression and Akaike's information criterion (AIC) of 3.0 with the “cov” statement in PLABQTL. Dependence of QTL estimation on sampling effects was estimated by a five-fold cross validation by dividing the genotypes into five subsets. The LOD threshold for declaring a putative QTL was set to 3.0 after performing 1,000 permutation tests (type I error level *α* = 10%). All QTLs identified in this study explained ≥10% of the total phenotypic variation and were thus classified as major QTLs [30].

## 3. Results

### 3.1. Phenotypic Analysis

#### 3.1.1. Results for Combined Analyses of Variance across Environments

The combined results for the two borer species suggested that plant damage (deadheart formation, leaf feeding damage, exit holes, and stem tunnels) and agro-morphological traits (seedling vigour and bloom waxiness) evaluated in this study were influenced significantly (*P* < 0.01) by borer species tested, genotype, and the environment where the experiment was conducted (Table 1).

Results of mean squares for leaf feeding, deadheart, exit holes and morphological traits at Kiboko are presented in Table 2. There were significant differences observed for all the traits measured except for leaf feeding damage. Exit holes and stem tunneling damages were the only plant damage traits that were significantly (*P* < 0.01) influenced by genotype by season interaction (Table 2). Results of mean squares for leaf feeding, deadheart, exit holes, and morphological traits at Embu are presented in Table 3. There were significant (*P* < 0.01) genotypic differences on all the traits evaluated at Embu site for *B. fusca* (Table 3).

Results of mean squares for leaf feeding, deadheart, exit holes, and morphological traits at Kabete are presented in Table 4. Genotype significantly (*P* < 0.01) influenced all the traits evaluated except leaf feeding damage (Table 4). Heritability estimates and their standard errors for the sorghum damage and morphological traits across the three environments are presented in Table 5. It was noted that heritability estimates for *C. partellus* at Kiboko were generally low for most of the traits compared to the two other sites where *B. fusca* was tested.

The contribution of *B. fusca* damage parameters to total grain yield through leaf feeding, deadheart, stem tunnels, and number of exit hole damages at Embu are presented in Table 6. Damage effects were partitioned into direct and indirect associations through path coefficient analysis, and grain yield was used as the resultant variable (Table 6). Deadheart, exit holes, leaf feeding, and stem tunneling damages had a negative correlation with grain yield. Deadheart had a significant direct positive effect on grain yield supported by an indirect positive effect through stem tunneling and indirect negative effects through the number of exit holes and leaf feeding damages. Exit holes had a direct positive effect on grain yield supported by indirect positive effects through deadheart and leaf feeding damages and an indirect negative effect through stem tunneling. Leaf feeding had a direct positive effect on grain yield supported by indirect positive effects through deadheart and the number of exit holes and an indirect negative effect through stem tunneling. Stem tunneling had a direct positive effect on grain yield supported by indirect positive effects through deadheart, number of exit holes, and leaf feeding damages. The contribution of *B. fusca* damage parameters to total grain yield through leaf feeding, deadheart formation, stem tunnels, and number of exit hole damages at Kabete is presented in Table 7.

Deadheart, exit holes, leaf feeding, and stem tunneling damages had a negative correlation with grain yield. Deadheart had a direct positive effect on grain yield supported by indirect positive effects on exit holes, leaf feeding, and stem tunneling damages. The number of exit holes had a direct positive effect on grain yield supported by indirect positive effects through deadheart, leaf feeding, and stem tunneling damages. Leaf feeding damage had a direct positive effect on grain yield supported by an indirect positive effect through deadheart damage and indirect negative effects through exit hole and stem tunneling damages. Stem tunneling had direct positive effects on grain yield supported by indirect negative effects through deadheart, the number of exit holes, and leaf feeding damages. The contribution of *C. partellus* damage traits to total grain yield through leaf feeding, deadheart, stem tunneling, and the number of exit hole damages at Kiboko are presented in Table 8.

Deadheart, exit holes, leaf feeding, and stem tunneling damages had a negative correlation with grain yield (Table 8). Deadheart had a direct positive effect on grain yield supported by an indirect positive effect through stem tunneling and an indirect negative effect through exit holes and leaf feeding damages. Exit holes had direct positive effects on grain yield supported by an indirect positive effect through leaf feeding and stem tunneling damages and an indirect positive effect through deadheart damage. Leaf feeding had a direct positive effect on grain yield supported by indirect negative effects through deadheart, exit holes, and stem tunneling damages. Stem tunneling had a direct positive effect on grain yield supported by indirect positive effects through deadheart, exit holes, and leaf feeding damages.

#### 3.1.2. Correlations of Means among Stem Borer Damage and Agro-morphological Traits

The results for correlations of mean for *B. fusca* damage and agro-morphological traits in Embu, Kenya are presented in Table 9. A significant positive correlation was observed between panicle length and plant height (*r* = 0.45, *P* ≤ 0.001), implying that taller plants had a long panicle length. Stem tunneling had a significant positive association with plant height (*r* = 0.18, *P* = 0.0057) and with exit holes (*r* = 0.71, *P* ≤ 0.001). Seedling vigour had a positive and significant relationship with plant height (*r* = 0.21, *P* = 0.0011). Exit holes had a significant positive relationship with deadheart damage (*r* = 0.15, *P* = 0.02). Leaf damage had a significant positive relationship with deadheart damage (*r* = 0.25, *P* ≤ 0.001). A significant positive correlation was observed between trichome density and leaf glossiness (*r* = 0.18, *P* = 0.0039), with seedling vigour (*r* = 0.14, *P* = 0.033), and with bloom waxiness (*r* = 0.17, *P* = 0.0075). A significant positive correlation was recorded between seedling vigour and panicle length (*r* = 0.13, *P* = 0.03). Bloom waxiness had a significant positive correlation with seedling vigour (*r* = 0.60, *P* ≤ 0.001). A significant negative relationship was recorded between seedling vigour and deadheart formation (*r* = −0.40, *P* ≤ 0.001) and with exit holes (*r* = −20, *P* = 0.002). Bloom waxiness had a significant negative correlation with deadheart damage (*r* = −0.39, *P* ≤ 0.001), with exit holes (*r* = −0.33, *P* ≤ 0.001), and with stem tunneling (*r* = −20, *P* = 0.0016), respectively.

The results for correlations of mean for *B. fusca* damage and agro-morphological traits at Kabete, Kenya are presented in Table 10.

Leaf feeding had a significant positive correlation with deadheart damage (*r* = 0.31, *P* ≤ 0.001). Stem tunneling had a significant positive relationship with exit holes (*r* = 0.70, *P* ≤ 0.001). Bloom waxiness had a significant positive correlation with leaf glossiness (*r* = 0.20, *P* = 0.002) and with seedling vigour (*r* = 0.15, *P* = 0.02). Yield had a significant positive correlation with plant height (*r* = 0.15, *P* = 0.021) and with panicle length (*r* = 0.30, *P* ≤ 0.001). Seedling vigour had a significant negative correlation with deadheart damage (*r* = −0.38, *P* ≤ 0.001), with exit holes (*r* = −0.15, *P* = 0.02), and with stem tunneling (*r* = −0.17, *P* = 0.008). Days to 50% flowering had a significant negative correlation with the number of exit holes (*r* = −0.13, *P* = 0.04).

The results for correlations of mean for *C. partellus* damage and agromorphological traits at Kiboko, Kenya are presented in Table 11. Stem tunneling had a significant positive correlation with exit holes (*r* = 0.68, *P* ≤ 0.001). Days to 50% flowering had a positive correlation with leaf damage (*r* = 0.17, *P* = 0.008) and with plant height (*r* = 0.13, *P* = 0.04). Panicle length had a significant positive relationship with plant height (*r* = 0.27, *P* ≤ 0.001), with bloom waxiness (*r* = 0.13, *P* = 0.04), and with total grain yield (*r* = 0.34, *P* ≤ 0.001). Seedling vigour had a significant positive correlation with bloom waxiness (*r* = 0.13, *P* = 0.04). Days to 50% flowering had a significant negative correlation with stem tunneling (*r* = −0.13, *P* = 0.04) and with seedling vigour (*r* = −0.33, *P* ≤ 0.001). Stem tunneling had a significant negative relationship with bloom waxiness (*r* = −0.26, *P* ≤ 0.001). Leaf feeding damage had a significant negative correlation with seedling vigour (*r* = −0.13, *P* = 0.04) and with total grain yield (*r* = −0.16, *P* = 0.01).

#### 3.1.3. QTL Mapping

QTL analysis with composite interval mapping was performed for the damage traits under artificial infestation with *B. fusca* at the Embu and Kabete sites, and the same traits were evaluated for *C. partellus* at Kiboko under artificial infestation as described in Materials and Methods. The results for damage and morphological traits are shown in Table 12. Three QTLs for deadheart damage due to *B. fusca* were detected on chromosomes 1, 3, and 5 (flanked by markers *SB261_1*, *DB103_3*, and *AD323_5*) at Embu (Table 12) that explained 3.9% of the phenotypic variation, with individual QTLs accounting for 7.5% - 11.2%. A simultaneous fit with all the three QTLs based on cross validation explained 28.3% of the adjusted genetic variance. The additive gene action ranged from -7.79 to 35.13. Two of the QTLs on chromosomes 1 and 5 (flanked by markers *SB261_1* and *AD323_5*) were from the susceptible parent, while the remaining QTLs on chromosome 3 (marker *DB103_3*) came from the resistant parent (PB 15520-1). The resistant parent contributed to less deadheart damage, which is a putative trait associated with resistance to stem borers in sorghum.

Five QTLs for leaf feeding damage due to *B. fusca* at Embu site were detected on chromosomes 2 and 10 (flanked by markers *CS115_2*, *CS190_2*, *CS389_2*, *CS414_2*, and *CPS162_10*, respectively) (Table 12). These explained 10.5% of the phenotypic variation, with individual QTLs accounting for 7.1% - 9.1%. A simultaneous fit with all the five QTLs based on cross validation explained 40.6% of the adjusted genetic variance. The additive gene action ranged from -6.48 to 8.13. Three of the QTLs on chromosomes 2 and 10 (flanked by markers *CS190_2*, *CS389_2*, and *CPS162_10*) came from the susceptible parent. The rest of the QTLs on chromosome 2 (flanked by markers *CS115_2* and *CS414_2*) came from the resistant parent (PB 15520-1), which contributed to less leaf feeding damage, which is a trait associated with resistance to stem borers in sorghum.

One QTL for exit hole damage due to *B. fusca* at Embu site was detected on chromosome 7 flanked by marker *GH89_7* (Table 12). This explained 6.1% of the phenotypic variation, with individual QTL accounting for 9%. A simultaneous fit with the single QTL based on cross validation explained 9% of the adjusted genetic variance. The additive gene action was -1.42. This QTL came from the resistant parent (PB 15520-1) which contributed to less exit hole damage, a trait associated with resistance to stem borers in sorghum.

Two QTLs for stem tunneling damage by *B. fusca* at Embu site were detected on chromosomes 7 and 9, respectively (flanked by markers *GH87_7* and *BF123_9*) (Table 12). These explained 5.9% of the phenotypic variation, with individual QTLs accounting for 8.1% - 8.2%. A simultaneous fit with the two QTLs based on cross validation explained 16.3% of the adjusted genetic variance. The additive gene action ranged from -2.39 to -7.33. The two QTLs came from the resistant parent (PB 15520-1), an indication of the transfer of genes associated with resistance to stem tunneling by borers in sorghum.

Ten QTLs for deadheart damage due to *B. fusca* were detected on chromosomes 1, 2, 3, 4, 8, and 5, respectively, (flanked by markers *SB691_1*, *CS402_2*, *CS259_2*, *CS350_2*, *DB172_3*, *DB169_3*, *BC149_4*, *JK399_8*, *BF97_9*, and *BF106_9*) (Table 12). These explained 2.8% of the phenotypic variation, with individual QTLs accounting for 7.6% - 17.5%. A simultaneous fit with all the ten QTLs based on cross validation explained 80.6% of the adjusted genetic variance. The additive gene action ranged from -20.68 to 12.95. Four of the QTLs on chromosomes 1, 2, 4, and 9 (flanked by markers on markers *SB691_1*, *CS259_2*, *BC149_4*, and *BF97_9*) were from the susceptible parent, while the QTLs on chromosomes 2, 3, 8, and 9 (flanked by markers *CS402_2*, *CS350_2*, *DB172_3*, *DB169_3*, *JK399_8*, and *BF106_9*) came from the resistant parent (PB 15520) which contributed to less deadheart damage.

Seven QTLs for leaf feeding damage due to *B. fusca* at Kabete site were detected on markers on chromosomes 2, 3, 6, 8, and 10 (flanked by markers *CS403_2*, *CS111_2*, *CS397_2*, *DB164_3*, *EF334_6*, *m08/014.9*, and *CPS158_10*) (Table 12). These explained 1.3% of the phenotypic variation, with individual QTLs accounting for 7% - 13%. A simultaneous fit with all the seven QTLs based on cross validation explained 66.8% of the adjusted genetic variance. The additive gene action ranged from -14.42 to 6.56. Three of the QTLs on chromosomes 2 and 10 (flanked by markers *CS111_2*, *CS397_2*, and *CPS158_10*, respectively) came from the susceptible parent (ICSV 745). QTLs on markers on chromosomes 2, 3, 6, and 8 (flanked by markers *CS403_2*, *DB164_3*, *EF334_6*, and *m08/014.9*, respectively) came from the resistant parent (PB 15520-1) which contributed to less leaf feeding damage.

Eight QTLs for exit holes due to *B. fusca* at Kabete site were detected on chromosomes 2, 5, 6, 8, and 9 (flanked by markers *CS342_2*, *CS190_2*, *AD25_5*, *AD185_5*, *EF255_6*, *JK208_8*, *BF138_9*, and *BF137_9*) (Table 12). These explained 6.1% of the phenotypic variation, with individual QTLs accounting for 7.5%–16.5%. A simultaneous fit with all the eight QTLs based on cross validation explained 84.7% of the adjusted genetic variance. The additive gene action ranged from -7.28 to 1.17. Two of the QTLs on chromosomes 2 and 5 (flanked by markers *CS342_2* and *AD185_5*, respectively) came from the susceptible parent (ICSV 745). The rest of the six QTLs on chromosomes 2, 5, 6, 8, and 9 (flanked by markers *CS190_2*, *AD25_5*, *EF255_6*, *JK208_8*, *BF138_9*, and *BF137_9*) came from the resistant parent (PB 15520-1) which contributed to less exit hole damage.

Nine QTLs for stem tunneling damage due to *B. fusca* at Kabete site were on chromosomes 1, 2, 3, 4, and 7 (flanked by markers *SB466_1*, *CS132_2*, *CS133_2*, *CS150_2*, *DB140_3*, *DB143_3*, *BC140_4*, *GH87_7*, and *GH108_7*) (Table 12). These QTLs explained 0.16% of the phenotypic variation, with individual QTLs accounting for 7.1% – 13.9%. A simultaneous fit with all the nine QTLs based on cross validation explained 74.29% of the adjusted genetic variance. The additive gene action ranged from -4.53 to 3.98. Four of the QTLs on chromosomes 1, 3, and 4 (flanked by markers *SB466_1*, *DB140_3*, *DB143_3*, and *BC140_4*) came from the susceptible parent (ICSV 745). The other five QTLs on chromosomes 2 and 7 (flanked by markers *CS132_2*, *CS133_2*, *CS150_2*, *GH87_7*, and *GH108_7*) came from the resistant parent (PB 15520-1) which contributed to less stem tunneling damage.

Four QTLs for deadheart damage due to *C. partellus* at Kiboko site were detected on chromosomes 2, 6, and 9 (flanked by markers *CS369_2*, *CS389_2*, *EF322_6*, and *BF152_9*) (Table 12). These QTLs explained 2.7% of the phenotypic variation with individual QTLs accounting for 8.3% – 12.6%. A simultaneous fit with all the four QTLs based on cross validation explained 41.2% of the adjusted genetic variance. The additive gene action ranged from -8.46 to 3.32. One of the QTLs on chromosome 2 (flanked by marker *CS369_2*) came from the susceptible parent (ICSV 745), while the other three QTLs came from the resistant parent (PB 15520-1) which contributed to less deadheart damage.

Three QTLs for leaf feeding damage due to *C. partellus* at Kiboko site were detected on chromosomes 2 and 6 (flanked by markers *CS133_2*, *CS397_2*, and *EF184_6*) (Table 12). The markers explained 4.1% of the phenotypic variation with individual QTLs accounting for 7.2% – 8.7%. A simultaneous fit with all the three QTLs based on cross validation explained 24.5% of the adjusted genetic variance. The additive gene action ranged from -3.07 to 2.76. All the three QTLs came from the resistant parent (PB 15520-1) which contributed to reduce leaf feeding damage.

Seven QTLs for exit hole damage due to *C. partellus* at Kiboko site were detected on chromosomes 2, 3, 4, 5, 6, and 7 (flanked by markers *CS414_2*, *DB153_3*, *DB208_3*, *BC222_4*, *m05/015.6*, *EF255_6*, and *GH66_7*) (Table 12). The QTLs explained 1.4% of the phenotypic variation with individual QTLs accounting for 7.2% – 10.3%. A simultaneous fit with all the seven QTLs based on cross validation explained 58.5% of the adjusted genetic variance. The additive gene action ranged from -8.04 to 0.61. Two of the QTLs on chromosomes 2 and 3 (flanked by markers *CS414_2* and *DB153_3*) came from the susceptible parent (ICSV 745). The other five QTLs came from the resistant parent (PB 15520-1), an indication of the transfer of the resistance genes to the progenies.

Three QTLs for stem tunneling damage due to *C. partellus* at Kiboko site were detected on chromosomes 3 and 7 (flanked by markers *DB152_3*, *GH70_7*, and *GH118_7*) (Table 12). These explained 0.29% of the phenotypic variation, with individual QTLs accounting for 7.8% –11.4%. A simultaneous fit with all the three QTLs based on cross validation explained 28.7% of the adjusted genetic variance. The additive gene action ranged from -3.56 to 2.64. One of the QTLs on chromosome 3 flanked by marker *DB152_3* came from the susceptible parent (ICSV 745), while the other two QTLs came from the resistant parent (PB 15520-1).

Colocalization of markers for some of the traits evaluated was detected on site analysis for *B. fusca* (at Embu and Kabete), and *C. partellus* (at Kiboko) (Table 12). For example, it was observed that the region flanked by markers (*DB172_3*, *EF184_6*, and *BF123_9*) on chromosomes 3, 6, and 9 conditioned trichome density, deadheart damage, and stem tunneling damage at Kabete. Marker *DB169_3* on chromosome 3 conditioned leaf glossiness and deadheart damage at Kabete. Marker *CS403_2* on chromosome 2 conditioned seedling vigour and leaf damage at Kabete and Embu, respectively. Marker *AD323_5* on chromosome 5 conditioned resistance to deadheart formation at Embu as well as seedling vigour at Embu. Marker *CS259_2* on chromosome 2 conditioned resistance to deadheart formation at Kabete as well as bloom waxiness in Kiboko. Marker *GH108_7* on chromosome 7 conditioned resistance to seedling vigour at Kiboko as well as stem tunneling in Kabete. Marker *AD323_5* on chromosome 5 conditioned resistance to seedling vigour and deadheart damage at Embu. Marker *CS389_2* conditioned resistance to leaf damage and deadheart damage at Embu and Kiboko. Marker *CS414_2* on chromosome 2 conditioned resistance to exit holes at Kiboko and leaf damage at Embu. Marker *CS190_2* on chromosome 2 conditioned resistance to leaf feeding at Embu and exit holes at Kabete.

Joint analysis of *B. fusca* and *C. partellus* damage and morphological traits across the three sites is presented on Table 13. Four QTLs due to deadheart damage were detected chromosomes 4, 5, 8, and 10 (flanked by markers *BC242-4*, *m05/023.7*, *JK399-8*, and *CPS160-10*, respectively) (Table 13). These explained 2.3% of the phenotypic variation, with individual QTLs accounting for 7.5% – 9%. A simultaneous fit with all the four QTLs based on cross validation explained 24.8% of the adjusted genetic variance. The additive gene action ranged from 3.32 to 16.64. One of the QTLs on chromosome 8 (flanked by marker *JK399-8*) came from the resistant parent (PB 15520-1), while the other three QTLs came from the susceptible parent (ICSV 745).

Six QTLs for leaf feeding damage were detected on markers on chromosomes 1, 3, 6, 7, and 8 (flanked by markers *SB255-1*, *DB160-3*, *DB169-3*, *EF222-6*, *GH70-7*, and *JK290-8*) Table 13). These explained 2.25% of the phenotypic variation, with individual QTLs accounting for 7.6% – 17.4%. A simultaneous fit with all the six QTLs based on cross validation explained 56% of the adjusted genetic variance. The additive gene action ranged from -2.02 to 5.070. Two of the QTLs on chromosomes 3 and 7 (flanked by markers *DB169-3* and *GH70-7*, respectively) came from the resistant parent (PB 15520-1) that contributed to reduce leaf feeding damage, while the other four QTLs came from the susceptible parent (ICSV 745).

Five QTLs for exit hole damage were detected on chromosomes 2, 4, 6, and 9 (flanked by markers *CS195-2*, *BC149-4*, *EF255-6*, *EF416-6*, and *BF138-9*) (Table 13). These explained 5.37% of the phenotypic variation, with individual QTLs accounting for 7% – 11.7%. A simultaneous fit with all the five QTLs based on cross validation explained 47.3% of the adjusted genetic variance. The additive gene action ranged from -0.7 to 3.2. Three of the QTLs on chromosomes 2, 4, and 6 (flanked by markers *CS195-2*, *BC149-4*, and *EF255-6*, respectively) came from the resistant parent (PB 15520-1), while the other two QTLs came from the susceptible parent (ICSV 745).

Six QTLs for stem tunneling damage were detected on chromosomes 1, 2, 3, 4, and 9 (flanked by markers *SB466_1*, *CS116-2*, *CS132-2*, *DB36_3*, *BC140_4*, and *BF140_9*) (Table 13). These explained 5.37% of the phenotypic variation, with individual QTLs accounting for 10.1%–14.7%. A simultaneous fit with all the five QTLs based on cross validation explained 65.5% of the adjusted genetic variance. The additive gene action ranged from -3.4 to 4.4. Two of the QTLs on chromosomes 2 and 9 (flanked by markers *CS132-2* and *BF140_9*, respectively) came from the resistant parent (PB 15520-1), while the other four QTLs came from the susceptible parent (ICSV 745). The analyses further revealed that marker *CS132-2* was colocalised for leaf toughness and stem tunneling traits on QTL 1 and 2, respectively.

## 4. Discussion

Heritability is used to predict response to selection and to help plant breeders know if it is more efficient to improve traits of economic importance through selection [31]. Heritability estimates thus explain the level to which genes control expression of the trait of interest. Heritability estimates for deadheart formation, leaf feeding damage, exit holes, stem tunneling, bloom waxiness, and seedling vigour tested for *B. fusca* at Embu and Kabete were low to high and ranged between 0.21 and 0.65. On the other hand, heritability estimates for the same aforementioned traits evaluated for *C. partellus* at Kiboko were low and ranged between 0.15 and 0.32. This observation suggested that genetic gain may not be realized in selection for some of the traits evaluated for the two borer species in the three sites. These traits that had low heritabilities are as follows: leaf feeding damage at Kabete and Kiboko; deadheart formation, bloom waxiness, and seedling vigour at Kiboko; and exit holes at all the three sites. Therefore, there is a need to consider other alternatives such as DNA marker technology to fix the desired genes. This observation is supported by other researchers. For example, Oloyede-Kamiyo et al. [31] worked on variability for resistance to the pink stem borer (*Sesamia calamistis* Hampson) and the sugarcane borer (*Eldana saccharina* Walker) in two tropical maize populations and observed that narrow-sense heritability was low to moderate and ranged from 1.45% for leaf feeding to 40.6% for stalk breakage.

Path coefficient analyses revealed that leaf feeding, deadheart, exit holes, and stem tunneling had a direct negative effect on grain yield. This observation suggested that either of the damage traits could lead to direct grain yield loss. Singh et al. [6] observed that direct effects of stem tunneling by *C. partellus* on grain yield loss were greater than leaf feeding and deadhearts in sorghum. However, Starks and Doggett [32] reported that leaf feeding damage by *C. partellus* in sorghum is a weak pointer of grain yield as production of new fresh leaves compensate for the leaf damage. Sandoya et al. [33] reported that improvement of maize stem tunneling resistance to the Mediterranean corn borer (*Sesamia nonagrioides* Lef) and the European corn borer (*Ostrinia nubilalis* Hbn) led to increased grain yield. The significant positive correlations observed between leaf damage and deadheart, and between stem tunneling and exit holes, implied that there is a direct relationship between the two traits, respectively, and either of the traits can be used to predict the other in the sorghum population studied. Generally, tall vigorous plants with bloom waxiness produced longer panicle length and high yield with less stem damage. There was a negative relationship between stem tunneling and bloom waxiness and between seedling vigour and deadheart formation implying that sorghum can be bred with either of the morphological traits for reduced damage with high grain yield to the two stem borers.

Colocalization of the markers for some of the traits evaluated was detected on site analysis for *B. fusca* (at Embu and Kabete) and *C. partellus* (at Kiboko). For example, it was observed that the region (flanked by markers *DB172_3*, *EF184_6*, and *BF123_9*) on chromosomes 3, 6, and 9, respectively, conditioned trichome density, deadheart damage, and stem tunneling damage at Kabete for *B. fusca*. This observation implies that the aforementioned traits can be improved by using the same linked markers. Marker *DB169_3* on chromosome 3 conditioned leaf glossiness and deadheart damage at Kabete implying that both traits can be improved by using the same linked marker for *B. fusca*. Marker *CS403_2* on chromosome 2 conditioned seedling vigour and leaf damage for *B. fusca* at Kabete and *C. partellus* at Embu, respectively, implying that both traits can be improved by using the same linked marker. Marker *AD323_5* on chromosome 5 conditioned resistance to deadheart formation as well as seedling vigour at Embu (for *B. fusca*) suggesting that both traits can be improved by using the same linked marker. Marker *CS259_2* on chromosome 2 conditioned resistance to deadheart formation at Kabete (for *B. fusca*) as well as bloom waxiness in Kiboko (for *C. partellus*) implying that the two traits can be improved by using the same linked marker. Marker *GH108_7* on chromosome 7 conditioned resistance to seedling vigour at Kiboko (for *C. partellus*) as well as stem tunneling in Kabete (for *B. fusca*) implying that both traits can be improved by using the same linked marker. Marker *AD323_5* on chromosome 5 conditioned resistance to seedling vigour and deadheart damage at Embu (for *B. fusca*) implying that both traits can be improved by using the same linked marker. Marker *CS389_2* conditioned resistance to leaf damage and deadheart damage at Embu (for *B. fusca*) and Kiboko (for *C. partellus*) implying that both traits can be improved by using the same linked marker. Marker *CS414_2* on chromosome 2 conditioned resistance to exit holes at Kiboko (for *C. partellus*) and leaf damage at Embu (for *B. fusca*) implying that both traits can be improved by using the same linked marker. Marker *CS190_2* on chromosome 2 conditioned resistance to leaf feeding at Embu and exit holes at Kabete implying that both traits can be improved by using the same linked marker for *B. fusca*.

This study identified and mapped genomic regions conditioning dual resistance to *B. fusca* and *C. partellus* in sorghum. The study utilized a large number of RILs and high-density evenly spaced genetic markers scored to ensure high power and precision in QTL mapping as described by Bekele et al. [34]. A five-fold cross validation analysis was run in this study as described by Gowda et al. [30] to confirm the frequency of QTL detection that gave an estimation of the precision of QTL localization. Identification of QTLs that influence dual resistance to *B. fusca* and *C. partellus* would increase the efficiency of selection and breeding for resistance of these two cereal stem borer species. Majority of the identified loci were highly significant, and they accounted for a substantial amount of phenotypic variation for *B. fusca* and *C. partellus.* This implied that fixing these traits (leaf feeding, deadheart formation, exit holes, and stem tunneling damages) would lead to the development of resistant cultivars and thus improve food security since the losses to stem borers will be lowered. A number of reports on linkage and QTL mapping for borer resistance traits have been published in maize [35–37]. QTL mapping for *C. partellus* resistance using the same sorghum mapping population utilized in this study is reported by Vinayan [20]. QTLs governing more than one resistance trait (pleiotropic QTLs) were identified in the present study. For example, it was observed that markers *DB172_3*, *EF184_6*, and *BF123_9* on chromosomes 3, 6, and 9 conditioned trichome density, deadheart damage, and stem tunneling damage at Kabete implying that all these traits can be improved using the same linked markers. Pleotropic QTLs conditioning resistance to borers have been reported in the maize population [38–40].

Chromosome 1 has been associated with multiple resistance to lepidopteran insect pests, mainly, the European corn borer (*Ostrinia nubilalis* (Hubner)), the southwestern corn borer (*Diatrea grandiosella* (Dyar)), and the sugarcane borer (*Diatraea saccharalis* (Fabricius)), and resistance to the maize weevil [41]. Chromosome 4 has been reported to condition resistance to stem tunneling in the tropical maize population against the corn borer [42, 43]. Chromosomes 1, 3, and 8 have been associated with resistance to Mediterranean corn borer *Sesamia nonagrioides* stem tunneling using EP39 × EP42 maize RILs [44]. Chromosomes 1, 2, 6, and 7 in sorghum have also been reported to regulate resistance to deadheart formation against sorghum shoot fly [45]. Joint analyses across the two species of stem borers and the three sites revealed that 4 QTLs conditioned deadheart damage (chr. 4, 5, 10, and 8) and trichome density (chr. 1, 2, 4, and 6); 6 QTLs conditioned leaf feeding damage (chr. 1, 3, 6, 7, and 8), stem tunneling damages (chr. 1, 2, 3, 4, and 9), and leaf toughness (chr. 2, 4, 6, 9, and 10); 5 QTLs conditioned exit holes (chr. 2, 4, 6, and 9); and 2 QTLs conditioned bloom waxiness (chr. 3, and 9), leaf glossiness (chr. 5, and 7), and seedling vigour (chr. 8, and 9). Genomic regions governing trichome density detected on sorghum chromosomes 2, and 6 in this study have not been reported. QTLs identified for trichome density on chromosomes 1 and 3 in the joint analyses in the current study have also been reported in sorghum against shoot fly (*Atherigona soccata* Rond) [8, 45, 46]. Foerster et al. [47] reported that chromosome 9 conditioned bloom waxiness in maize. Aruna et al. [45] reported that chromosome 9 controlled seedling vigour in sorghum against shoot fly. Joint analyses further revealed that marker *CS132-2* colocalised for leaf toughness and stem tunneling on QTLs 1 and 2, respectively, implying that both traits can be improved with the help of the same linked marker.

## 5. Conclusion and Recommendation

This study identified and mapped genomic regions that conditioned expression of dual resistance to *B. fusca* and *C. partellus* in sorghum. QTLs identified in this study can be used in marker-assisted selection in the development of *B. fusca*- and *C. partellus*-resistant sorghum cultivars. Marker *CS132-2* colocalised for leaf toughness and stem tunneling on QTLs 1 and 2, respectively; thus, both traits can be improved for *B. fusca* and *C. partellus* with the help of the same linked marker. There is a need for further studies to identify gene(s) underlying the mapped QTLs.

## Figures and Tables

**Table 1:** Combined analyses of variance across environments.

Source	DF	DH	LD	EH	ST	VG	BW
Rep	1	60.3	1176.8	397.4	5432.6	92.4	20.4
Borer spp.	1	109506.8^∗∗^	417327^∗∗^	701.7^∗∗^	6304.2^∗∗^	5.6^∗^	28.4^∗∗^
Genotype	265	547.1 ns	527.4 ns	137.9^∗∗^	904^∗∗^	1.2^∗∗^	3.2^∗∗^
Environment	3	153767.7^∗∗^	82463.8^∗∗^	4338.2^∗∗^	43160.9^∗∗^	8.1^∗∗^	977.9^∗∗^
Borer_spp · genotype	265	506.9 ns	511.3 ns	60.1^∗∗^	341.2^∗∗^	0.9 ns	2 ns
Genotype · environment	795	323.9 ns	330.5 ns	27 ns	187.4 ns	0.6 ns	1.5 ns
Residual	9302	471.2	585.8	28.9	217.2	0.9	2.1

∗ and ∗∗ indicate that data are significant at the ≤0.05 and ≤0.01 probability levels, respectively; ns=nonsignificant; DF=degrees of freedom; DH=deadheart (%); LD=leaf feeding damage (%); EH=exit holes; ST=stem tunneling; VG=seedling vigour; BW=bloom waxiness; Borer_spp · genotype=borer spp. by genotype interaction; Genotype · environment=genotype by environment interaction.

**Table 2:** Mean squares for *Chilo partellus* damage and morphological traits at the Kiboko site.

Source	DF	DH	LD	EH	ST	VG	BW	GL
Rep	1	9957.3	1276.3	357.74	8260.1	96.6018	136.799	10.3224
Genotype	265	260.5	459.6	80.66^∗∗^	591.3^∗∗^	0.9825^∗∗^	2.598^∗∗^	0.3900^∗∗^
Season	1	395099.3^∗∗^	123.3	12714.94^∗∗^	129477.7^∗∗^	19.058^∗∗^	774.727^∗∗^	1.1911^∗^
Genotype · season	265	262.3	382.6	45.03^∗∗^	352^∗∗^	0.7023	1.567	0.1344
Residual	4786	227.7	450.7	30.52	235.2	0.6442	1.724	0.1915

∗∗ indicates that data is significant at the ≤0.01 probability level; DF=degrees of freedom; DH=deadheart damage; LD=leaf feeding damage; EH=exit holes; ST=stem tunneling; VG=seedling vigour; BW=bloom waxiness; GL=glossiness.

**Table 3:** Mean squares for *Busseola fusca* damage and morphological traits at the Embu site.

Source	DF	DH	LD	EH	ST	VG	GL	BW
Rep	1	18491	5552.1	122.36	594.3	0.1295	3.9774	11.29
Genotype	265	981.6∗∗	945∗∗	65.2∗∗	377.5∗∗	1.6923∗∗	0.5039∗∗	3.94∗∗
Residual	265	440	329.5	22.6	161	0.9548	0.3253	1.828

∗ and ∗∗ indicate that data are significant at the ≤0.05 and ≤0.01 probability levels, respectively; DF=degrees of freedom; DH=deadheart damage; LD=leaf feeding damage; EH=exit holes; ST=stem tunneling; VG=seedling vigour; GL=leaf glossiness; BW=bloom waxiness.

**Table 4:** Mean squares for *Busseola fusca* damage and morphological traits at the Kabete site.

Source	DF	DH	LD	EH	ST	VG	GL	BW
Rep	1	427.1	37498.3	3.99	33	0.5305	3.9774	16.9286
Genotype	265	521.1∗	243.1	88.12∗∗	487.5∗∗	0.60∗∗	0.34∗∗	1.63∗∗
Residual	265	412.3	366.4	28.64	212	0.33	0.2054	0.622

∗ and ∗∗ indicate that data are significant at the ≤0.05 and ≤0.01 probability levels, respectively; DF=degrees of freedom; DH=deadheart damage; LD=leaf feeding damage; EH=exit holes; ST=stem tunneling; VG=seedling vigour; GL=leaf glossiness; BW=bloom waxiness.

**Table 5:** Heritability estimates for stem borer damage and morphological traits at the three sites.

Site	Trait					
	DH	LD	EH	ST	VG	BW
Kabete (*B. fusca*)	0.208	0.651	0.293	0.206	0.436	0.536
Embu (*B. fusca*)	0.551	0.651	0.273	0.212	0.435	0.536
Kiboko (*C. partellus*)	0.199	0.151	0.182	0.172	0.317	0.194

DH=deadheart damage; LD=leaf feeding damage; EH=exit holes; ST=stem tunneling; VG=seedling vigour; BW=bloom waxiness.

**Table 6:** Direct and indirect effects of *B. fusca* damage traits on total grain yield at Embu site.

Character	Correlation with		Indirect effect via			
	Grain yield	Direct effect	DH	EH	LD	ST
DH	-0.96	42.62∗∗	—	-0.0012	-0.0007	0.004
EH	-0.96	4.927∗∗	0.0026	—	0.0132	-0.0325
LD	-0.96	37.62∗∗	0.0005	0.0031	—	-0.0001
ST	-0.96	19.32∗∗	0.0001	0.001	0.0057	—

DH=deadheart damage; EH=number of exit holes; LD=leaf feeding damage; ST=stem tunneling damage; ∗∗ indicates that data is significant at the *P* ≤ 0.01 probability level.

**Table 7:** Direct and indirect effects of *B. fusca* damage traits on total grain yield at Kabete site.

Character	Correlation with		Indirect effect via			
	Grain yield	Direct effect	DH	EH	LD	ST
DH	-0.87	60.37∗∗	—	0.0009	0.0008	0.004
EH	-0.87	4.078∗∗	0.0164	—	0.0012	-0.0066
LD	-0.87	71.24∗∗	0.0018	-0.0006	—	-0.0005
ST	-0.87	18.10∗∗	-0.0053	-0.0071	-0.0039	—

DH=deadheart damage; EH=number of exit holes; LD=leaf feeding damage; ST=stem tunneling damage; ∗∗ indicates that data is significant at the *P* ≤ 0.01 probability level.

**Table 8:** Direct and indirect effects of *C. partellus* damage traits on total grain yield at Kiboko site.

Character	Correlation with		Indirect effect via			
	Grain yield	Direct effect	DH	EH	LD	ST
DH	-0.97	68.11∗∗	—	-0.0006	-0.0061	0.0056
EH	-0.97	6.003∗∗	-0.0015	—	0.0055	0.0161
LD	-0.97	36.93∗∗	-0.0013	-0.0060	—	-0.0003
ST	-0.97	16.69∗∗	0.00149	0.0016	0.0065	—

∗∗ indicates that data is significant at the ≤0.01 probability level; DH=deadheart damage; EH=number of exit holes; LD=leaf feeding damage; ST=stem tunneling damage.

**Table 9:** Correlation coefficients between *B. fusca* damage and agro-morphological traits at Embu site.

PH	—											
DH	-0.03	—										
EH	0.12	0.15∗	—									
FL	0.07	-0.03	0.01	—								
GL	0.05	-0.08	0.01	-0.07	—							
LD	0.06	0.25∗∗	-0.07	-0.01	0.12	—						
PL	0.45∗∗	0.03	0.06	0.10	0.07	0.12	—					
ST	0.18∗∗	0.11	0.71∗∗	0.03	0.01	-0.07	0.12	—				
TR	0.01	0.02	-0.06	0.00	0.18∗∗	0.06	-0.01	-0.09	—			
VG	0.21∗∗	-0.40∗∗	-0.20∗∗	-0.04	0.14∗	-0.01	0.13∗	-0.06	-0.02	—		
BW	0.11	-0.39∗∗	-0.33∗∗	-0.01	0.17∗∗	-0.09	0.07	-0.20∗∗	0.02	0.60∗∗	—	
YLD	0.09	0.08	0.15∗	0.06	-0.07	0.09	0.17∗∗	0.10	0.05	-0.09	-0.06	—
	PH	DH	EH	FL	GL	LD	PL	ST	TR	VG	BW	YLD

∗ and ∗∗ indicate that data are significant at the ≤0.05 and ≤0.01 probability levels, respectively; PH=plant height; DH=deadheart; EH=exit holes; FL=days to flowering; GL=leaf glossiness; LD=leaf damage; PL=panicle length; ST=stem tunneling; TR=trichome density; VG=seedling vigour; BW=bloom waxiness; YLD=total grain yield.

**Table 10:** Correlation coefficients between *B. fusca* damage and agro-morphological traits at Kabete site.

DH	—											
EH	0.05	—										
FL	0.07	-0.13∗	—									
GL	-0.09	-0.09	0.11	—								
LD	0.31∗∗	0.02	0.05	0.01	—							
PH	-0.04	0.10	-0.05	-0.07	-0.01	—						
PL	0.02	0.14∗	-0.03	-0.05	0.11	0.16∗	—					
ST	0.05	0.70∗∗	-0.16∗	-0.11	0.03	0.12	0.15∗	—				
TR	-0.04	0.12	0.02	0.01	-0.05	0.01	-0.05	0.05	—			
VG	-0.38∗∗	-0.15∗	-0.03	0.13∗	-0.01	0.08	-0.14∗	-0.17∗	0.04	—		
BW	-0.09	0.03	0.07	0.20∗∗	0.12	-0.03	0.05	-0.01	0.02	0.15∗	—	
YLD	0.09	0.32∗∗	-0.14∗	-0.09	0.14∗	0.15∗	0.30∗∗	0.27∗∗	-0.07	-0.12	-0.00	—
	DH	EH	FL	GL	LD	PH	PL	ST	TR	VG	BW	YLD

∗ and ∗∗ indicate that data are significant at the ≤0.05 and ≤0.01 probability levels, respectively; PH=plant height; DH=deadheart; EH=exit holes; FL=days to flowering; GL=leaf glossiness; LD=leaf damage; PL=panicle length; ST=stem tunneling; TR=trichome density; VG=seedling vigour; BW=bloom waxiness; YLD=total grain yield.

**Table 11:** Correlation coefficients between *C. partellus* damage and agro-morphological traits at Kiboko site.

DH	—										
EH	-0.10	—									
FL	-0.09	0.01	—								
GL	0.01	0.00	0.02	—							
LD	0.09	-0.01	0.17∗∗	0.05	—						
PH	0.03	0.12	0.13∗	0.04	0.07	—					
PL	-0.02	0.07	0.12	-0.09	-0.05	0.27∗∗	—				
ST	0.02	0.68∗∗	-0.13∗	-0.03	-0.04	0.00	0.03	—			
VG	-0.06	-0.03	-0.33∗∗	-0.03	-0.13∗	-0.00	-0.01	0.13∗	—		
BW	-0.05	-0.09	0.12	0.03	0.04	-0.03	0.13∗	-0.26∗∗	0.13∗	—	
YLD	0.07	0.06	-0.08	-0.01	-0.16∗	0.12	0.34∗∗	0.15∗	0.02	-0.02	—
	DH	EH	FL	GL	LD	PH	PL	ST	VG	BW	YLD

∗ and ∗∗ indicate that data are significant at the ≤0.05 and ≤0.01 probability levels, respectively; PH=plant height; DH=deadheart; EH=exit holes; FL=days to flowering; GL=leaf glossiness; LD=leaf damage; PL=panicle length; ST=stem tunneling; TR=trichome density; VG=seedling vigour; BW=bloom waxiness; YLD=total grain yield.

**Table 12:** QTL analyses at the three sites from the RIL sorghum population derived from ICSV 745 × PB 15520‐1.

Pest species	Site	Trait	QTL	Chr.	Position (cM)	Marker and loci interval	Supp interval	LOD	*R* ^2^	Additive effects	SE	Adj *σ*^2^*g*
*B. fusca*	Embu	DH	1	1	22	SB261_1 251-220	20-26	3.27	7.5	35.13	10.154∗∗	
			2	3	24	DB103_3 1527-1528	22-26	4.11	9.6	-7.79	1.680∗∗	
			3	5	10	AD323_5 2718-2834	8_12	3.97	11.2	8.86	1.731∗∗	28.3
		LD	1	2	4	CS115_2 936-927	2_6	3.11	9.1	-6.48	2.335	
			2	2	84	CS190_2 1011-1172	82-86	3.55	8.1	8.13	1.821∗∗	
			3	2	134	CS389_2 1210-1041	132-136	3.09	7.1	5.27	1.888	
			4	2	148	CS414_2 1235-1083	146-150	3.67	8.7	-8.01	1.687∗∗	
			5	10	30	CPS162_10 4621-4623	26-36	3.26	7.7	7.04	2.323∗∗	40.6
		EH	1	7	26	GH89_7 3380-3379	24-28	3.98	9	-1.42	0.337∗	9.0
		ST	1	7	22	GH87_7 3378-3376	20-24	3.51	8.2	-2.39	0.584∗∗	
			2	9	72	BF123_9 4190-4193	70-74	3.48	8.1	-7.33	1.910∗∗	16.3
		WX	1	3	10	DB39_3 1463-1461	2-12	3.65	14.5	0.471	0.105∗∗	
			2	3	56	DB155_3 1579-1590	54-58	3.17	7.3	0.578	0.112∗∗	
			3	3	144	DB214_3 1638-1637	140-146	3.35	8.1	-2.180	0.469∗	
			4	6	24	EF297_6 3158-3157	22-26	3.26	7.4	-3.888	1.085∗	
			5	9	46	BF101_9 4168-4169	40-48	3.03	7.0	0.844	0.258	29.8
		VG	1	2	70	CS341_2 1162-1168	68-72	3.51	8.0	-0.394	0.101∗∗	
			2	2	70	CS341_2 1162-1168	68-72	4.60	10.5	-0.423	0.092∗∗	
			3	2	140	CS403_2 1224-1064	138-142	3.07	7.1	0.969	0.277∗	
			4	5	10	AD323_5 2718-2834	8-12	3.04	8.7	-0.451	0.077∗	
			5	6	60	EF325_6 3186-3073	58-62	3.06	7.0	-1.578	0.502∗	
			6	7	56	GH99_7 3390-3391	52-60	5.53	12.4	-1.588	0.323∗∗	
			7	7	58	GH100_7 3391-3394	52-60	5.18	11.6	-1.867	0.414∗∗	
			8	7	58	GH100_7 3391-3394	52-60	5.00	11.3	-2.342	0.329∗∗	76.6
		TR	1	2	44	CS129_2 950-953	40-48	3.27	7.5	3.000	1.814	
			2	2	64	CS139_2 960-962	62-66	3.21	7.5	-3.732	0.862∗	
			3	6	52	EF320_6 3181-3068	50-54	3.83	8.8	-14.970	3.786∗∗	23.8
		GL	1	5	14	AD443_5 2838-2676	12-16	3.08	7.5	-0.696	0.225∗∗	
			2	6	62	EF211_6 3072-3194	60-64	3.24	7.4	-0.841	0.221∗∗	14.9

*B. fusca*	Kabete	DH	1	1	28	SB691_1 681-208	26-30	3.53	8.1	12.95	3.602∗∗	
			2	2	138	CS402_2 1223-1224	136-140	3.34	7.6	-7.2	2.177∗∗	
			3	2	154	CS259_2 1080-1099	152-168	4.54	14.6	8.1	1.615∗	
			4	2	82	CS350_2 1171-1011	80-84	3.19	7.3	-8.52	2.359∗	
			5	3	70	DB172_3 1596-1603	68-72	8.16	17.5	-6.08	1.140∗∗	
			6	3	74	DB169_3 1593-1592	72-78	3.91	9	-5.32	1.229∗∗	
			7	4	58	BC149_4 2249-2250	52-60	3.29	7.7	5.77	1.590∗∗	
			8	8	40	JK399_8 4047-3979	38-42	4.38	9.9	-20.68	5.332∗∗	
			9	9	42	BF97_9 4164-4168	38-46	4.96	11.2	9.67	2.373∗∗	
			10	9	56	BF106_9 4173-4179	52-58	5.19	11.7	-14.34	3.455∗∗	80.6
		LD	1	2	140	CS403_2 1224-1064	138-142	3.01	7	-9.54	2.926∗∗	
			2	2	8	CS111_2 932-933	6_10	5.05	12.6	6.56	0.863∗∗	
			3	3	132	CS397_2 1218-1219	130-134	5.41	12.1	4.15	0.902∗∗	
			4	4	64	DB164_3 1588-1598	62-66	4.53	10.2	-3.37	0.834∗∗	
			5	6	72	EF334_6 3195-3079	70-74	3.3	7.6	-14.42	4.207∗∗	
			6	8	18	m08/014.9 3924-3929	14-22	3.17	7.7	-4.3	3.193∗∗	
			7	10	12	CPS158_10 4617-4618	6_16	3.24	9.6	4.07	1.032∗	66.8
		EH	1	2	62	CS342_2 1163-964	60-64	4.26	9.6	1.17	0.266∗∗	
			2	4	84	CS190_2 1011-1172	82-86	4.89	10.9	-1.32	0.284∗∗	
			3	4	72	AD25_5 2420-2343	70-74	7.38	16.5	-2.29	0.434∗∗	
			4	5	28	AD185_5 2580-2701	26-30	3.27	7.8	1.05	0.323∗∗	
			5	6	4	EF255_6 3116-3143	2_6	3.67	8.4	-0.89	0.250∗∗	
			6	9	88	BF138_9 4205-4203	86-90	3.25	7.5	-1.75	0.568∗∗	
			7	8	44	JK208_8 3856-3959	42-46	3.7	8.4	-7.28	0.96	
			8	9	86	BF137_9 4204-4205	84-88	7.13	15.6	-6.49	0.878∗∗	84.7
		ST	1	1	18	SB466_1 456-453	16-20	3.48	8.1	2.7	0.736∗∗	
			2	2	52	CS132_2 953-954	48-58	3.27	7.5	-1.9	0.531∗∗	
			3	2	54	CS133_2 954-955	48-58	3.2	7.4	-2.01	0.563∗∗	
			4	2	90	CS150_2 971-1177	88-92	3.99	9.1	-2.3	0.597∗∗	
			5	3	30	DB140_3 1564-1547	28-32	3.97	4.49	2.68	0.550∗∗	
			6	7	22	GH87_7 3378-3376	20-24	3.04	7.1	-2.1	0.644∗	
			7	3	32	DB143_3 1567-1568	30-34	6.17	13.9	3.98	0.515	
			8	4	46	BC140_4 2240-2229	42-50	3.64	8.5	2.98	0.868∗∗	
			9	7	74	GH108_7 3399-3420	72-76	3.48	8.2	-4.53	1.351∗	74.29
		WX	1	3	36	DB146_3 1570-1571	34-40	5.26	11.9	-0.797	0.069	
			2	5	20	AD290_5 2685-2683	18-22	4.01	9.4	-0.337	0.078	
			3	7	52	GH99_7 3390-3391	50-58	5.43	12.3	0.922	0.219∗∗	33.6
		VG	1	3	58	DB163_3 1587-1588	56-60	3.03	6.9	0.142	0.040∗∗	6.9
		TR	1	3	70	DB172_3 1596-1603	68-72	3.16	7.3	2.171	0.660	
			2	6	16	EF184_6 3045-3161	12-18	3.06	7.1	-2.307	0.721∗∗	
			3	9	72	BF123_9 4190-4193	70-74	7.22	16	-24.470	3.946∗∗	30.4
		GL	1	2	104	CS156_2 977-1188	102-106	3.51	8.0	-0.240	0.070∗	
			2	3	4	DB40_3 1464-1463	2-6	4.17	15.9	0.139	0.029∗	
			3	3	76	DB169_3 1593-1592	74-82	3.20	7.3	0.091	0.028	
			4	6	60	EF325_6 3186-3073	58-62	5.62	12.5	0.874	0.196∗∗	
			5	9	60	BF112_9 4179-4191	58-62	4.19	9.6	-0.193	0.052∗∗	53.3

*C. partellus*	Kiboko	DH	1	2	116	CS369_2 1190-1197	114-118	3.62	8.6	3.32	0.916∗∗	
			2	2	134	CS389_2 1210-1041	132-136	4.99	11.7	-2.71	0.622∗∗	
			3	6	56	EF322_6 3183-3184	54-58	3.49	8.3	-3.17	0.762∗	
			4	9	106	BF152_9 4219-4220	104-108	3.13	12.6	-8.46	.815∗	41.2
		LD	1	2	56	CS133_2 954-955	52-58	3.84	8.7	-3.07	0.758∗	
			2	2	132	CS397_2 1218-1219	130-134	3.78	8.6	-3.06	0.804∗∗	
			3	6	16	EF184_6 3045-3161	14-18	3.14	7.2	2.76	0.801∗∗	24.5
		EH	1	3	50	DB153_3 1577-1580	42-52	3.99	9.1	0.59	0.153∗	
			2	3	134	DB208_3 1632-1635	130-136	3.67	8.5	-1.01	0.287∗∗	
			3	4	40	BC222_4 2322-2239	38-42	3.12	7.2	-0.89	0.255∗	
			4	5	16	m05/015.6 2567-2709	14-18	3.6	8.4	-8.04	2.229∗∗	
			5	2	148	CS414_2 1235-1083	146-150	3.04	7.4	0.61	0.183	
			6	6	4	EF255_6 3116-3143	2_6	3.21	7.6	-0.55	0.172∗∗	
			7	7	16	GH66_7 3357-3367	14-18	4.05	10.3	-0.51	0.148∗	58.5
		ST	1	3	42	DB152_3 1576-1573	40-46	5.08	11.4	2.64	0.391∗	
			2	7	24	GH70_7 3361-3371	22-26	3.4	7.8	-1.83	0.471∗	
			3	7	90	GH118_7 3409-3404	88-94	3.99	9.5	-3.56	0.837	28.7
		WX	1	2	154	CS259_2 1080-1099	152-168	3.88	13.5	-0.631	0.126	13.5
		VG	1	2	122	CS380_2 1201-1205	120-126	3.84	8.7	0.216	0.055∗∗	
			2	6	22	EF303_6 3164-3158	20-24	3.04	7.1	1.546	0.490∗	
			3	7	76	GH108_7 3399-3420	74-78	4.18	9.8	-1.036	0.264∗	
			4	8	20	JK281_8 3929-3930	18-22	3.61	8.6	-0.259	0.068∗∗	
			5	8	20	JK281_8 3929-3930	16-22	3.58	8.7	-0.279	0.073∗∗	
			6	8	20	JK281_8 3929-3930	16-22	4.00	9.6	-0.266	0.073∗∗	52.5
		GL	1	2	104	CS156_2 977-1188	102-106	3.51	8.0	-0.240	0.070∗	
			2	3	4	DB40_3 1464-1463	2-6	4.17	15.9	0.139	0.029∗	
			3	3	76	DB169_3 1593-1592	74-82	3.20	7.3	0.091	0.028	
			4	5	28	AD185_5 2580-2701	26-30	3.15	7.6	-0.168	0.046	
			5	6	60	EF325_6 3186-3073	58-62	5.62	12.5	0.874	0.052∗∗	
			6	6	60	EF325_6 3186-3073	58-62	5.12	11.6	0.867	0.230∗∗	
			7	6	60	EF325_6 3186-3073	58-62	3.19	7.5	0.838	0.223∗∗	
			8	7	22	GH87_7 3378-3376	18-24	3.55	8.3	0.159	0.038∗	
			9	7	36	GH90_7 3381-3382	30-40	3.24	7.5	-0.328	0.110∗	
			10	7	76	GH108_7 3399-3420	74-78	3.27	7.6	-0.541	0.147∗∗	
			11	9	48	BF102_9 4169-4171	44-50	4.08	9.4	-0.287	0.083∗	
			12	9	60	BF112_9 4179-4191	58-62	4.19	9.6	-0.193	0.052∗∗	112.8

QTL=quantitative trait loci; Chr=chromosome; cM=centimorgan; Position=maximum peak in cM, relative to the first locus on each chromosome; Supp interval=supportive interval; LOD=likelihood-ratio test statistic; Additive effect=regression coefficient of the QTL at the specific position from the multiple regression analysis. Positive additive effects indicate that the PB 15520-1 (resistant parent) allele increased the value of the trait; SE=standard error; *R*^2^=coefficient of determination between the respective QTL and the phenotypic observations from the whole data set; Adj *σ*^2^*g*=adjusted genetic variance. ∗ and ∗∗ indicate that data are significant at the ≤0.05 and ≤0.01 probability levels, respectively; DH=deadheart damage; LD=leaf feeding damage; EH=exit holes; ST=stem tunneling; WX=bloom waxiness; VG=seedling vigour; TR=trichome density; GL=leaf glossiness. Marker names in the table were coded for ease of analyses, and their full names are in the supplementary material (see).

**Table 13:** Joint analyses for QTLs common for *B. fusca* and *C. partellus* damage and morphological traits from the RIL population derived from ICSV 745 × PB 15520‐1 sorghum.

Trait	QTL	Chromosome	Position (cM)	Marker and loci interval	Supp interval	LOD	*R* ^2^	Additive effects	SE	Adj *σ*^2^*g*
Deadheart	1	4	74	BC242-4 2342-2347	72-78	3.81	9	16.6	1.587∗	
	2	5	24	m05/023.7 2703-2679	22-26	3.31	7.6	3.3	0.981∗∗	24.8
	3	10	22	CPS160-10 4619-4622	18-28	3.16	8.2	5.4	1.336∗	
	4	8	40	JK399-8 36-42	36-42	3.41	7.8	-9.5	2.758∗	

Leaf damage	1	1	14	SB255-1 245-224	12-16	6.28	15.4	3.8	0.822∗∗	
	2	3	54	DB160-3 1584-1579	52-56	7.88	17.4	5.1	0.657	56
	3	3	74	DB169-3 1593-1592	72-84	3.48	8.1	-2	0.646	
	4	6	76	EF222-6 3083-3203	72-78	3.26	7.6	3.9	1.077	
	5	8	48	JK290-8 3938-3944	46-52	3.24	7.6	2.2	0.705	
	6	7	24	GH70-7 3361-3371	22-26	3.61	8.4	-3.1	0.681∗	

Exit holes	1	2	88	CS195-2 1016-971	86-90	3.94	9	-0.5	0.142∗∗	
	2	4	58	BC149-4 2249-2250	56-60	3.92	9	-0.7	0.167∗∗	47.3
	3	6	4	EF255-6 3116-3143	2-6	5.14	11.7	-0.7	0.154∗∗	
	4	6	26	EF416-6 3277-3281	24-28	4.64	10.5	3.2	0.743∗∗	
	5	9	88	BF138-9 4205-4203	84-90	3	7	1.2	0.361∗∗	

Stem tunneling	1	1	18	SB466_1	456-453	3.55	8.2	1.982	0.523∗	
	2	2	16	CS116-2 937-940	14-18	5.18	11.8	1.6	0.365∗∗	
	3	2	52	CS132-2 953-954	46-56	4.62	10.5	-1.5	0.391∗∗	
	4	3	20	DB36_3 1460-1524	16-24	3.34	10.1	4.4	0.618∗∗	65.5
	5	4	46	BC140_4 2240-2229	42-48	6.57	14.7	3	0.631∗∗	
	6	9	90	BF140_9 4207-4210	88-92	4.44	10.3	-3.4	0.458	

Bloom waxiness	1	3	10	DB39-3 1463-1461	2-12	4.88	19.1	0.5	0.105∗∗	27.3
	2	9	42	BF97-9 4164-4168	40-46	3.52	8.2	1.2	0.201	

Leaf glossiness	1	5	14	AD443-5 2838-2676	12-16	3.32	8.1	-0.7	0.220∗∗	15.2
	2	7	82	GH120-7 3411-3410	80-84	3.01	7.1	-0.5	0.162∗	

Trichome density	1	1	10	SB259-1 249-245	8-12	3.03	7.7	-6.6	1.752	40.5
	2	2	122	CS380-2 1201-1205	120-124	4.66	10.4	-4.1	1.007∗∗	
	3	3	108	DB390-3 1814-1619	102-110	3.19	7.3	4.6	1.428∗	
	4	6	10	EF154-6 3015-3153	8-12	6.90	15.0	-7.8	1.435∗	

Leaf toughness	1	2	56	CS133-2 954-955	54-58	3.01	7.1	0.01	0.006∗	41.1
	2	2	138	CS402-2 1223-1224	136-140	3.23	7.7	0.03	0.011	
	3	4	24	BC131-4 2231-2232	22-26	3.51	8.8	-0.04	0.012∗∗	
	4	6	48	EF204-6 3065-3009	46-50	3.82	8.9	0.1	0.029∗	
	5	9	72	BF123-9 4190-4193	70-74	3.67	8.6	-0.1	0.025∗	
	6	10	64	m10/062.5 4638-4636	60-66	3.22	10.6	0.1	0.011∗∗	

Seedling vigour	1	8	12	JK152-8 3800-3809	8-16	3.05	8.7	0.3	0.099∗∗	19.5
	2	9	20	BF84-9 4151-4149	18-22	4.59	10.8	-0.9	0.076	

QTL=quantitative trait loci; cM=centimorgan; Supp=supportive interval; LOD=likelihood-ratio test statistic; Position=maximum peak in cM, relative to the first locus on each chromosome; Additive effect=regression coefficient of the QTL at the specific position from the multiple regression analysis. Positive additive effects indicate that the PB 15520-1 (resistant parent) allele increased the value of the trait; SE=standard error; *R*^2^=coefficient of determination between the respective QTL and the phenotypic observations from the whole data set; Adj *σ*^2^*g*=adjusted genetic variance. Here, marker names were coded for ease of analyses, and their full names are in the supplementary material (see).

## Data Availability

Some of the data used to support the findings of this study are included in the article. Additional data are available from the corresponding author upon request.

## Supplementary Materials

Supplementary Materials Table 14 Coded markers and their names.
