# Supplementary material for: QTL Mapping of Traits Associated with Dual Resistance to the African Stem Borer (Busseola fusca) and Spotted Stem Borer (Chilo partellus) in Sorghum (Sorghum bicolor)
Source: Int J Genomics. 2021 Jan 15;2021:7016712. doi: 10.1155/2021/7016712 (PMC7834829; doi:10.1155/2021/7016712)
Supplement: Supplementary Materials — Table 14 Coded markers and their names. [file 7016712.f1.docx]

Table 14: showing Coded markers and their names

| Coded name | Marker name |
| --- | --- |
| SB11 | *S1_116694 |
| SB12 | *S1_239795 |
| SB13 | *S1_239798 |
| SB14 | *S1_239802 |
| SB15 | *S1_239807 |
| SB16 | *S1_239810 |
| SB17 | *S1_1067862 |
| SB18 | *S1_1218211 |
| SB19 | *S1_1218250 |
| SB20 | *S1_1232724 |
| SB21 | *S1_1349122 |
| SB22 | *S1_1353046 |
| SB23 | *S1_1419426 |
| SB24 | *S1_1942064 |
| SB25 | *S1_1965871 |
| SB26 | *S1_2077750 |
| SB27 | *S1_2077758 |
| SB28 | *S1_2372533 |
| SB29 | *S1_2372535 |
| SB30 | *S1_2372557 |
| SB31 | *S1_2853564 |
| SB32 | *S1_3320059 |
| SB33 | *S1_3747115 |
| SB34 | *S1_4711600 |
| SB35 | *S1_4897115 |
| SB36 | *S1_5018893 |
| SB37 | *S1_5082794 |
| SB38 | *S1_6028013 |
| SB39 | *S1_6095969 |
| SB40 | *S1_6096542 |
| SB41 | *S1_6682206 |
| SB42 | *S1_7355484 |
| SB43 | *S1_7385618 |
| SB44 | *S1_7430458 |
| SB45 | *S1_7641713 |
| SB46 | *S1_8035365 |
| SB47 | *S1_8035366 |
| SB48 | *S1_9107107 |
| SB49 | *S1_9354241 |
| SB50 | *S1_10064191 |
| SB51 | *S1_10350053 |
| SB52 | *S1_10480907 |
| SB53 | *S1_10480909 |
| SB54 | *S1_10505576 |
| SB55 | *S1_10611728 |
| SB56 | *S1_11106878 |
| SB57 | *S1_11814770 |
| SB58 | *S1_11814810 |
| SB59 | *S1_11824519 |
| SB60 | *S1_12456029 |
| SB61 | *S1_12782615 |
| SB62 | *S1_12939301 |
| SB63 | *S1_12954374 |
| SB64 | *S1_13334172 |
| SB65 | *S1_14616808 |
| SB66 | *S1_14982749 |
| SB67 | *S1_15918710 |
| SB68 | *S1_16567295 |
| SB69 | *S1_17117807 |
| SB70 | *S1_18741898 |
| SB71 | *S1_18988022 |
| SB72 | *S1_19038942 |
| SB73 | *S1_19039268 |
| SB74 | *S1_19039291 |
| SB75 | *S1_19775124 |
| SB76 | *S1_20241382 |
| SB77 | *S1_20278464 |
| SB78 | *S1_20278465 |
| SB79 | *S1_20361801 |
| SB80 | *S1_21306078 |
| SB81 | *S1_21510814 |
| SB82 | *S1_21573608 |
| SB83 | *S1_21637349 |
| SB84 | *S1_21777035 |
| SB85 | *S1_21784980 |
| SB86 | *S1_22221055 |
| SB87 | *S1_22223801 |
| SB88 | *S1_22323789 |
| SB89 | *S1_22323792 |
| SB90 | *S1_24940350 |
| SB91 | *S1_25170241 |
| SB92 | *S1_25816860 |
| SB93 | *S1_26372898 |
| SB94 | *S1_26384690 |
| SB95 | *S1_27138951 |
| SB96 | *S1_27138953 |
| SB97 | *S1_27138954 |
| SB98 | *S1_27652525 |
| SB99 | *S1_27805716 |
| SB100 | *S1_27805735 |
| SB101 | *S1_27805760 |
| SB102 | *S1_29448761 |
| SB103 | *S1_29924340 |
| SB104 | *S1_30757860 |
| SB105 | *S1_30805544 |
| SB106 | *S1_31302211 |
| SB107 | *S1_31338290 |
| SB108 | *S1_42395845 |
| SB109 | *S1_43881166 |
| SB110 | *S1_43881167 |
| SB111 | *S1_44323584 |
| SB112 | *S1_44832538 |
| SB113 | *S1_45707176 |
| SB114 | *S1_46064332 |
| SB115 | *S1_46880058 |
| SB116 | *S1_47079043 |
| SB117 | *S1_47406251 |
| SB118 | *S1_47990264 |
| SB119 | *S1_48512829 |
| SB120 | *S1_49211063 |
| SB121 | *S1_49235253 |
| SB122 | *S1_49640205 |
| SB123 | *S1_49698235 |
| SB124 | *S1_50007964 |
| SB125 | *S1_50095179 |
| SB126 | *S1_50946841 |
| SB127 | *S1_51061412 |
| SB128 | *S1_51468734 |
| SB129 | *S1_51524866 |
| SB130 | *S1_51904631 |
| SB131 | *S1_52251053 |
| SB132 | *S1_52332705 |
| SB133 | *S1_52332752 |
| SB134 | *S1_52345182 |
| SB135 | *S1_52446769 |
| SB136 | *S1_52677725 |
| SB137 | *S1_53199141 |
| SB138 | *S1_53704231 |
| SB139 | *S1_53989212 |
| SB140 | *S1_54197108 |
| SB141 | *S1_54272430 |
| SB142 | *S1_54409202 |
| SB143 | *S1_54721922 |
| SB144 | *S1_56222011 |
| SB145 | *S1_57275295 |
| SB146 | *S1_58017236 |
| SB147 | *S1_58282187 |
| SB148 | *S1_58615698 |
| SB149 | *S1_59451528 |
| SB150 | *S1_59596265 |
| SB151 | *S1_59603519 |
| SB152 | *S1_59663218 |
| SB153 | *S1_59867403 |
| SB154 | *S1_59867451 |
| SB155 | *S1_60091943 |
| SB156 | *S1_60178195 |
| SB157 | *S1_60485456 |
| SB158 | *S1_62162109 |
| SB159 | *S1_62419873 |
| SB160 | *S1_63000156 |
| SB161 | *S1_63000158 |
| SB162 | *S1_65626230 |
| SB163 | *S1_66403832 |
| SB164 | *S1_66455321 |
| SB165 | *S1_66455328 |
| SB166 | *S1_66455331 |
| SB167 | *S1_66597639 |
| SB168 | *S1_67293272 |
| SB169 | *S1_67445433 |
| SB170 | *S1_67494852 |
| SB171 | *S1_67536520 |
| SB172 | *S1_67547957 |
| SB173 | *S1_67615831 |
| SB174 | *S1_67693269 |
| SB175 | *S1_67833367 |
| SB176 | *S1_68338277 |
| SB177 | *S1_68527083 |
| SB178 | *S1_68533147 |
| SB179 | *S1_68936308 |
| SB180 | *S1_70034437 |
| SB181 | *S1_70260209 |
| SB182 | *S1_70265210 |
| SB183 | *S1_70265214 |
| SB184 | *S1_70265216 |
| SB185 | *S1_70265220 |
| SB186 | *S1_70306044 |
| SB187 | *S1_71664493 |
| SB188 | *S1_72078461 |
| SB189 | *S1_72084343 |
| SB190 | *S1_72334273 |
| SB191 | *S1_72362088 |
| SB192 | *S1_72362210 |
| SB193 | *S1_72411604 |
| SB194 | *S1_72411605 |
| SB195 | *S1_72434681 |
| SB196 | *S1_72434686 |
| SB197 | *S1_72434691 |
| SB198 | *S1_72756643 |
| SB199 | *S1_72779563 |
| SB200 | *S1_3747109 |
| SB201 | *S1_198505 |
| SB202 | *S1_59694125 |
| SB203 | *S1_2163 |
| SB204 | *S1_14606 |
| SB205 | *S1_20646 |
| SB206 | *S1_20654 |
| SB207 | *S1_298897 |
| SB208 | *S1_850723 |
| SB209 | *S1_852897 |
| SB210 | *S1_874421 |
| SB211 | *S1_1021125 |
| SB212 | *S1_1349055 |
| SB213 | *S1_1349092 |
| SB214 | *S1_1349124 |
| SB215 | *S1_1391412 |
| SB216 | *S1_1492060 |
| SB217 | *S1_1563363 |
| SB218 | *S1_1563393 |
| SB219 | *S1_1882799 |
| SB220 | *S1_1882804 |
| SB221 | *S1_1883121 |
| SB222 | *S1_1965838 |
| SB223 | *S1_1965883 |
| SB224 | *S1_2083302 |
| SB225 | *S1_2286635 |
| SB226 | *S1_2372537 |
| SB227 | *S1_2372545 |
| SB228 | *S1_2631715 |
| SB229 | *S1_2864117 |
| SB230 | *S1_3299477 |
| SB231 | *S1_3320013 |
| SB232 | *S1_3425338 |
| SB233 | *S1_3515686 |
| SB234 | *S1_3709092 |
| SB235 | *S1_3719550 |
| SB236 | *S1_3719571 |
| SB237 | *S1_3731617 |
| SB238 | *S1_3731652 |
| SB239 | *S1_3876476 |
| SB240 | *S1_3976803 |
| SB241 | *S1_4049199 |
| SB242 | *S1_4053557 |
| SB243 | *S1_4054570 |
| SB244 | *S1_4827790 |
| SB245 | *S1_5107061 |
| SB246 | *S1_5456799 |
| SB247 | *S1_5614535 |
| SB248 | *S1_5721471 |
| SB249 | *S1_6027993 |
| SB250 | *S1_6028018 |
| SB251 | *S1_6095953 |
| SB252 | *S1_6240147 |
| SB253 | *S1_6654995 |
| SB254 | *S1_7517305 |
| SB255 | *S1_7781513 |
| SB256 | *S1_8035165 |
| SB257 | *S1_8078100 |
| SB258 | *S1_8161496 |
| SB259 | *S1_8164558 |
| SB260 | *S1_9107104 |
| SB261 | *S1_9107118 |
| SB262 | *S1_9623601 |
| SB263 | *S1_9776669 |
| SB264 | *S1_9904878 |
| SB265 | *S1_9982701 |
| SB266 | *S1_10097749 |
| SB267 | *S1_10116985 |
| SB268 | *S1_10350048 |
| SB269 | *S1_10480911 |
| SB270 | *S1_11203246 |
| SB271 | *S1_12174767 |
| SB272 | *S1_12269576 |
| SB273 | *S1_12588288 |
| SB274 | *S1_12635922 |
| SB275 | *S1_12782614 |
| SB276 | *S1_12950596 |
| SB277 | *S1_13060993 |
| SB278 | *S1_14292053 |
| SB279 | *S1_14872285 |
| SB280 | *S1_15376106 |
| SB281 | *S1_16478486 |
| SB282 | *S1_17050912 |
| SB283 | *S1_17066099 |
| SB284 | *S1_17788410 |
| SB285 | *S1_17793175 |
| SB286 | *S1_18768740 |
| SB287 | *S1_18966780 |
| SB288 | *S1_19038933 |
| SB289 | *S1_19423446 |
| SB290 | *S1_19564593 |
| SB291 | *S1_20278463 |
| SB292 | *S1_20278473 |
| SB293 | *S1_20278476 |
| SB294 | *S1_20498680 |
| SB295 | *S1_21197570 |
| SB296 | *S1_21306036 |
| SB297 | *S1_21306062 |
| SB298 | *S1_21637169 |
| SB299 | *S1_21657091 |
| SB300 | *S1_21755637 |
| SB301 | *S1_21755663 |
| SB302 | *S1_22323773 |
| SB303 | *S1_22323778 |
| SB304 | *S1_22701075 |
| SB305 | *S1_24940392 |
| SB306 | *S1_25157619 |
| SB307 | *S1_25187657 |
| SB308 | *S1_25271148 |
| SB309 | *S1_25387601 |
| SB310 | *S1_25623637 |
| SB311 | *S1_25816872 |
| SB312 | *S1_27284819 |
| SB313 | *S1_29700878 |
| SB314 | *S1_29786811 |
| SB315 | *S1_31268600 |
| SB316 | *S1_31302210 |
| SB317 | *S1_31302214 |
| SB318 | *S1_43688306 |
| SB319 | *S1_43988439 |
| SB320 | *S1_44811709 |
| SB321 | *S1_45667939 |
| SB322 | *S1_46426298 |
| SB323 | *S1_46426350 |
| SB324 | *S1_46426353 |
| SB325 | *S1_46722722 |
| SB326 | *S1_46831647 |
| SB327 | *S1_46880052 |
| SB328 | *S1_47079049 |
| SB329 | *S1_47134707 |
| SB330 | *S1_47184186 |
| SB331 | *S1_47401434 |
| SB332 | *S1_47990222 |
| SB333 | *S1_47990278 |
| SB334 | *S1_48437827 |
| SB335 | *S1_48463954 |
| SB336 | *S1_48758499 |
| SB337 | *S1_48758520 |
| SB338 | *S1_49322823 |
| SB339 | *S1_49663877 |
| SB340 | *S1_49676916 |
| SB341 | *S1_51076146 |
| SB342 | *S1_51826712 |
| SB343 | *S1_51909365 |
| SB344 | *S1_52020280 |
| SB345 | *S1_52201747 |
| SB346 | *S1_52258392 |
| SB347 | *S1_52332709 |
| SB348 | *S1_52332748 |
| SB349 | *S1_52332749 |
| SB350 | *S1_52564632 |
| SB351 | *S1_52571550 |
| SB352 | *S1_52767935 |
| SB353 | *S1_52867305 |
| SB354 | *S1_53685883 |
| SB355 | *S1_53989208 |
| SB356 | *S1_54009140 |
| SB357 | *S1_54197122 |
| SB358 | *S1_54197130 |
| SB359 | *S1_54217574 |
| SB360 | *S1_54416925 |
| SB361 | *S1_54475026 |
| SB362 | *S1_54538244 |
| SB363 | *S1_55090390 |
| SB364 | *S1_55130501 |
| SB365 | *S1_55177418 |
| SB366 | *S1_55421116 |
| SB367 | *S1_57604127 |
| SB368 | *S1_57629291 |
| SB369 | *S1_59139438 |
| SB370 | *S1_59150809 |
| SB371 | *S1_59430447 |
| SB372 | *S1_59603497 |
| SB373 | *S1_59702549 |
| SB374 | *S1_59717983 |
| SB375 | *S1_60256146 |
| SB376 | *S1_60362003 |
| SB377 | *S1_60383946 |
| SB378 | *S1_60485478 |
| SB379 | *S1_60664068 |
| SB380 | *S1_62365544 |
| SB381 | *S1_62828049 |
| SB382 | *S1_63000157 |
| SB383 | *S1_63000159 |
| SB384 | *S1_63025533 |
| SB385 | *S1_64618559 |
| SB386 | *S1_64862304 |
| SB387 | *S1_65357022 |
| SB388 | *S1_65419873 |
| SB389 | *S1_65419874 |
| SB390 | *S1_65419877 |
| SB391 | *S1_65561758 |
| SB392 | *S1_65573616 |
| SB393 | *S1_65574884 |
| SB394 | *S1_65574908 |
| SB395 | *S1_65607259 |
| SB396 | *S1_65884848 |
| SB397 | *S1_66237086 |
| SB398 | *S1_66237143 |
| SB399 | *S1_66245351 |
| SB400 | *S1_66529060 |
| SB401 | *S1_66572689 |
| SB402 | *S1_66633184 |
| SB403 | *S1_66701363 |
| SB404 | *S1_66975576 |
| SB405 | *S1_67350900 |
| SB406 | *S1_67801097 |
| SB407 | *S1_68279667 |
| SB408 | *S1_68338270 |
| SB409 | *S1_68338272 |
| SB410 | *S1_68338275 |
| SB411 | *S1_68338279 |
| SB412 | *S1_68646897 |
| SB413 | *S1_68785401 |
| SB414 | *S1_69657996 |
| SB415 | *S1_70260253 |
| SB416 | *S1_70265217 |
| SB417 | *S1_70306043 |
| SB418 | *S1_70788029 |
| SB419 | *S1_70828309 |
| SB420 | *S1_70874710 |
| SB421 | *S1_71546773 |
| SB422 | *S1_71560783 |
| SB423 | *S1_72123469 |
| SB424 | *S1_72217669 |
| SB425 | *S1_72222769 |
| SB426 | *S1_72362087 |
| SB427 | *S1_73245460 |
| SB428 | *S1_73582682 |
| SB429 | *S1_378289 |
| SB430 | *S1_831577 |
| SB431 | *S1_831628 |
| SB432 | *S1_874377 |
| SB433 | *S1_922635 |
| SB434 | *S1_1223474 |
| SB435 | *S1_1302191 |
| SB436 | *S1_1349009 |
| SB437 | *S1_1349119 |
| SB438 | *S1_1349121 |
| SB439 | *S1_1349125 |
| SB440 | *S1_1501571 |
| SB441 | *S1_1563344 |
| SB442 | *S1_1563396 |
| SB443 | *S1_1857153 |
| SB444 | *S1_1882798 |
| SB445 | *S1_1882823 |
| SB446 | *S1_1883113 |
| SB447 | *S1_1942067 |
| SB448 | *S1_1942068 |
| SB449 | *S1_1942071 |
| SB450 | *S1_2021260 |
| SB451 | *S1_2077731 |
| SB452 | *S1_2372544 |
| SB453 | *S1_3107006 |
| SB454 | *S1_3506414 |
| SB455 | *S1_4201399 |
| SB456 | *S1_4227308 |
| SB457 | *S1_4372721 |
| SB458 | *S1_5593407 |
| SB459 | *S1_6007223 |
| SB460 | *S1_6095970 |
| SB461 | *S1_6098935 |
| SB462 | *S1_7310433 |
| SB463 | *S1_7571071 |
| SB464 | *S1_7601644 |
| SB465 | *S1_7641717 |
| SB466 | *S1_7660099 |
| SB467 | *S1_7781554 |
| SB468 | *S1_8078071 |
| SB469 | *S1_8844678 |
| SB470 | *S1_9623604 |
| SB471 | *S1_9623629 |
| SB472 | *S1_9832514 |
| SB473 | *S1_10410788 |
| SB474 | *S1_10480906 |
| SB475 | *S1_10480908 |
| SB476 | *S1_10480910 |
| SB477 | *S1_10611713 |
| SB478 | *S1_11354916 |
| SB479 | *S1_11421564 |
| SB480 | *S1_11814727 |
| SB481 | *S1_11956703 |
| SB482 | *S1_12732909 |
| SB483 | *S1_12950557 |
| SB484 | *S1_12950595 |
| SB485 | *S1_14262331 |
| SB486 | *S1_14660024 |
| SB487 | *S1_15723195 |
| SB488 | *S1_16806773 |
| SB489 | *S1_17829241 |
| SB490 | *S1_17986943 |
| SB491 | *S1_19038949 |
| SB492 | *S1_19760428 |
| SB493 | *S1_20278462 |
| SB494 | *S1_20278466 |
| SB495 | *S1_20278479 |
| SB496 | *S1_20278482 |
| SB497 | *S1_20278483 |
| SB498 | *S1_20320885 |
| SB499 | *S1_20321046 |
| SB500 | *S1_21188136 |
| SB501 | *S1_21197026 |
| SB502 | *S1_21517081 |
| SB503 | *S1_21517099 |
| SB504 | *S1_21550210 |
| SB505 | *S1_21777039 |
| SB506 | *S1_21777208 |
| SB507 | *S1_21945581 |
| SB508 | *S1_22114506 |
| SB509 | *S1_22323777 |
| SB510 | *S1_22323787 |
| SB511 | *S1_22323798 |
| SB512 | *S1_22359553 |
| SB513 | *S1_25157415 |
| SB514 | *S1_25262472 |
| SB515 | *S1_25271128 |
| SB516 | *S1_25271153 |
| SB517 | *S1_25271154 |
| SB518 | *S1_25282683 |
| SB519 | *S1_25387627 |
| SB520 | *S1_26019879 |
| SB521 | *S1_26372710 |
| SB522 | *S1_26372890 |
| SB523 | *S1_27293120 |
| SB524 | *S1_27805731 |
| SB525 | *S1_27805765 |
| SB526 | *S1_29448723 |
| SB527 | *S1_30614665 |
| SB528 | *S1_43819202 |
| SB529 | *S1_43881171 |
| SB530 | *S1_45706276 |
| SB531 | *S1_45762134 |
| SB532 | *S1_47079050 |
| SB533 | *S1_47079055 |
| SB534 | *S1_47401471 |
| SB535 | *S1_47496589 |
| SB536 | *S1_47819692 |
| SB537 | *S1_47990379 |
| SB538 | *S1_48451035 |
| SB539 | *S1_48451038 |
| SB540 | *S1_48451044 |
| SB541 | *S1_48463961 |
| SB542 | *S1_48784860 |
| SB543 | *S1_49117544 |
| SB544 | *S1_49235307 |
| SB545 | *S1_49369553 |
| SB546 | *S1_49640230 |
| SB547 | *S1_49663878 |
| SB548 | *S1_49663887 |
| SB549 | *S1_50007923 |
| SB550 | *S1_50268184 |
| SB551 | *S1_50614823 |
| SB552 | *S1_51089220 |
| SB553 | *S1_51300982 |
| SB554 | *S1_51302818 |
| SB555 | *S1_51415401 |
| SB556 | *S1_52020282 |
| SB557 | *S1_52020284 |
| SB558 | *S1_52056855 |
| SB559 | *S1_52219108 |
| SB560 | *S1_52219113 |
| SB561 | *S1_52332733 |
| SB562 | *S1_52332750 |
| SB563 | *S1_52419904 |
| SB564 | *S1_52571584 |
| SB565 | *S1_52575421 |
| SB566 | *S1_53217167 |
| SB567 | *S1_53989254 |
| SB568 | *S1_54118125 |
| SB569 | *S1_54197123 |
| SB570 | *S1_54197146 |
| SB571 | *S1_55090342 |
| SB572 | *S1_55302855 |
| SB573 | *S1_55421115 |
| SB574 | *S1_57352754 |
| SB575 | *S1_57352769 |
| SB576 | *S1_58118978 |
| SB577 | *S1_58197319 |
| SB578 | *S1_58322828 |
| SB579 | *S1_58615750 |
| SB580 | *S1_59180099 |
| SB581 | *S1_59181792 |
| SB582 | *S1_59441288 |
| SB583 | *S1_59659575 |
| SB584 | *S1_59663203 |
| SB585 | *S1_60091815 |
| SB586 | *S1_60485454 |
| SB587 | *S1_60485459 |
| SB588 | *S1_60485460 |
| SB589 | *S1_60563533 |
| SB590 | *S1_60664070 |
| SB591 | *S1_62162124 |
| SB592 | *S1_62233061 |
| SB593 | *S1_62899537 |
| SB594 | *S1_62933790 |
| SB595 | *S1_65407158 |
| SB596 | *S1_65419866 |
| SB597 | *S1_66237085 |
| SB598 | *S1_66245282 |
| SB599 | *S1_66245308 |
| SB600 | *S1_66455322 |
| SB601 | *S1_66455326 |
| SB602 | *S1_66455327 |
| SB603 | *S1_66455332 |
| SB604 | *S1_66626579 |
| SB605 | *S1_66627040 |
| SB606 | *S1_66633183 |
| SB607 | *S1_66738405 |
| SB608 | *S1_66769372 |
| SB609 | *S1_66813762 |
| SB610 | *S1_67389904 |
| SB611 | *S1_67775915 |
| SB612 | *S1_67896786 |
| SB613 | *S1_68230485 |
| SB614 | *S1_68328827 |
| SB615 | *S1_68338273 |
| SB616 | *S1_68533142 |
| SB617 | *S1_68535079 |
| SB618 | *S1_68785423 |
| SB619 | *S1_69205182 |
| SB620 | *S1_69449976 |
| SB621 | *S1_69564907 |
| SB622 | *S1_70105500 |
| SB623 | *S1_70221903 |
| SB624 | *S1_70240828 |
| SB625 | *S1_70265208 |
| SB626 | *S1_70265209 |
| SB627 | *S1_70265211 |
| SB628 | *S1_70265215 |
| SB629 | *S1_70265219 |
| SB630 | *S1_70306018 |
| SB631 | *S1_70739754 |
| SB632 | *S1_70751335 |
| SB633 | *S1_71024684 |
| SB634 | *S1_71838695 |
| SB635 | *S1_71845946 |
| SB636 | *S1_72334230 |
| SB637 | *S1_72411603 |
| SB638 | *S1_72411606 |
| SB639 | *S1_72411645 |
| SB640 | *S1_72902294 |
| SB641 | *S1_73640068 |
| SB642 | *S1_73662423 |
| SB643 | *S1_29532878 |
| SB644 | *S1_20601 |
| SB645 | *S1_197958 |
| SB646 | *S1_239794 |
| SB647 | *S1_239813 |
| SB648 | *S1_958421 |
| SB649 | *S1_958422 |
| SB650 | *S1_958456 |
| SB651 | *S1_1021115 |
| SB652 | *S1_1563397 |
| SB653 | *S1_1623831 |
| SB654 | *S1_1857144 |
| SB655 | *S1_1883114 |
| SB656 | *S1_1883117 |
| SB657 | *S1_1883118 |
| SB658 | *S1_1883119 |
| SB659 | *S1_1883120 |
| SB660 | *S1_1942069 |
| SB661 | *S1_2193264 |
| SB662 | *S1_2320744 |
| SB663 | *S1_2372536 |
| SB664 | *S1_2864116 |
| SB665 | *S1_3071286 |
| SB666 | *S1_3171807 |
| SB667 | *S1_3515687 |
| SB668 | *S1_3546465 |
| SB669 | *S1_3580938 |
| SB670 | *S1_3881933 |
| SB671 | *S1_4007628 |
| SB672 | *S1_4297874 |
| SB673 | *S1_5027354 |
| SB674 | *S1_5064933 |
| SB675 | *S1_5943062 |
| SB676 | *S1_5950409 |
| SB677 | *S1_6028003 |
| SB678 | *S1_6055511 |
| SB679 | *S1_6112927 |
| SB680 | *S1_7310434 |
| SB681 | *S1_7320214 |
| SB682 | *S1_7385621 |
| SB683 | *S1_7846963 |
| SB684 | *S1_8041382 |
| SB685 | *S1_9107116 |
| SB686 | *S1_9600460 |
| SB687 | *S1_9858793 |
| SB688 | *S1_9982866 |
| SB689 | *S1_10480905 |
| SB690 | *S1_11004597 |
| SB691 | *S1_11608350 |
| SB692 | *S1_11814748 |
| SB693 | *S1_11969857 |
| SB694 | *S1_12171176 |
| SB695 | *S1_12689933 |
| SB696 | *S1_12705968 |
| SB697 | *S1_12733940 |
| SB698 | *S1_12950547 |
| SB699 | *S1_12950560 |
| SB700 | *S1_14872327 |
| SB701 | *S1_15899571 |
| SB702 | *S1_15918722 |
| SB703 | *S1_15918761 |
| SB704 | *S1_16567282 |
| SB705 | *S1_16633467 |
| SB706 | *S1_17050920 |
| SB707 | *S1_17682749 |
| SB708 | *S1_17948478 |
| SB709 | *S1_18119283 |
| SB710 | *S1_18139285 |
| SB711 | *S1_18502196 |
| SB712 | *S1_18927750 |
| SB713 | *S1_19100348 |
| SB714 | *S1_19423456 |
| SB715 | *S1_20278467 |
| SB716 | *S1_20278469 |
| SB717 | *S1_20278478 |
| SB718 | *S1_20320847 |
| SB719 | *S1_20410220 |
| SB720 | *S1_21306058 |
| SB721 | *S1_21784956 |
| SB722 | *S1_21939977 |
| SB723 | *S1_22094143 |
| SB724 | *S1_22323774 |
| SB725 | *S1_22323790 |
| SB726 | *S1_22323797 |
| SB727 | *S1_22323800 |
| SB728 | *S1_24940349 |
| SB729 | *S1_25055554 |
| SB730 | *S1_25170354 |
| SB731 | *S1_25271157 |
| SB732 | *S1_25282678 |
| SB733 | *S1_25285099 |
| SB734 | *S1_25373384 |
| SB735 | *S1_25384795 |
| SB736 | *S1_27138950 |
| SB737 | *S1_27677313 |
| SB738 | *S1_29448755 |
| SB739 | *S1_29700883 |
| SB740 | *S1_29786850 |
| SB741 | *S1_31275346 |
| SB742 | *S1_42395883 |
| SB743 | *S1_42890637 |
| SB744 | *S1_43881170 |
| SB745 | *S1_44558312 |
| SB746 | *S1_45309480 |
| SB747 | *S1_45667919 |
| SB748 | *S1_45667956 |
| SB749 | *S1_45683146 |
| SB750 | *S1_45776005 |
| SB751 | *S1_46049154 |
| SB752 | *S1_46722728 |
| SB753 | *S1_47278382 |
| SB754 | *S1_47447069 |
| SB755 | *S1_47658011 |
| SB756 | *S1_48088610 |
| SB757 | *S1_48510972 |
| SB758 | *S1_49064455 |
| SB759 | *S1_49211027 |
| SB760 | *S1_49640204 |
| SB761 | *S1_49676893 |
| SB762 | *S1_49676911 |
| SB763 | *S1_49759993 |
| SB764 | *S1_49759994 |
| SB765 | *S1_49891961 |
| SB766 | *S1_50840222 |
| SB767 | *S1_50946840 |
| SB768 | *S1_51061409 |
| SB769 | *S1_51061411 |
| SB770 | *S1_51877637 |
| SB771 | *S1_51877643 |
| SB772 | *S1_51909361 |
| SB773 | *S1_52003698 |
| SB774 | *S1_52003699 |
| SB775 | *S1_52020283 |
| SB776 | *S1_52219110 |
| SB777 | *S1_52419922 |
| SB778 | *S1_53775506 |
| SB779 | *S1_54021725 |
| SB780 | *S1_54197116 |
| SB781 | *S1_54241545 |
| SB782 | *S1_55177391 |
| SB783 | *S1_55281829 |
| SB784 | *S1_55361750 |
| SB785 | *S1_55801286 |
| SB786 | *S1_56844925 |
| SB787 | *S1_56849399 |
| SB788 | *S1_56963463 |
| SB789 | *S1_57821154 |
| SB790 | *S1_57891447 |
| SB791 | *S1_58118943 |
| SB792 | *S1_59108389 |
| SB793 | *S1_59126575 |
| SB794 | *S1_59139449 |
| SB795 | *S1_59139495 |
| SB796 | *S1_59447711 |
| SB797 | *S1_59570642 |
| SB798 | *S1_59647691 |
| SB799 | *S1_59949045 |
| SB800 | *S1_60266488 |
| SB801 | *S1_60361936 |
| SB802 | *S1_60485434 |
| SB803 | *S1_60485457 |
| SB804 | *S1_60485481 |
| SB805 | *S1_60507732 |
| SB806 | *S1_62389927 |
| SB807 | *S1_62441638 |
| SB808 | *S1_62574013 |
| SB809 | *S1_62802920 |
| SB810 | *S1_65384607 |
| SB811 | *S1_65394756 |
| SB812 | *S1_65419865 |
| SB813 | *S1_65419867 |
| SB814 | *S1_65573619 |
| SB815 | *S1_66331038 |
| SB816 | *S1_66455324 |
| SB817 | *S1_66500711 |
| SB818 | *S1_66567401 |
| SB819 | *S1_66577222 |
| SB820 | *S1_66680876 |
| SB821 | *S1_67207924 |
| SB822 | *S1_67405191 |
| SB823 | *S1_67445418 |
| SB824 | *S1_68312282 |
| SB825 | *S1_68437188 |
| SB826 | *S1_69449954 |
| SB827 | *S1_70173566 |
| SB828 | *S1_70282647 |
| SB829 | *S1_70306020 |
| SB830 | *S1_70739716 |
| SB831 | *S1_71080285 |
| SB832 | *S1_71314857 |
| SB833 | *S1_71778158 |
| SB834 | *S1_71862841 |
| SB835 | *S1_72411607 |
| SB836 | *S1_72473385 |
| SB837 | *S1_73309024 |
| SB838 | *S1_73544709 |
| SB839 | *S1_73805243 |
| SB840 | *S1_3747110 |
| CS10 | *S2_673587 |
| CS11 | *S2_1048815 |
| CS12 | *S2_1275051 |
| CS13 | *S2_1328630 |
| CS14 | *S2_1571952 |
| CS15 | *S2_1656396 |
| CS16 | *S2_2203378 |
| CS17 | *S2_3098538 |
| CS18 | *S2_3389213 |
| CS19 | *S2_3785890 |
| CS20 | *S2_3791534 |
| CS21 | *S2_3897555 |
| CS22 | *S2_4167832 |
| CS23 | *S2_4168074 |
| CS24 | *S2_4347542 |
| CS25 | *S2_4396379 |
| CS26 | *S2_4574836 |
| CS27 | *S2_4825976 |
| CS28 | *S2_4916907 |
| CS29 | *S2_5316188 |
| CS30 | *S2_5579692 |
| CS31 | *S2_5800751 |
| CS32 | *S2_5954094 |
| CS33 | *S2_6033898 |
| CS34 | *S2_6145481 |
| CS35 | *S2_6153923 |
| CS36 | *S2_6220828 |
| CS37 | *S2_6235434 |
| CS38 | *S2_6246379 |
| CS39 | *S2_6264759 |
| CS40 | *S2_6700069 |
| CS41 | *S2_6707205 |
| CS42 | *S2_7470908 |
| CS43 | *S2_7715809 |
| CS44 | *S2_7739142 |
| CS45 | *S2_8203510 |
| CS46 | *S2_9111032 |
| CS47 | *S2_9408375 |
| CS48 | *S2_9668228 |
| CS49 | *S2_9997554 |
| CS50 | *S2_10026359 |
| CS51 | *S2_10026362 |
| CS52 | *S2_10026369 |
| CS53 | *S2_10026371 |
| CS54 | *S2_10090659 |
| CS55 | *S2_10343957 |
| CS56 | *S2_10343964 |
| CS57 | *S2_10343989 |
| CS58 | *S2_10379619 |
| CS59 | *S2_10428234 |
| CS60 | *S2_10448509 |
| CS61 | *S2_11118921 |
| CS62 | *S2_11129603 |
| CS63 | *S2_11373304 |
| CS64 | *S2_11450729 |
| CS65 | *S2_11554883 |
| CS66 | *S2_11587152 |
| CS67 | *S2_11587278 |
| CS68 | *S2_12741167 |
| CS69 | *S2_16549739 |
| CS70 | *S2_16900994 |
| CS71 | *S2_16901024 |
| CS72 | *S2_16918345 |
| CS73 | *S2_17814140 |
| CS74 | *S2_18830678 |
| CS75 | *S2_19576038 |
| CS76 | *S2_19764836 |
| CS77 | *S2_20044223 |
| CS78 | *S2_20187819 |
| CS79 | *S2_21727350 |
| CS80 | *S2_22347404 |
| CS81 | *S2_23130089 |
| CS82 | *S2_23366103 |
| CS83 | *S2_28987533 |
| CS84 | *S2_34965640 |
| CS85 | *S2_35162825 |
| CS86 | *S2_40150274 |
| CS87 | *S2_40251908 |
| CS88 | *S2_43818848 |
| CS89 | *S2_46756518 |
| CS90 | *S2_52403691 |
| CS91 | *S2_55514069 |
| CS92 | *S2_56198764 |
| CS93 | *S2_56439901 |
| CS94 | *S2_56763936 |
| CS95 | *S2_57511418 |
| CS96 | *S2_57556756 |
| CS97 | *S2_58430563 |
| CS98 | *S2_58607679 |
| CS99 | *S2_58608746 |
| CS100 | *S2_58639230 |
| CS101 | *S2_58867252 |
| CS102 | *S2_58949708 |
| CS103 | *S2_59135058 |
| CS104 | *S2_59326717 |
| CS105 | *S2_59821920 |
| CS106 | *S2_59821923 |
| CS107 | *S2_59915393 |
| CS108 | *S2_60978892 |
| CS109 | *S2_61131430 |
| CS110 | *S2_61131438 |
| CS111 | *S2_61134839 |
| CS112 | *S2_61279469 |
| CS113 | *S2_61614526 |
| CS114 | *S2_61713566 |
| CS115 | *S2_61757557 |
| CS116 | *S2_61763974 |
| CS117 | *S2_61811694 |
| CS118 | *S2_62046647 |
| CS119 | *S2_62072573 |
| CS120 | *S2_63413403 |
| CS121 | *S2_65006997 |
| CS122 | *S2_65124571 |
| CS123 | *S2_66713117 |
| CS124 | *S2_67011661 |
| CS125 | *S2_70002347 |
| CS126 | *S2_70052730 |
| CS127 | *S2_70067680 |
| CS128 | *S2_70238683 |
| CS129 | *S2_70238704 |
| CS130 | *S2_70290073 |
| CS131 | *S2_70855067 |
| CS132 | *S2_72395038 |
| CS133 | *S2_72850011 |
| CS134 | *S2_73134755 |
| CS135 | *S2_73175119 |
| CS136 | *S2_73721716 |
| CS137 | *S2_73721726 |
| CS138 | *S2_73826237 |
| CS139 | *S2_73872731 |
| CS140 | *S2_73990924 |
| CS141 | *S2_75037606 |
| CS142 | *S2_75181633 |
| CS143 | *S2_75181634 |
| CS144 | *S2_75525227 |
| CS145 | *S2_75648177 |
| CS146 | *S2_75866439 |
| CS147 | *S2_76939105 |
| CS148 | *S2_77197351 |
| CS149 | *S2_77197353 |
| CS150 | *S2_77334383 |
| CS151 | *S2_77667149 |
| CS152 | *S2_3638977 |
| CS153 | *S2_72826063 |
| CS154 | *S2_470762 |
| CS155 | *S2_500824 |
| CS156 | *S2_1404060 |
| CS157 | *S2_1674987 |
| CS158 | *S2_2195341 |
| CS159 | *S2_3080623 |
| CS160 | *S2_3080636 |
| CS161 | *S2_3389183 |
| CS162 | *S2_3785931 |
| CS163 | *S2_3897184 |
| CS164 | *S2_3897557 |
| CS165 | *S2_3947115 |
| CS166 | *S2_3947124 |
| CS167 | *S2_3962348 |
| CS168 | *S2_3967112 |
| CS169 | *S2_4167834 |
| CS170 | *S2_4283999 |
| CS171 | *S2_4284041 |
| CS172 | *S2_4594407 |
| CS173 | *S2_4942984 |
| CS174 | *S2_5297187 |
| CS175 | *S2_5392150 |
| CS176 | *S2_5715277 |
| CS177 | *S2_5852240 |
| CS178 | *S2_5954052 |
| CS179 | *S2_6025087 |
| CS180 | *S2_6029515 |
| CS181 | *S2_6220836 |
| CS182 | *S2_6232331 |
| CS183 | *S2_6280279 |
| CS184 | *S2_6284752 |
| CS185 | *S2_6284753 |
| CS186 | *S2_6700075 |
| CS187 | *S2_6829359 |
| CS188 | *S2_6829395 |
| CS189 | *S2_7804399 |
| CS190 | *S2_8116926 |
| CS191 | *S2_8321488 |
| CS192 | *S2_9107416 |
| CS193 | *S2_9403775 |
| CS194 | *S2_9557525 |
| CS195 | *S2_9597951 |
| CS196 | *S2_9631067 |
| CS197 | *S2_9700964 |
| CS198 | *S2_10026370 |
| CS199 | *S2_10026387 |
| CS200 | *S2_10026394 |
| CS201 | *S2_10090611 |
| CS202 | *S2_10343977 |
| CS203 | *S2_10380099 |
| CS204 | *S2_10448508 |
| CS205 | *S2_11587296 |
| CS206 | *S2_11937479 |
| CS207 | *S2_11964609 |
| CS208 | *S2_12061899 |
| CS209 | *S2_12470990 |
| CS210 | *S2_12733173 |
| CS211 | *S2_16669014 |
| CS212 | *S2_16900968 |
| CS213 | *S2_16918344 |
| CS214 | *S2_17129741 |
| CS215 | *S2_17129764 |
| CS216 | *S2_18922623 |
| CS217 | *S2_19271787 |
| CS218 | *S2_19530777 |
| CS219 | *S2_19530838 |
| CS220 | *S2_19764840 |
| CS221 | *S2_23150453 |
| CS222 | *S2_26094811 |
| CS223 | *S2_28681483 |
| CS224 | *S2_29965175 |
| CS225 | *S2_31368393 |
| CS226 | *S2_31667543 |
| CS227 | *S2_33060309 |
| CS228 | *S2_37822232 |
| CS229 | *S2_38876856 |
| CS230 | *S2_39957667 |
| CS231 | *S2_39992122 |
| CS232 | *S2_40397620 |
| CS233 | *S2_40397632 |
| CS234 | *S2_41376248 |
| CS235 | *S2_43818831 |
| CS236 | *S2_45873104 |
| CS237 | *S2_46682400 |
| CS238 | *S2_46774741 |
| CS239 | *S2_52429204 |
| CS240 | *S2_54516283 |
| CS241 | *S2_55425054 |
| CS242 | *S2_55425940 |
| CS243 | *S2_55514033 |
| CS244 | *S2_55600794 |
| CS245 | *S2_56198758 |
| CS246 | *S2_56198762 |
| CS247 | *S2_56338794 |
| CS248 | *S2_56444739 |
| CS249 | *S2_57517513 |
| CS250 | *S2_57703534 |
| CS251 | *S2_57726523 |
| CS252 | *S2_57764975 |
| CS253 | *S2_58607699 |
| CS254 | *S2_59244014 |
| CS255 | *S2_59727158 |
| CS256 | *S2_59734270 |
| CS257 | *S2_60978893 |
| CS258 | *S2_60978913 |
| CS259 | *S2_60999422 |
| CS260 | *S2_61614537 |
| CS261 | *S2_61615819 |
| CS262 | *S2_61621292 |
| CS263 | *S2_61713574 |
| CS264 | *S2_62040827 |
| CS265 | *S2_62046640 |
| CS266 | *S2_62070391 |
| CS267 | *S2_63941736 |
| CS268 | *S2_65006998 |
| CS269 | *S2_65124589 |
| CS270 | *S2_66594076 |
| CS271 | *S2_66713112 |
| CS272 | *S2_67686143 |
| CS273 | *S2_67957403 |
| CS274 | *S2_70020660 |
| CS275 | *S2_70020684 |
| CS276 | *S2_70067666 |
| CS277 | *S2_70067670 |
| CS278 | *S2_70199001 |
| CS279 | *S2_70238684 |
| CS280 | *S2_70290108 |
| CS281 | *S2_71063557 |
| CS282 | *S2_72243011 |
| CS283 | *S2_72749782 |
| CS284 | *S2_72872094 |
| CS285 | *S2_73326928 |
| CS286 | *S2_73506652 |
| CS287 | *S2_73725400 |
| CS288 | *S2_73826241 |
| CS289 | *S2_73831779 |
| CS290 | *S2_73831793 |
| CS291 | *S2_73990923 |
| CS292 | *S2_74063027 |
| CS293 | *S2_74334576 |
| CS294 | *S2_75037601 |
| CS295 | *S2_75181638 |
| CS296 | *S2_75181640 |
| CS297 | *S2_75181643 |
| CS298 | *S2_75649255 |
| CS299 | *S2_75845075 |
| CS300 | *S2_75866446 |
| CS301 | *S2_75866469 |
| CS302 | *S2_75866470 |
| CS303 | *S2_77077089 |
| CS304 | *S2_77078186 |
| CS305 | *S2_77078191 |
| CS306 | *S2_77197677 |
| CS307 | *S2_77197710 |
| CS308 | *S2_77334380 |
| CS309 | *S2_5470182 |
| CS310 | *S2_66293861 |
| CS311 | *S2_66293862 |
| CS312 | *S2_392685 |
| CS313 | *S2_466279 |
| CS314 | *S2_510331 |
| CS315 | *S2_1212870 |
| CS316 | *S2_1260309 |
| CS317 | *S2_1571899 |
| CS318 | *S2_1667173 |
| CS319 | *S2_1667179 |
| CS320 | *S2_1693718 |
| CS321 | *S2_1746637 |
| CS322 | *S2_1815652 |
| CS323 | *S2_3686780 |
| CS324 | *S2_3785924 |
| CS325 | *S2_3833615 |
| CS326 | *S2_3856530 |
| CS327 | *S2_4167820 |
| CS328 | *S2_4167829 |
| CS329 | *S2_4167830 |
| CS330 | *S2_4373406 |
| CS331 | *S2_4801136 |
| CS332 | *S2_4825970 |
| CS333 | *S2_5671843 |
| CS334 | *S2_5782821 |
| CS335 | *S2_5800619 |
| CS336 | *S2_5800815 |
| CS337 | *S2_5805982 |
| CS338 | *S2_5887822 |
| CS339 | *S2_5999738 |
| CS340 | *S2_6001436 |
| CS341 | *S2_6143474 |
| CS342 | *S2_6218245 |
| CS343 | *S2_6249850 |
| CS344 | *S2_6264711 |
| CS345 | *S2_6264725 |
| CS346 | *S2_6498084 |
| CS347 | *S2_6700047 |
| CS348 | *S2_6700074 |
| CS349 | *S2_6833609 |
| CS350 | *S2_7448421 |
| CS351 | *S2_7470876 |
| CS352 | *S2_7714990 |
| CS353 | *S2_7715826 |
| CS354 | *S2_7850723 |
| CS355 | *S2_7999987 |
| CS356 | *S2_8369816 |
| CS357 | *S2_8478719 |
| CS358 | *S2_8478918 |
| CS359 | *S2_8486963 |
| CS360 | *S2_9179281 |
| CS361 | *S2_9524789 |
| CS362 | *S2_9557561 |
| CS363 | *S2_9631076 |
| CS364 | *S2_9700948 |
| CS365 | *S2_9997591 |
| CS366 | *S2_10015507 |
| CS367 | *S2_10015540 |
| CS368 | *S2_10026391 |
| CS369 | *S2_10067907 |
| CS370 | *S2_10090626 |
| CS371 | *S2_10343959 |
| CS372 | *S2_10343993 |
| CS373 | *S2_10377097 |
| CS374 | *S2_11147386 |
| CS375 | *S2_11450735 |
| CS376 | *S2_11590683 |
| CS377 | *S2_11885629 |
| CS378 | *S2_12061851 |
| CS379 | *S2_16900803 |
| CS380 | *S2_16900828 |
| CS381 | *S2_18030604 |
| CS382 | *S2_18030873 |
| CS383 | *S2_19764839 |
| CS384 | *S2_19764845 |
| CS385 | *S2_20093770 |
| CS386 | *S2_20989007 |
| CS387 | *S2_21173914 |
| CS388 | *S2_21534333 |
| CS389 | *S2_23019105 |
| CS390 | *S2_40864410 |
| CS391 | *S2_45001145 |
| CS392 | *S2_49474388 |
| CS393 | *S2_55171582 |
| CS394 | *S2_55514061 |
| CS395 | *S2_55562544 |
| CS396 | *S2_56444780 |
| CS397 | *S2_56964445 |
| CS398 | *S2_57017751 |
| CS399 | *S2_57265206 |
| CS400 | *S2_57452527 |
| CS401 | *S2_57452558 |
| CS402 | *S2_57605240 |
| CS403 | *S2_58847311 |
| CS404 | *S2_58867199 |
| CS405 | *S2_59234295 |
| CS406 | *S2_59326715 |
| CS407 | *S2_59326734 |
| CS408 | *S2_59543022 |
| CS409 | *S2_59821866 |
| CS410 | *S2_59821913 |
| CS411 | *S2_60978899 |
| CS412 | *S2_60999538 |
| CS413 | *S2_61098323 |
| CS414 | *S2_61166689 |
| CS415 | *S2_61713569 |
| CS416 | *S2_61811259 |
| CS417 | *S2_62070390 |
| CS418 | *S2_62070392 |
| CS419 | *S2_62070420 |
| CS420 | *S2_62122787 |
| CS421 | *S2_62145285 |
| CS422 | *S2_62148590 |
| CS423 | *S2_62148620 |
| CS424 | *S2_62373925 |
| CS425 | *S2_62402074 |
| CS426 | *S2_63451878 |
| CS427 | *S2_63940992 |
| CS428 | *S2_63993853 |
| CS429 | *S2_64285736 |
| CS430 | *S2_65188596 |
| CS431 | *S2_65258452 |
| CS432 | *S2_66685442 |
| CS433 | *S2_66713725 |
| CS434 | *S2_67011660 |
| CS435 | *S2_67747233 |
| CS436 | *S2_69271774 |
| CS437 | *S2_69459372 |
| CS438 | *S2_69982421 |
| CS439 | *S2_70067668 |
| CS440 | *S2_70067673 |
| CS441 | *S2_70067678 |
| CS442 | *S2_70154736 |
| CS443 | *S2_70238682 |
| CS444 | *S2_70238715 |
| CS445 | *S2_70261820 |
| CS446 | *S2_70262311 |
| CS447 | *S2_70262358 |
| CS448 | *S2_70333287 |
| CS449 | *S2_70390272 |
| CS450 | *S2_70855137 |
| CS451 | *S2_70855174 |
| CS452 | *S2_71063601 |
| CS453 | *S2_71208861 |
| CS454 | *S2_71238475 |
| CS455 | *S2_71630198 |
| CS456 | *S2_72243192 |
| CS457 | *S2_72720531 |
| CS458 | *S2_72752460 |
| CS459 | *S2_72752485 |
| CS460 | *S2_72764322 |
| CS461 | *S2_72823836 |
| CS462 | *S2_72858988 |
| CS463 | *S2_73721733 |
| CS464 | *S2_73721748 |
| CS465 | *S2_73725414 |
| CS466 | *S2_73739672 |
| CS467 | *S2_73762056 |
| CS468 | *S2_73826238 |
| CS469 | *S2_74019631 |
| CS470 | *S2_74944296 |
| CS471 | *S2_75181632 |
| CS472 | *S2_75517284 |
| CS473 | *S2_75517295 |
| CS474 | *S2_75603535 |
| CS475 | *S2_75757864 |
| CS476 | *S2_77077084 |
| CS477 | *S2_77078189 |
| CS478 | *S2_77078190 |
| CS479 | *S2_59710710 |
| CS480 | *S2_66293845 |
| CS481 | *S2_1412219 |
| CS482 | *S2_1674995 |
| CS483 | *S2_1923802 |
| CS484 | *S2_3918972 |
| CS485 | *S2_3967098 |
| CS486 | *S2_4167828 |
| CS487 | *S2_4167833 |
| CS488 | *S2_4284024 |
| CS489 | *S2_4594404 |
| CS490 | *S2_4594410 |
| CS491 | *S2_4916883 |
| CS492 | *S2_5135900 |
| CS493 | *S2_5504646 |
| CS494 | *S2_5633869 |
| CS495 | *S2_5800791 |
| CS496 | *S2_5814325 |
| CS497 | *S2_5838028 |
| CS498 | *S2_5859419 |
| CS499 | *S2_6145492 |
| CS500 | *S2_6235435 |
| CS501 | *S2_6246374 |
| CS502 | *S2_6264731 |
| CS503 | *S2_6284847 |
| CS504 | *S2_6667006 |
| CS505 | *S2_6829392 |
| CS506 | *S2_7957333 |
| CS507 | *S2_8114806 |
| CS508 | *S2_8369747 |
| CS509 | *S2_8972416 |
| CS510 | *S2_9162937 |
| CS511 | *S2_9371124 |
| CS512 | *S2_9805658 |
| CS513 | *S2_10026382 |
| CS514 | *S2_10343995 |
| CS515 | *S2_10379609 |
| CS516 | *S2_11129623 |
| CS517 | *S2_11450701 |
| CS518 | *S2_11707418 |
| CS519 | *S2_11835603 |
| CS520 | *S2_11982079 |
| CS521 | *S2_12564243 |
| CS522 | *S2_13564145 |
| CS523 | *S2_16455887 |
| CS524 | *S2_16463990 |
| CS525 | *S2_19728378 |
| CS526 | *S2_19764805 |
| CS527 | *S2_20093692 |
| CS528 | *S2_23366191 |
| CS529 | *S2_32232452 |
| CS530 | *S2_34439412 |
| CS531 | *S2_35162857 |
| CS532 | *S2_35162858 |
| CS533 | *S2_40329527 |
| CS534 | *S2_43556148 |
| CS535 | *S2_43818827 |
| CS536 | *S2_45001144 |
| CS537 | *S2_46774729 |
| CS538 | *S2_46801389 |
| CS539 | *S2_55170686 |
| CS540 | *S2_56198757 |
| CS541 | *S2_56198761 |
| CS542 | *S2_56198765 |
| CS543 | *S2_56702905 |
| CS544 | *S2_57645574 |
| CS545 | *S2_58233366 |
| CS546 | *S2_58609382 |
| CS547 | *S2_59377248 |
| CS548 | *S2_59912676 |
| CS549 | *S2_59949434 |
| CS550 | *S2_60542829 |
| CS551 | *S2_60791902 |
| CS552 | *S2_60978888 |
| CS553 | *S2_60978890 |
| CS554 | *S2_60978914 |
| CS555 | *S2_60978919 |
| CS556 | *S2_61098320 |
| CS557 | *S2_61134800 |
| CS558 | *S2_61614559 |
| CS559 | *S2_61616289 |
| CS560 | *S2_62040797 |
| CS561 | *S2_62126063 |
| CS562 | *S2_63179991 |
| CS563 | *S2_63940987 |
| CS564 | *S2_63987612 |
| CS565 | *S2_63993852 |
| CS566 | *S2_64225283 |
| CS567 | *S2_64272842 |
| CS568 | *S2_64285727 |
| CS569 | *S2_66923167 |
| CS570 | *S2_67049363 |
| CS571 | *S2_67747168 |
| CS572 | *S2_69441248 |
| CS573 | *S2_69459059 |
| CS574 | *S2_70002353 |
| CS575 | *S2_70005462 |
| CS576 | *S2_70262351 |
| CS577 | *S2_70262395 |
| CS578 | *S2_70512725 |
| CS579 | *S2_70531801 |
| CS580 | *S2_70533358 |
| CS581 | *S2_70720470 |
| CS582 | *S2_70728051 |
| CS583 | *S2_70748404 |
| CS584 | *S2_70749301 |
| CS585 | *S2_72396504 |
| CS586 | *S2_73007471 |
| CS587 | *S2_73767772 |
| CS588 | *S2_73826243 |
| CS589 | *S2_73870486 |
| CS590 | *S2_74052128 |
| CS591 | *S2_74116973 |
| CS592 | *S2_74116991 |
| CS593 | *S2_74334575 |
| CS594 | *S2_75181639 |
| CS595 | *S2_75181642 |
| CS596 | *S2_75181647 |
| CS597 | *S2_75845126 |
| CS598 | *S2_75866453 |
| CS599 | *S2_75866456 |
| CS600 | *S2_75866459 |
| CS601 | *S2_75866460 |
| CS602 | *S2_75866464 |
| CS603 | *S2_75866468 |
| CS604 | *S2_75882930 |
| CS605 | *S2_75882939 |
| CS606 | *S2_76939617 |
| CS607 | *S2_77077086 |
| CS608 | *S2_77078193 |
| CS609 | *S2_77078209 |
| CS610 | *S2_77078213 |
| DB08 | *S3_516244 |
| DB09 | *S3_557476 |
| DB10 | *S3_891594 |
| DB11 | *S3_2165171 |
| DB12 | *S3_2815263 |
| DB13 | *S3_3345854 |
| DB14 | *S3_3955251 |
| DB15 | *S3_3987567 |
| DB16 | *S3_4681218 |
| DB17 | *S3_4683069 |
| DB18 | *S3_4765819 |
| DB19 | *S3_5139389 |
| DB20 | *S3_5702184 |
| DB21 | *S3_5702216 |
| DB22 | *S3_5702217 |
| DB23 | *S3_6014340 |
| DB24 | *S3_6017885 |
| DB25 | *S3_6018578 |
| DB26 | *S3_6187325 |
| DB27 | *S3_6801081 |
| DB28 | *S3_7062579 |
| DB29 | *S3_7062624 |
| DB30 | *S3_7131825 |
| DB31 | *S3_7206433 |
| DB32 | *S3_7801244 |
| DB33 | *S3_8653666 |
| DB34 | *S3_8728713 |
| DB35 | *S3_8954990 |
| DB36 | *S3_9116999 |
| DB37 | *S3_9119710 |
| DB38 | *S3_9119740 |
| DB39 | *S3_9119746 |
| DB40 | *S3_9123261 |
| DB41 | *S3_9123263 |
| DB42 | *S3_9123271 |
| DB43 | *S3_9740551 |
| DB44 | *S3_9763054 |
| DB45 | *S3_10502945 |
| DB46 | *S3_10502951 |
| DB47 | *S3_10563818 |
| DB48 | *S3_10986581 |
| DB49 | *S3_11466679 |
| DB50 | *S3_11466685 |
| DB51 | *S3_12231291 |
| DB52 | *S3_12231297 |
| DB53 | *S3_12231299 |
| DB54 | *S3_12231305 |
| DB55 | *S3_12262961 |
| DB56 | *S3_12379541 |
| DB57 | *S3_12520301 |
| DB58 | *S3_12520313 |
| DB59 | *S3_12520316 |
| DB60 | *S3_13154514 |
| DB61 | *S3_13458369 |
| DB62 | *S3_13463216 |
| DB63 | *S3_13463219 |
| DB64 | *S3_13463221 |
| DB65 | *S3_13463227 |
| DB66 | *S3_13463246 |
| DB67 | *S3_13463260 |
| DB68 | *S3_13463262 |
| DB69 | *S3_13463265 |
| DB70 | *S3_13463271 |
| DB71 | *S3_14262206 |
| DB72 | *S3_15579598 |
| DB73 | *S3_15645911 |
| DB74 | *S3_15715342 |
| DB75 | *S3_15724438 |
| DB76 | *S3_15812919 |
| DB77 | *S3_15920615 |
| DB78 | *S3_16173160 |
| DB79 | *S3_16229514 |
| DB80 | *S3_18352290 |
| DB81 | *S3_18352292 |
| DB82 | *S3_18352293 |
| DB83 | *S3_18352295 |
| DB84 | *S3_19100448 |
| DB85 | *S3_19280276 |
| DB86 | *S3_19280298 |
| DB87 | *S3_19280315 |
| DB88 | *S3_19280316 |
| DB89 | *S3_19407546 |
| DB90 | *S3_20552251 |
| DB91 | *S3_20785119 |
| DB92 | *S3_20880294 |
| DB93 | *S3_22440721 |
| DB94 | *S3_24730341 |
| DB95 | *S3_25183685 |
| DB96 | *S3_26772615 |
| DB97 | *S3_26772639 |
| DB98 | *S3_27778758 |
| DB99 | *S3_27986494 |
| DB100 | *S3_31873757 |
| DB101 | *S3_36343511 |
| DB102 | *S3_37574773 |
| DB103 | *S3_38814663 |
| DB104 | *S3_41552302 |
| DB105 | *S3_41790750 |
| DB106 | *S3_47371504 |
| DB107 | *S3_47738844 |
| DB108 | *S3_48874878 |
| DB109 | *S3_48952842 |
| DB110 | *S3_48953623 |
| DB111 | *S3_48953626 |
| DB112 | *S3_50449563 |
| DB113 | *S3_50449599 |
| DB114 | *S3_51105993 |
| DB115 | *S3_51306550 |
| DB116 | *S3_51453464 |
| DB117 | *S3_51455148 |
| DB118 | *S3_51534710 |
| DB119 | *S3_51619234 |
| DB120 | *S3_52053582 |
| DB121 | *S3_52085525 |
| DB122 | *S3_52410769 |
| DB123 | *S3_53329989 |
| DB124 | *S3_53393694 |
| DB125 | *S3_54149512 |
| DB126 | *S3_55177710 |
| DB127 | *S3_56499146 |
| DB128 | *S3_56499166 |
| DB129 | *S3_56969534 |
| DB130 | *S3_56969810 |
| DB131 | *S3_57167532 |
| DB132 | *S3_57167535 |
| DB133 | *S3_57222701 |
| DB134 | *S3_57331356 |
| DB135 | *S3_57331359 |
| DB136 | *S3_57352322 |
| DB137 | *S3_57860807 |
| DB138 | *S3_57932087 |
| DB139 | *S3_58438046 |
| DB140 | *S3_58456357 |
| DB141 | *S3_58456386 |
| DB142 | *S3_58662188 |
| DB143 | *S3_58907644 |
| DB144 | *S3_59001460 |
| DB145 | *S3_59001474 |
| DB146 | *S3_59124533 |
| DB147 | *S3_59242134 |
| DB148 | *S3_59242393 |
| DB149 | *S3_59300489 |
| DB150 | *S3_59396594 |
| DB151 | *S3_59494271 |
| DB152 | *S3_59494272 |
| DB153 | *S3_59714295 |
| DB154 | *S3_59714302 |
| DB155 | *S3_59714304 |
| DB156 | *S3_59714308 |
| DB157 | *S3_59714309 |
| DB158 | *S3_64319947 |
| DB159 | *S3_64702081 |
| DB160 | *S3_64856857 |
| DB161 | *S3_64927820 |
| DB162 | *S3_64927832 |
| DB163 | *S3_64927837 |
| DB164 | *S3_66091222 |
| DB165 | *S3_68101935 |
| DB166 | *S3_69024262 |
| DB167 | *S3_69323461 |
| DB168 | *S3_69614572 |
| DB169 | *S3_69642374 |
| DB170 | *S3_69666669 |
| DB171 | *S3_69666699 |
| DB172 | *S3_69859889 |
| DB173 | *S3_69884455 |
| DB174 | *S3_69973218 |
| DB175 | *S3_69976835 |
| DB176 | *S3_70192250 |
| DB177 | *S3_70333661 |
| DB178 | *S3_70333663 |
| DB179 | *S3_70333664 |
| DB180 | *S3_70502927 |
| DB181 | *S3_71762371 |
| DB182 | *S3_71762514 |
| DB183 | *S3_72330058 |
| DB184 | *S3_72460203 |
| DB185 | *S3_72681754 |
| DB186 | *S3_73204436 |
| DB187 | *S3_73281474 |
| DB188 | *S3_73539778 |
| DB189 | *S3_73539781 |
| DB190 | *S3_73539790 |
| DB191 | *S3_73750896 |
| DB192 | *S3_74365522 |
| DB193 | *S3_74393681 |
| DB194 | *S3_14395403 |
| DB195 | *S3_557475 |
| DB196 | *S3_613062 |
| DB197 | *S3_863589 |
| DB198 | *S3_1223361 |
| DB199 | *S3_2165158 |
| DB200 | *S3_2165172 |
| DB201 | *S3_2165207 |
| DB202 | *S3_2536860 |
| DB203 | *S3_2556101 |
| DB204 | *S3_2815282 |
| DB205 | *S3_2991290 |
| DB206 | *S3_3009145 |
| DB207 | *S3_3345863 |
| DB208 | *S3_3752434 |
| DB209 | *S3_4682946 |
| DB210 | *S3_4741130 |
| DB211 | *S3_4804702 |
| DB212 | *S3_5044826 |
| DB213 | *S3_5135445 |
| DB214 | *S3_5586620 |
| DB215 | *S3_5945949 |
| DB216 | *S3_6014324 |
| DB217 | *S3_6018579 |
| DB218 | *S3_6018671 |
| DB219 | *S3_6058895 |
| DB220 | *S3_6065395 |
| DB221 | *S3_6161832 |
| DB222 | *S3_6161834 |
| DB223 | *S3_6161900 |
| DB224 | *S3_6344202 |
| DB225 | *S3_6357866 |
| DB226 | *S3_6994182 |
| DB227 | *S3_7062638 |
| DB228 | *S3_7443661 |
| DB229 | *S3_7451045 |
| DB230 | *S3_8008571 |
| DB231 | *S3_8194187 |
| DB232 | *S3_8489475 |
| DB233 | *S3_8626395 |
| DB234 | *S3_8664089 |
| DB235 | *S3_8751429 |
| DB236 | *S3_8974786 |
| DB237 | *S3_9123260 |
| DB238 | *S3_9123270 |
| DB239 | *S3_9740552 |
| DB240 | *S3_10283093 |
| DB241 | *S3_10563844 |
| DB242 | *S3_10986574 |
| DB243 | *S3_10986576 |
| DB244 | *S3_10986580 |
| DB245 | *S3_11466682 |
| DB246 | *S3_11542201 |
| DB247 | *S3_11938329 |
| DB248 | *S3_12124205 |
| DB249 | *S3_12231303 |
| DB250 | *S3_12231308 |
| DB251 | *S3_12301684 |
| DB252 | *S3_12356222 |
| DB253 | *S3_12505833 |
| DB254 | *S3_13463225 |
| DB255 | *S3_13463231 |
| DB256 | *S3_13463242 |
| DB257 | *S3_13463259 |
| DB258 | *S3_13682950 |
| DB259 | *S3_14997855 |
| DB260 | *S3_15147045 |
| DB261 | *S3_15488208 |
| DB262 | *S3_15604912 |
| DB263 | *S3_15649822 |
| DB264 | *S3_16843376 |
| DB265 | *S3_16857040 |
| DB266 | *S3_17017175 |
| DB267 | *S3_18693024 |
| DB268 | *S3_19280272 |
| DB269 | *S3_19614626 |
| DB270 | *S3_19741880 |
| DB271 | *S3_20288483 |
| DB272 | *S3_20423783 |
| DB273 | *S3_20573203 |
| DB274 | *S3_27358015 |
| DB275 | *S3_27983167 |
| DB276 | *S3_29185613 |
| DB277 | *S3_38260196 |
| DB278 | *S3_38818451 |
| DB279 | *S3_40628248 |
| DB280 | *S3_41552297 |
| DB281 | *S3_42123340 |
| DB282 | *S3_44456348 |
| DB283 | *S3_44563855 |
| DB284 | *S3_47227114 |
| DB285 | *S3_47738831 |
| DB286 | *S3_48952712 |
| DB287 | *S3_48953608 |
| DB288 | *S3_50209836 |
| DB289 | *S3_51039837 |
| DB290 | *S3_51580429 |
| DB291 | *S3_51977927 |
| DB292 | *S3_52465324 |
| DB293 | *S3_53346752 |
| DB294 | *S3_54118105 |
| DB295 | *S3_54247480 |
| DB296 | *S3_55488588 |
| DB297 | *S3_56541259 |
| DB298 | *S3_56551572 |
| DB299 | *S3_57075242 |
| DB300 | *S3_57075248 |
| DB301 | *S3_57222691 |
| DB302 | *S3_57269629 |
| DB303 | *S3_57331353 |
| DB304 | *S3_57505545 |
| DB305 | *S3_58555234 |
| DB306 | *S3_58584633 |
| DB307 | *S3_58662174 |
| DB308 | *S3_59298611 |
| DB309 | *S3_59309680 |
| DB310 | *S3_59396595 |
| DB311 | *S3_59614900 |
| DB312 | *S3_59634050 |
| DB313 | *S3_59642787 |
| DB314 | *S3_59676987 |
| DB315 | *S3_59714296 |
| DB316 | *S3_62604492 |
| DB317 | *S3_63211697 |
| DB318 | *S3_63568350 |
| DB319 | *S3_64327714 |
| DB320 | *S3_64578059 |
| DB321 | *S3_64856835 |
| DB322 | *S3_64969718 |
| DB323 | *S3_65584238 |
| DB324 | *S3_65968600 |
| DB325 | *S3_66108826 |
| DB326 | *S3_66960028 |
| DB327 | *S3_68101937 |
| DB328 | *S3_69024223 |
| DB329 | *S3_69531413 |
| DB330 | *S3_69859888 |
| DB331 | *S3_69867763 |
| DB332 | *S3_69953626 |
| DB333 | *S3_69953627 |
| DB334 | *S3_70141162 |
| DB335 | *S3_70286258 |
| DB336 | *S3_70333646 |
| DB337 | *S3_70362756 |
| DB338 | *S3_70597429 |
| DB339 | *S3_70623361 |
| DB340 | *S3_72152701 |
| DB341 | *S3_72846184 |
| DB342 | *S3_73072536 |
| DB343 | *S3_73223066 |
| DB344 | *S3_73289994 |
| DB345 | *S3_73539788 |
| DB346 | *S3_74017792 |
| DB347 | *S3_74150279 |
| DB348 | *S3_74243005 |
| DB349 | *S3_3943445 |
| DB350 | *S3_8101654 |
| DB351 | *S3_15627259 |
| DB352 | *S3_72121927 |
| DB353 | *S3_557477 |
| DB354 | *S3_612391 |
| DB355 | *S3_612401 |
| DB356 | *S3_629729 |
| DB357 | *S3_942490 |
| DB358 | *S3_977201 |
| DB359 | *S3_2535790 |
| DB360 | *S3_2535806 |
| DB361 | *S3_2815306 |
| DB362 | *S3_3201394 |
| DB363 | *S3_3370781 |
| DB364 | *S3_3677339 |
| DB365 | *S3_3724718 |
| DB366 | *S3_3973987 |
| DB367 | *S3_4648921 |
| DB368 | *S3_4829802 |
| DB369 | *S3_5135478 |
| DB370 | *S3_5276663 |
| DB371 | *S3_5696514 |
| DB372 | *S3_6119117 |
| DB373 | *S3_6771649 |
| DB374 | *S3_6801077 |
| DB375 | *S3_7010497 |
| DB376 | *S3_7026314 |
| DB377 | *S3_7120221 |
| DB378 | *S3_7133839 |
| DB379 | *S3_7146106 |
| DB380 | *S3_7152687 |
| DB381 | *S3_7191324 |
| DB382 | *S3_7504262 |
| DB383 | *S3_7523404 |
| DB384 | *S3_7853545 |
| DB385 | *S3_8008523 |
| DB386 | *S3_8089372 |
| DB387 | *S3_8842438 |
| DB388 | *S3_9116322 |
| DB389 | *S3_9117023 |
| DB390 | *S3_9119843 |
| DB391 | *S3_9123267 |
| DB392 | *S3_9123269 |
| DB393 | *S3_9501228 |
| DB394 | *S3_9501234 |
| DB395 | *S3_9665522 |
| DB396 | *S3_10012147 |
| DB397 | *S3_10324083 |
| DB398 | *S3_10563872 |
| DB399 | *S3_10589201 |
| DB400 | *S3_10986577 |
| DB401 | *S3_11089978 |
| DB402 | *S3_11466672 |
| DB403 | *S3_11466676 |
| DB404 | *S3_11659546 |
| DB405 | *S3_11667131 |
| DB406 | *S3_11783427 |
| DB407 | *S3_11909508 |
| DB408 | *S3_12231292 |
| DB409 | *S3_12231296 |
| DB410 | *S3_12366012 |
| DB411 | *S3_12520263 |
| DB412 | *S3_12520268 |
| DB413 | *S3_13463217 |
| DB414 | *S3_13463218 |
| DB415 | *S3_13463229 |
| DB416 | *S3_13463244 |
| DB417 | *S3_13463245 |
| DB418 | *S3_13463248 |
| DB419 | *S3_13463250 |
| DB420 | *S3_13463258 |
| DB421 | *S3_13463264 |
| DB422 | *S3_13487350 |
| DB423 | *S3_13682948 |
| DB424 | *S3_13682951 |
| DB425 | *S3_13682953 |
| DB426 | *S3_14257069 |
| DB427 | *S3_14305649 |
| DB428 | *S3_14708275 |
| DB429 | *S3_15488253 |
| DB430 | *S3_15645920 |
| DB431 | *S3_15659785 |
| DB432 | *S3_15724307 |
| DB433 | *S3_15804270 |
| DB434 | *S3_15976495 |
| DB435 | *S3_16173185 |
| DB436 | *S3_17163295 |
| DB437 | *S3_17660284 |
| DB438 | *S3_17660296 |
| DB439 | *S3_20086459 |
| DB440 | *S3_22555695 |
| DB441 | *S3_24730368 |
| DB442 | *S3_27760983 |
| DB443 | *S3_29153024 |
| DB444 | *S3_29511382 |
| DB445 | *S3_29738080 |
| DB446 | *S3_31030362 |
| DB447 | *S3_32478032 |
| DB448 | *S3_34977563 |
| DB449 | *S3_37663331 |
| DB450 | *S3_41272644 |
| DB451 | *S3_43861367 |
| DB452 | *S3_44890688 |
| DB453 | *S3_47473546 |
| DB454 | *S3_47738776 |
| DB455 | *S3_47738840 |
| DB456 | *S3_47738849 |
| DB457 | *S3_48952625 |
| DB458 | *S3_48952835 |
| DB459 | *S3_48953605 |
| DB460 | *S3_51085633 |
| DB461 | *S3_51306587 |
| DB462 | *S3_51453462 |
| DB463 | *S3_51977483 |
| DB464 | *S3_52053631 |
| DB465 | *S3_52085484 |
| DB466 | *S3_52241958 |
| DB467 | *S3_53322524 |
| DB468 | *S3_53329998 |
| DB469 | *S3_53345740 |
| DB470 | *S3_53346782 |
| DB471 | *S3_53393724 |
| DB472 | *S3_53921360 |
| DB473 | *S3_54111809 |
| DB474 | *S3_54247521 |
| DB475 | *S3_55200283 |
| DB476 | *S3_55200353 |
| DB477 | *S3_55200363 |
| DB478 | *S3_55285382 |
| DB479 | *S3_55424017 |
| DB480 | *S3_56499135 |
| DB481 | *S3_56550930 |
| DB482 | *S3_56969805 |
| DB483 | *S3_57055347 |
| DB484 | *S3_57220985 |
| DB485 | *S3_57222724 |
| DB486 | *S3_57331351 |
| DB487 | *S3_57331367 |
| DB488 | *S3_57705518 |
| DB489 | *S3_57705579 |
| DB490 | *S3_57958033 |
| DB491 | *S3_58240976 |
| DB492 | *S3_58325049 |
| DB493 | *S3_59269038 |
| DB494 | *S3_59298606 |
| DB495 | *S3_59336852 |
| DB496 | *S3_59396598 |
| DB497 | *S3_59396639 |
| DB498 | *S3_59480046 |
| DB499 | *S3_59494273 |
| DB500 | *S3_59579459 |
| DB501 | *S3_59712076 |
| DB502 | *S3_59714298 |
| DB503 | *S3_59714300 |
| DB504 | *S3_59714301 |
| DB505 | *S3_59714332 |
| DB506 | *S3_60157688 |
| DB507 | *S3_61431503 |
| DB508 | *S3_63208961 |
| DB509 | *S3_63211693 |
| DB510 | *S3_64536606 |
| DB511 | *S3_65636481 |
| DB512 | *S3_68199791 |
| DB513 | *S3_69184667 |
| DB514 | *S3_69399097 |
| DB515 | *S3_69867782 |
| DB516 | *S3_69867783 |
| DB517 | *S3_69942706 |
| DB518 | *S3_70141169 |
| DB519 | *S3_70192466 |
| DB520 | *S3_70266717 |
| DB521 | *S3_70294256 |
| DB522 | *S3_70333654 |
| DB523 | *S3_70597428 |
| DB524 | *S3_71196905 |
| DB525 | *S3_71982948 |
| DB526 | *S3_72151881 |
| DB527 | *S3_72393977 |
| DB528 | *S3_72831534 |
| DB529 | *S3_72850799 |
| DB530 | *S3_73223065 |
| DB531 | *S3_73281446 |
| DB532 | *S3_73298194 |
| DB533 | *S3_73465369 |
| DB534 | *S3_73478157 |
| DB535 | *S3_73537938 |
| DB536 | *S3_73558938 |
| DB537 | *S3_73558951 |
| DB538 | *S3_73558952 |
| DB539 | *S3_74017806 |
| DB540 | *S3_557436 |
| DB541 | *S3_557484 |
| DB542 | *S3_613071 |
| DB543 | *S3_907029 |
| DB544 | *S3_945824 |
| DB545 | *S3_1051444 |
| DB546 | *S3_1318131 |
| DB547 | *S3_2146068 |
| DB548 | *S3_2172034 |
| DB549 | *S3_2221380 |
| DB550 | *S3_2251784 |
| DB551 | *S3_2535807 |
| DB552 | *S3_2897194 |
| DB553 | *S3_3671300 |
| DB554 | *S3_3775574 |
| DB555 | *S3_3973997 |
| DB556 | *S3_4011966 |
| DB557 | *S3_4890102 |
| DB558 | *S3_5020748 |
| DB559 | *S3_5044652 |
| DB560 | *S3_5225645 |
| DB561 | *S3_5479271 |
| DB562 | *S3_5679155 |
| DB563 | *S3_5702186 |
| DB564 | *S3_5776265 |
| DB565 | *S3_6083986 |
| DB566 | *S3_6083987 |
| DB567 | *S3_6084033 |
| DB568 | *S3_6162072 |
| DB569 | *S3_6357879 |
| DB570 | *S3_7526023 |
| DB571 | *S3_7999655 |
| DB572 | *S3_8489473 |
| DB573 | *S3_8751407 |
| DB574 | *S3_8974795 |
| DB575 | *S3_9116299 |
| DB576 | *S3_9425262 |
| DB577 | *S3_9501230 |
| DB578 | *S3_10563812 |
| DB579 | *S3_10861988 |
| DB580 | *S3_10986573 |
| DB581 | *S3_11303226 |
| DB582 | *S3_11466687 |
| DB583 | *S3_12231309 |
| DB584 | *S3_12301713 |
| DB585 | *S3_12379576 |
| DB586 | *S3_12515055 |
| DB587 | *S3_12671293 |
| DB588 | *S3_13152767 |
| DB589 | *S3_13463215 |
| DB590 | *S3_13463252 |
| DB591 | *S3_13463267 |
| DB592 | *S3_13707019 |
| DB593 | *S3_14305303 |
| DB594 | *S3_15659773 |
| DB595 | *S3_15660820 |
| DB596 | *S3_15782622 |
| DB597 | *S3_15934530 |
| DB598 | *S3_15976494 |
| DB599 | *S3_16201783 |
| DB600 | *S3_16843426 |
| DB601 | *S3_17831835 |
| DB602 | *S3_19280308 |
| DB603 | *S3_19407556 |
| DB604 | *S3_20022912 |
| DB605 | *S3_20552164 |
| DB606 | *S3_20573192 |
| DB607 | *S3_21553536 |
| DB608 | *S3_22581155 |
| DB609 | *S3_25444991 |
| DB610 | *S3_28348506 |
| DB611 | *S3_28610320 |
| DB612 | *S3_31337796 |
| DB613 | *S3_34552221 |
| DB614 | *S3_40621301 |
| DB615 | *S3_41419135 |
| DB616 | *S3_46253349 |
| DB617 | *S3_47738885 |
| DB618 | *S3_50914547 |
| DB619 | *S3_51081181 |
| DB620 | *S3_51455145 |
| DB621 | *S3_51619221 |
| DB622 | *S3_53346726 |
| DB623 | *S3_54109532 |
| DB624 | *S3_54244841 |
| DB625 | *S3_55200401 |
| DB626 | *S3_55488617 |
| DB627 | *S3_56960671 |
| DB628 | *S3_57333140 |
| DB629 | *S3_57558684 |
| DB630 | *S3_57899858 |
| DB631 | *S3_58240946 |
| DB632 | *S3_58309575 |
| DB633 | *S3_58325079 |
| DB634 | *S3_58430985 |
| DB635 | *S3_58456329 |
| DB636 | *S3_58555325 |
| DB637 | *S3_58584644 |
| DB638 | *S3_58990081 |
| DB639 | *S3_59109211 |
| DB640 | *S3_59300474 |
| DB641 | *S3_59396596 |
| DB642 | *S3_59396597 |
| DB643 | *S3_59480003 |
| DB644 | *S3_59582997 |
| DB645 | *S3_59676983 |
| DB646 | *S3_59676988 |
| DB647 | *S3_59714307 |
| DB648 | *S3_59837831 |
| DB649 | *S3_59963315 |
| DB650 | *S3_61431516 |
| DB651 | *S3_63576449 |
| DB652 | *S3_64223706 |
| DB653 | *S3_64343597 |
| DB654 | *S3_64874831 |
| DB655 | *S3_64969720 |
| DB656 | *S3_65584244 |
| DB657 | *S3_65673028 |
| DB658 | *S3_65945229 |
| DB659 | *S3_66947352 |
| DB660 | *S3_69291030 |
| DB661 | *S3_69304405 |
| DB662 | *S3_69714494 |
| DB663 | *S3_69975227 |
| DB664 | *S3_70286269 |
| DB665 | *S3_70333642 |
| DB666 | *S3_70333644 |
| DB667 | *S3_70333648 |
| DB668 | *S3_70333653 |
| DB669 | *S3_70428135 |
| DB670 | *S3_70502897 |
| DB671 | *S3_70504856 |
| DB672 | *S3_70579755 |
| DB673 | *S3_72846254 |
| DB674 | *S3_73281483 |
| DB675 | *S3_73298188 |
| DB676 | *S3_73539751 |
| DB677 | *S3_73558936 |
| DB678 | *S3_73558945 |
| DB679 | *S3_73558949 |
| DB680 | *S3_73558950 |
| DB681 | *S3_4969227 |
| DB682 | *S3_6154048 |
| BC07 | *S4_1198569 |
| BC08 | *S4_1274623 |
| BC09 | *S4_3058541 |
| BC10 | *S4_3434591 |
| BC11 | *S4_3712100 |
| BC12 | *S4_3732376 |
| BC13 | *S4_3732389 |
| BC14 | *S4_3823650 |
| BC15 | *S4_3943321 |
| BC16 | *S4_4085385 |
| BC17 | *S4_4129822 |
| BC18 | *S4_4251838 |
| BC19 | *S4_4251847 |
| BC20 | *S4_4259530 |
| BC21 | *S4_4265956 |
| BC22 | *S4_4409858 |
| BC23 | *S4_4822831 |
| BC24 | *S4_4922014 |
| BC25 | *S4_5107494 |
| BC26 | *S4_5607465 |
| BC27 | *S4_5782006 |
| BC28 | *S4_5782011 |
| BC29 | *S4_5832683 |
| BC30 | *S4_8071096 |
| BC31 | *S4_8157057 |
| BC32 | *S4_8770449 |
| BC33 | *S4_8925198 |
| BC34 | *S4_8944293 |
| BC35 | *S4_8944300 |
| BC36 | *S4_11288663 |
| BC37 | *S4_11347993 |
| BC38 | *S4_12794690 |
| BC39 | *S4_14266595 |
| BC40 | *S4_14971156 |
| BC41 | *S4_16115566 |
| BC42 | *S4_19811748 |
| BC43 | *S4_20598847 |
| BC44 | *S4_24091625 |
| BC45 | *S4_50562560 |
| BC46 | *S4_52502987 |
| BC47 | *S4_52754314 |
| BC48 | *S4_52754335 |
| BC49 | *S4_52754336 |
| BC50 | *S4_53313027 |
| BC51 | *S4_53316806 |
| BC52 | *S4_53808346 |
| BC53 | *S4_54971217 |
| BC54 | *S4_55074150 |
| BC55 | *S4_56363047 |
| BC56 | *S4_56695037 |
| BC57 | *S4_57322909 |
| BC58 | *S4_61371894 |
| BC59 | *S4_61537423 |
| BC60 | *S4_61564685 |
| BC61 | *S4_61741516 |
| BC62 | *S4_61743538 |
| BC63 | *S4_61973201 |
| BC64 | *S4_62802016 |
| BC65 | *S4_62891969 |
| BC66 | *S4_63389608 |
| BC67 | *S4_65558427 |
| BC68 | *S4_65569720 |
| BC69 | *S4_65600813 |
| BC70 | *S4_65667707 |
| BC71 | *S4_66096589 |
| BC72 | *S4_66462219 |
| BC73 | *S4_66462235 |
| BC74 | *S4_66554368 |
| BC75 | *S4_66557073 |
| BC76 | *S4_66557301 |
| BC77 | *S4_67553055 |
| BC78 | *S4_39644500 |
| BC79 | *S4_1198579 |
| BC80 | *S4_1498940 |
| BC81 | *S4_1498943 |
| BC82 | *S4_2642512 |
| BC83 | *S4_2642516 |
| BC84 | *S4_2649282 |
| BC85 | *S4_3375902 |
| BC86 | *S4_3712069 |
| BC87 | *S4_3823654 |
| BC88 | *S4_3834280 |
| BC89 | *S4_3885030 |
| BC90 | *S4_3927425 |
| BC91 | *S4_4251850 |
| BC92 | *S4_4259547 |
| BC93 | *S4_8819733 |
| BC94 | *S4_8819735 |
| BC95 | *S4_8819736 |
| BC96 | *S4_9437746 |
| BC97 | *S4_9550592 |
| BC98 | *S4_9659169 |
| BC99 | *S4_9709936 |
| BC100 | *S4_9978295 |
| BC101 | *S4_9983831 |
| BC102 | *S4_13113180 |
| BC103 | *S4_13122012 |
| BC104 | *S4_13602308 |
| BC105 | *S4_14197589 |
| BC106 | *S4_14279355 |
| BC107 | *S4_14971155 |
| BC108 | *S4_27680120 |
| BC109 | *S4_43275579 |
| BC110 | *S4_48920238 |
| BC111 | *S4_48920241 |
| BC112 | *S4_49240606 |
| BC113 | *S4_49987809 |
| BC114 | *S4_51691331 |
| BC115 | *S4_51712350 |
| BC116 | *S4_51718132 |
| BC117 | *S4_52754317 |
| BC118 | *S4_52754338 |
| BC119 | *S4_53132977 |
| BC120 | *S4_53135624 |
| BC121 | *S4_53316842 |
| BC122 | *S4_53331324 |
| BC123 | *S4_53334441 |
| BC124 | *S4_54196323 |
| BC125 | *S4_54825006 |
| BC126 | *S4_54916207 |
| BC127 | *S4_55775139 |
| BC128 | *S4_56379600 |
| BC129 | *S4_56695033 |
| BC130 | *S4_56769806 |
| BC131 | *S4_56838966 |
| BC132 | *S4_57256561 |
| BC133 | *S4_61322395 |
| BC134 | *S4_61556209 |
| BC135 | *S4_61566681 |
| BC136 | *S4_61744257 |
| BC137 | *S4_61798713 |
| BC138 | *S4_62826776 |
| BC139 | *S4_62892010 |
| BC140 | *S4_65678951 |
| BC141 | *S4_65678966 |
| BC142 | *S4_65678972 |
| BC143 | *S4_65965090 |
| BC144 | *S4_67248001 |
| BC145 | *S4_55463387 |
| BC146 | *S4_409492 |
| BC147 | *S4_409552 |
| BC148 | *S4_1198586 |
| BC149 | *S4_1498945 |
| BC150 | *S4_3058522 |
| BC151 | *S4_3058526 |
| BC152 | *S4_3434590 |
| BC153 | *S4_3434637 |
| BC154 | *S4_3718702 |
| BC155 | *S4_3732377 |
| BC156 | *S4_3747329 |
| BC157 | *S4_4085365 |
| BC158 | *S4_4251835 |
| BC159 | *S4_4251837 |
| BC160 | *S4_4251842 |
| BC161 | *S4_4251843 |
| BC162 | *S4_4261396 |
| BC163 | *S4_4480455 |
| BC164 | *S4_4822843 |
| BC165 | *S4_4849691 |
| BC166 | *S4_5385499 |
| BC167 | *S4_5512960 |
| BC168 | *S4_5782040 |
| BC169 | *S4_6112324 |
| BC170 | *S4_6112329 |
| BC171 | *S4_6881476 |
| BC172 | *S4_8416929 |
| BC173 | *S4_8925148 |
| BC174 | *S4_8925152 |
| BC175 | *S4_8925154 |
| BC176 | *S4_9550586 |
| BC177 | *S4_9709932 |
| BC178 | *S4_9709937 |
| BC179 | *S4_9971894 |
| BC180 | *S4_10701524 |
| BC181 | *S4_11241238 |
| BC182 | *S4_11241291 |
| BC183 | *S4_12452950 |
| BC184 | *S4_13890094 |
| BC185 | *S4_14266580 |
| BC186 | *S4_14971154 |
| BC187 | *S4_14971157 |
| BC188 | *S4_44987774 |
| BC189 | *S4_49786231 |
| BC190 | *S4_49987798 |
| BC191 | *S4_50909758 |
| BC192 | *S4_51718860 |
| BC193 | *S4_52720940 |
| BC194 | *S4_52754313 |
| BC195 | *S4_52754316 |
| BC196 | *S4_52754320 |
| BC197 | *S4_52754327 |
| BC198 | *S4_53135590 |
| BC199 | *S4_53135591 |
| BC200 | *S4_53135620 |
| BC201 | *S4_53313035 |
| BC202 | *S4_53313041 |
| BC203 | *S4_53313050 |
| BC204 | *S4_53316843 |
| BC205 | *S4_53336024 |
| BC206 | *S4_54875797 |
| BC207 | *S4_54974962 |
| BC208 | *S4_54975785 |
| BC209 | *S4_54982672 |
| BC210 | *S4_55855068 |
| BC211 | *S4_56171060 |
| BC212 | *S4_56259508 |
| BC213 | *S4_56296662 |
| BC214 | *S4_56681989 |
| BC215 | *S4_56695031 |
| BC216 | *S4_56695036 |
| BC217 | *S4_56769847 |
| BC218 | *S4_57256573 |
| BC219 | *S4_57322907 |
| BC220 | *S4_57322910 |
| BC221 | *S4_57402896 |
| BC222 | *S4_61556928 |
| BC223 | *S4_61741508 |
| BC224 | *S4_61813239 |
| BC225 | *S4_62285830 |
| BC226 | *S4_62290250 |
| BC227 | *S4_62290299 |
| BC228 | *S4_62751273 |
| BC229 | *S4_64504097 |
| BC230 | *S4_65558425 |
| BC231 | *S4_65558433 |
| BC232 | *S4_65678967 |
| BC233 | *S4_65690579 |
| BC234 | *S4_65964988 |
| BC235 | *S4_66649824 |
| BC236 | *S4_67150780 |
| BC237 | *S4_68023405 |
| BC238 | *S4_55704180 |
| BC239 | *S4_56813032 |
| BC240 | *S4_804748 |
| BC241 | *S4_1498941 |
| BC242 | *S4_1498946 |
| BC243 | *S4_3058520 |
| BC244 | *S4_3058533 |
| BC245 | *S4_3245295 |
| BC246 | *S4_3400981 |
| BC247 | *S4_4028166 |
| BC248 | *S4_4028168 |
| BC249 | *S4_4028177 |
| BC250 | *S4_4085358 |
| BC251 | *S4_4251845 |
| BC252 | *S4_4251848 |
| BC253 | *S4_4259533 |
| BC254 | *S4_4259534 |
| BC255 | *S4_4609756 |
| BC256 | *S4_5678928 |
| BC257 | *S4_5781991 |
| BC258 | *S4_6112328 |
| BC259 | *S4_8152730 |
| BC260 | *S4_8154210 |
| BC261 | *S4_8154217 |
| BC262 | *S4_8780607 |
| BC263 | *S4_8819734 |
| BC264 | *S4_8838605 |
| BC265 | *S4_8925150 |
| BC266 | *S4_8944295 |
| BC267 | *S4_9550561 |
| BC268 | *S4_9550612 |
| BC269 | *S4_10701546 |
| BC270 | *S4_10703017 |
| BC271 | *S4_11288686 |
| BC272 | *S4_11347595 |
| BC273 | *S4_12728697 |
| BC274 | *S4_13113150 |
| BC275 | *S4_13429772 |
| BC276 | *S4_14266554 |
| BC277 | *S4_14279574 |
| BC278 | *S4_15842223 |
| BC279 | *S4_48802271 |
| BC280 | *S4_52754311 |
| BC281 | *S4_52754343 |
| BC282 | *S4_53135603 |
| BC283 | *S4_53313021 |
| BC284 | *S4_54825015 |
| BC285 | *S4_54871067 |
| BC286 | *S4_56027117 |
| BC287 | *S4_56198686 |
| BC288 | *S4_57322908 |
| BC289 | *S4_57322912 |
| BC290 | *S4_61385088 |
| BC291 | *S4_61543952 |
| BC292 | *S4_61659261 |
| BC293 | *S4_61741522 |
| BC294 | *S4_61790393 |
| BC295 | *S4_61854304 |
| BC296 | *S4_62204740 |
| BC297 | *S4_62557603 |
| BC298 | *S4_64504043 |
| BC299 | *S4_64504072 |
| BC300 | *S4_65681108 |
| BC301 | *S4_65965014 |
| BC302 | *S4_66096273 |
| BC303 | *S4_66471267 |
| BC304 | *S4_66471275 |
| BC305 | *S4_68019638 |
| BC306 | *S4_48674786 |
| AD12 | *S5_463057 |
| AD13 | *S5_595322 |
| AD14 | *S5_1629622 |
| AD15 | *S5_1680987 |
| AD16 | *S5_1938042 |
| AD17 | *S5_2101165 |
| AD18 | *S5_3079216 |
| AD19 | *S5_3904698 |
| AD20 | *S5_3904703 |
| AD21 | *S5_3904704 |
| AD22 | *S5_3904705 |
| AD23 | *S5_4326739 |
| AD24 | *S5_4412239 |
| AD25 | *S5_4569760 |
| AD26 | *S5_8909780 |
| AD27 | *S5_10359599 |
| AD28 | *S5_10952110 |
| AD29 | *S5_12935195 |
| AD30 | *S5_13721213 |
| AD31 | *S5_16056336 |
| AD32 | *S5_18314093 |
| AD33 | *S5_18382525 |
| AD34 | *S5_18458772 |
| AD35 | *S5_18458792 |
| AD36 | *S5_19718928 |
| AD37 | *S5_20264531 |
| AD38 | *S5_21666726 |
| AD39 | *S5_22738766 |
| AD40 | *S5_23038032 |
| AD41 | *S5_25503220 |
| AD42 | *S5_26127716 |
| AD43 | *S5_26524342 |
| AD44 | *S5_26775247 |
| AD45 | *S5_29602390 |
| AD46 | *S5_30478062 |
| AD47 | *S5_32362347 |
| AD48 | *S5_33633924 |
| AD49 | *S5_34242476 |
| AD50 | *S5_37041241 |
| AD51 | *S5_37440630 |
| AD52 | *S5_40963461 |
| AD53 | *S5_41089449 |
| AD54 | *S5_41701419 |
| AD55 | *S5_42285785 |
| AD56 | *S5_42661103 |
| AD57 | *S5_44545049 |
| AD58 | *S5_45244752 |
| AD59 | *S5_46661090 |
| AD60 | *S5_47005158 |
| AD61 | *S5_47232964 |
| AD62 | *S5_47502427 |
| AD63 | *S5_47582166 |
| AD64 | *S5_47582175 |
| AD65 | *S5_49932868 |
| AD66 | *S5_49932876 |
| AD67 | *S5_50248842 |
| AD68 | *S5_51587298 |
| AD69 | *S5_51600381 |
| AD70 | *S5_51636205 |
| AD71 | *S5_51639880 |
| AD72 | *S5_51719163 |
| AD73 | *S5_51719165 |
| AD74 | *S5_51719166 |
| AD75 | *S5_52142335 |
| AD76 | *S5_52307511 |
| AD77 | *S5_52470360 |
| AD78 | *S5_52830119 |
| AD79 | *S5_53105079 |
| AD80 | *S5_53192141 |
| AD81 | *S5_53419618 |
| AD82 | *S5_54624950 |
| AD83 | *S5_54892963 |
| AD84 | *S5_55113340 |
| AD85 | *S5_55127814 |
| AD86 | *S5_55136400 |
| AD87 | *S5_55459699 |
| AD88 | *S5_55741119 |
| AD89 | *S5_55741153 |
| AD90 | *S5_57510191 |
| AD91 | *S5_58020160 |
| AD92 | *S5_58737474 |
| AD93 | *S5_58901930 |
| AD94 | *S5_59339811 |
| AD95 | *S5_59341425 |
| AD96 | *S5_59364207 |
| AD97 | *S5_59437748 |
| AD98 | *S5_59551863 |
| AD99 | *S5_59555764 |
| AD100 | *S5_59590085 |
| AD101 | *S5_60194632 |
| AD102 | *S5_60199179 |
| AD103 | *S5_60318350 |
| AD104 | *S5_60343169 |
| AD105 | *S5_60350128 |
| AD106 | *S5_60907955 |
| AD107 | *S5_61208686 |
| AD108 | *S5_61759702 |
| AD109 | *S5_463148 |
| AD110 | *S5_881098 |
| AD111 | *S5_1122798 |
| AD112 | *S5_1122807 |
| AD113 | *S5_1629663 |
| AD114 | *S5_1938078 |
| AD115 | *S5_1938079 |
| AD116 | *S5_2250420 |
| AD117 | *S5_2650854 |
| AD118 | *S5_2651373 |
| AD119 | *S5_2947052 |
| AD120 | *S5_3923383 |
| AD121 | *S5_4569746 |
| AD122 | *S5_4569747 |
| AD123 | *S5_4569756 |
| AD124 | *S5_4569765 |
| AD125 | *S5_4750763 |
| AD126 | *S5_4755032 |
| AD127 | *S5_4770013 |
| AD128 | *S5_4860214 |
| AD129 | *S5_5098134 |
| AD130 | *S5_10502536 |
| AD131 | *S5_10894099 |
| AD132 | *S5_10952104 |
| AD133 | *S5_11324814 |
| AD134 | *S5_11470416 |
| AD135 | *S5_12988240 |
| AD136 | *S5_13001332 |
| AD137 | *S5_13001906 |
| AD138 | *S5_14428620 |
| AD139 | *S5_15781691 |
| AD140 | *S5_15889492 |
| AD141 | *S5_16321400 |
| AD142 | *S5_18458742 |
| AD143 | *S5_18458743 |
| AD144 | *S5_18458782 |
| AD145 | *S5_18608732 |
| AD146 | *S5_19380556 |
| AD147 | *S5_20136307 |
| AD148 | *S5_20361864 |
| AD149 | *S5_20756004 |
| AD150 | *S5_21704629 |
| AD151 | *S5_21906714 |
| AD152 | *S5_22803804 |
| AD153 | *S5_27934545 |
| AD154 | *S5_28401503 |
| AD155 | *S5_29045725 |
| AD156 | *S5_35926604 |
| AD157 | *S5_37452086 |
| AD158 | *S5_42764164 |
| AD159 | *S5_44250216 |
| AD160 | *S5_44545040 |
| AD161 | *S5_45237396 |
| AD162 | *S5_45238075 |
| AD163 | *S5_46576049 |
| AD164 | *S5_48523648 |
| AD165 | *S5_49815056 |
| AD166 | *S5_49860844 |
| AD167 | *S5_49931675 |
| AD168 | *S5_51719134 |
| AD169 | *S5_51719160 |
| AD170 | *S5_51719176 |
| AD171 | *S5_52228189 |
| AD172 | *S5_52943190 |
| AD173 | *S5_52943205 |
| AD174 | *S5_52976513 |
| AD175 | *S5_52980342 |
| AD176 | *S5_53190874 |
| AD177 | *S5_53380348 |
| AD178 | *S5_53419622 |
| AD179 | *S5_53419625 |
| AD180 | *S5_53419637 |
| AD181 | *S5_53614123 |
| AD182 | *S5_53978073 |
| AD183 | *S5_54413461 |
| AD184 | *S5_55113330 |
| AD185 | *S5_55419983 |
| AD186 | *S5_55459838 |
| AD187 | *S5_55539485 |
| AD188 | *S5_55741143 |
| AD189 | *S5_55741150 |
| AD190 | *S5_55741154 |
| AD191 | *S5_55745314 |
| AD192 | *S5_56127356 |
| AD193 | *S5_57099017 |
| AD194 | *S5_57157187 |
| AD195 | *S5_57491964 |
| AD196 | *S5_57609641 |
| AD197 | *S5_58210427 |
| AD198 | *S5_58219042 |
| AD199 | *S5_58413608 |
| AD200 | *S5_58413616 |
| AD201 | *S5_58476682 |
| AD202 | *S5_58603386 |
| AD203 | *S5_58736736 |
| AD204 | *S5_58757100 |
| AD205 | *S5_58930123 |
| AD206 | *S5_58937550 |
| AD207 | *S5_59555776 |
| AD208 | *S5_59626445 |
| AD209 | *S5_60092156 |
| AD210 | *S5_60169845 |
| AD211 | *S5_60343168 |
| AD212 | *S5_60347039 |
| AD213 | *S5_60350129 |
| AD214 | *S5_60358761 |
| AD215 | *S5_60358764 |
| AD216 | *S5_60633765 |
| AD217 | *S5_60715269 |
| AD218 | *S5_60828419 |
| AD219 | *S5_61102661 |
| AD220 | *S5_61208682 |
| AD221 | *S5_61208683 |
| AD222 | *S5_322989 |
| AD223 | *S5_323015 |
| AD224 | *S5_881071 |
| AD225 | *S5_881111 |
| AD226 | *S5_1629720 |
| AD227 | *S5_1938095 |
| AD228 | *S5_1939098 |
| AD229 | *S5_1939104 |
| AD230 | *S5_2013607 |
| AD231 | *S5_2101237 |
| AD232 | *S5_2250419 |
| AD233 | *S5_2250424 |
| AD234 | *S5_2432565 |
| AD235 | *S5_2780030 |
| AD236 | *S5_2796386 |
| AD237 | *S5_3068361 |
| AD238 | *S5_4326710 |
| AD239 | *S5_4810761 |
| AD240 | *S5_9695162 |
| AD241 | *S5_10502480 |
| AD242 | *S5_10952111 |
| AD243 | *S5_11193966 |
| AD244 | *S5_11249169 |
| AD245 | *S5_14411620 |
| AD246 | *S5_16018291 |
| AD247 | *S5_16321367 |
| AD248 | *S5_17202131 |
| AD249 | *S5_17648901 |
| AD250 | *S5_18641230 |
| AD251 | *S5_19646296 |
| AD252 | *S5_20814433 |
| AD253 | *S5_21335182 |
| AD254 | *S5_22333332 |
| AD255 | *S5_23214595 |
| AD256 | *S5_28915654 |
| AD257 | *S5_29045720 |
| AD258 | *S5_30663553 |
| AD259 | *S5_30801025 |
| AD260 | *S5_31132258 |
| AD261 | *S5_31919756 |
| AD262 | *S5_36112623 |
| AD263 | *S5_41215861 |
| AD264 | *S5_41448018 |
| AD265 | *S5_42715473 |
| AD266 | *S5_43017819 |
| AD267 | *S5_43860319 |
| AD268 | *S5_44396289 |
| AD269 | *S5_44396316 |
| AD270 | *S5_45134983 |
| AD271 | *S5_45758238 |
| AD272 | *S5_46244703 |
| AD273 | *S5_46384455 |
| AD274 | *S5_46518988 |
| AD275 | *S5_46519004 |
| AD276 | *S5_46576051 |
| AD277 | *S5_46661083 |
| AD278 | *S5_47232935 |
| AD279 | *S5_48513663 |
| AD280 | *S5_49805073 |
| AD281 | *S5_49805095 |
| AD282 | *S5_49805388 |
| AD283 | *S5_49860845 |
| AD284 | *S5_49932849 |
| AD285 | *S5_51538904 |
| AD286 | *S5_51638479 |
| AD287 | *S5_51719162 |
| AD288 | *S5_51719175 |
| AD289 | *S5_52307510 |
| AD290 | *S5_52307849 |
| AD291 | *S5_52830102 |
| AD292 | *S5_52943211 |
| AD293 | *S5_52980199 |
| AD294 | *S5_52990211 |
| AD295 | *S5_53419620 |
| AD296 | *S5_53419621 |
| AD297 | *S5_53436931 |
| AD298 | *S5_53476647 |
| AD299 | *S5_53891248 |
| AD300 | *S5_54226483 |
| AD301 | *S5_54509627 |
| AD302 | *S5_54821148 |
| AD303 | *S5_55459745 |
| AD304 | *S5_55459844 |
| AD305 | *S5_55459860 |
| AD306 | *S5_55508666 |
| AD307 | *S5_55539516 |
| AD308 | *S5_55655720 |
| AD309 | *S5_55741156 |
| AD310 | *S5_55741158 |
| AD311 | *S5_57100211 |
| AD312 | *S5_57226998 |
| AD313 | *S5_57403266 |
| AD314 | *S5_57491962 |
| AD315 | *S5_57589371 |
| AD316 | *S5_57758642 |
| AD317 | *S5_57775866 |
| AD318 | *S5_58246488 |
| AD319 | *S5_58413606 |
| AD320 | *S5_58736708 |
| AD321 | *S5_58812953 |
| AD322 | *S5_58929102 |
| AD323 | *S5_59009625 |
| AD324 | *S5_59341438 |
| AD325 | *S5_59550073 |
| AD326 | *S5_59551867 |
| AD327 | *S5_59590098 |
| AD328 | *S5_59771618 |
| AD329 | *S5_60005810 |
| AD330 | *S5_60044496 |
| AD331 | *S5_60110479 |
| AD332 | *S5_60343170 |
| AD333 | *S5_60492218 |
| AD334 | *S5_60767028 |
| AD335 | *S5_61050747 |
| AD336 | *S5_61117034 |
| AD337 | *S5_62144731 |
| AD338 | *S5_62168308 |
| AD339 | *S5_62170099 |
| AD340 | *S5_62172237 |
| AD341 | *S5_62175624 |
| AD342 | *S5_60737625 |
| AD343 | *S5_56216 |
| AD344 | *S5_499931 |
| AD345 | *S5_561394 |
| AD346 | *S5_1629615 |
| AD347 | *S5_1629661 |
| AD348 | *S5_2005775 |
| AD349 | *S5_2250423 |
| AD350 | *S5_2250426 |
| AD351 | *S5_2704115 |
| AD352 | *S5_2707518 |
| AD353 | *S5_2939663 |
| AD354 | *S5_2947037 |
| AD355 | *S5_3009449 |
| AD356 | *S5_3404153 |
| AD357 | *S5_4349037 |
| AD358 | *S5_4569741 |
| AD359 | *S5_4569758 |
| AD360 | *S5_4569790 |
| AD361 | *S5_8909761 |
| AD362 | *S5_10036106 |
| AD363 | *S5_10221378 |
| AD364 | *S5_11192584 |
| AD365 | *S5_11193971 |
| AD366 | *S5_12858363 |
| AD367 | *S5_13583946 |
| AD368 | *S5_13721253 |
| AD369 | *S5_14111300 |
| AD370 | *S5_16043885 |
| AD371 | *S5_17151455 |
| AD372 | *S5_17522020 |
| AD373 | *S5_17681360 |
| AD374 | *S5_17747581 |
| AD375 | *S5_17754582 |
| AD376 | *S5_20100073 |
| AD377 | *S5_20264450 |
| AD378 | *S5_20756031 |
| AD379 | *S5_20756055 |
| AD380 | *S5_22590662 |
| AD381 | *S5_27985277 |
| AD382 | *S5_29045719 |
| AD383 | *S5_31926577 |
| AD384 | *S5_31927540 |
| AD385 | *S5_32362348 |
| AD386 | *S5_33633949 |
| AD387 | *S5_41089446 |
| AD388 | *S5_41781092 |
| AD389 | *S5_41848008 |
| AD390 | *S5_42087546 |
| AD391 | *S5_42327420 |
| AD392 | *S5_42327450 |
| AD393 | *S5_42661146 |
| AD394 | *S5_43017806 |
| AD395 | *S5_43017822 |
| AD396 | *S5_44380327 |
| AD397 | *S5_46518972 |
| AD398 | *S5_46575236 |
| AD399 | *S5_46576055 |
| AD400 | *S5_46576056 |
| AD401 | *S5_47614444 |
| AD402 | *S5_47655616 |
| AD403 | *S5_48441692 |
| AD404 | *S5_51587312 |
| AD405 | *S5_51636195 |
| AD406 | *S5_51636212 |
| AD407 | *S5_51636228 |
| AD408 | *S5_51639849 |
| AD409 | *S5_51719137 |
| AD410 | *S5_52242147 |
| AD411 | *S5_52307560 |
| AD412 | *S5_52328702 |
| AD413 | *S5_52565178 |
| AD414 | *S5_52830105 |
| AD415 | *S5_53021039 |
| AD416 | *S5_53190510 |
| AD417 | *S5_53419619 |
| AD418 | *S5_53419659 |
| AD419 | *S5_53476657 |
| AD420 | *S5_53476671 |
| AD421 | *S5_53583329 |
| AD422 | *S5_53998505 |
| AD423 | *S5_54326289 |
| AD424 | *S5_54384715 |
| AD425 | *S5_54611166 |
| AD426 | *S5_54663237 |
| AD427 | *S5_54821153 |
| AD428 | *S5_55741149 |
| AD429 | *S5_55741157 |
| AD430 | *S5_56023594 |
| AD431 | *S5_56096741 |
| AD432 | *S5_56978698 |
| AD433 | *S5_56978731 |
| AD434 | *S5_57099033 |
| AD435 | *S5_57157241 |
| AD436 | *S5_57167110 |
| AD437 | *S5_57403969 |
| AD438 | *S5_57494026 |
| AD439 | *S5_57510187 |
| AD440 | *S5_57775854 |
| AD441 | *S5_58026666 |
| AD442 | *S5_58229918 |
| AD443 | *S5_58413607 |
| AD444 | *S5_58736735 |
| AD445 | *S5_58757103 |
| AD446 | *S5_58757106 |
| AD447 | *S5_59162886 |
| AD448 | *S5_59162888 |
| AD449 | *S5_59229031 |
| AD450 | *S5_59229049 |
| AD451 | *S5_59229073 |
| AD452 | *S5_59284876 |
| AD453 | *S5_59550055 |
| AD454 | *S5_59550064 |
| AD455 | *S5_59550068 |
| AD456 | *S5_59551866 |
| AD457 | *S5_59551876 |
| AD458 | *S5_59590097 |
| AD459 | *S5_59777416 |
| AD460 | *S5_59777458 |
| AD461 | *S5_60005780 |
| AD462 | *S5_60005817 |
| AD463 | *S5_60092198 |
| AD464 | *S5_60194657 |
| AD465 | *S5_60318362 |
| AD466 | *S5_60343166 |
| AD467 | *S5_60633864 |
| AD468 | *S5_60767021 |
| AD469 | *S5_60822928 |
| AD470 | *S5_61139226 |
| AD471 | *S5_61174463 |
| AD472 | *S5_62144991 |
| AD473 | *S5_62162522 |
| AD474 | *S5_62170116 |
| AD475 | *S5_62176154 |
| AD476 | *S5_62184184 |
| EF11 | *S6_452713 |
| EF12 | *S6_772964 |
| EF13 | *S6_1013559 |
| EF14 | *S6_1306601 |
| EF15 | *S6_1553680 |
| EF16 | *S6_1893397 |
| EF17 | *S6_1907468 |
| EF18 | *S6_2975883 |
| EF19 | *S6_3873283 |
| EF20 | *S6_4210195 |
| EF21 | *S6_6574249 |
| EF22 | *S6_9425423 |
| EF23 | *S6_9809019 |
| EF24 | *S6_9809030 |
| EF25 | *S6_10546645 |
| EF26 | *S6_10575052 |
| EF27 | *S6_16857405 |
| EF28 | *S6_19118062 |
| EF29 | *S6_19118063 |
| EF30 | *S6_19118065 |
| EF31 | *S6_19118069 |
| EF32 | *S6_20753723 |
| EF33 | *S6_28724637 |
| EF34 | *S6_29492824 |
| EF35 | *S6_31973273 |
| EF36 | *S6_32185441 |
| EF37 | *S6_35884604 |
| EF38 | *S6_37345488 |
| EF39 | *S6_38155501 |
| EF40 | *S6_38155515 |
| EF41 | *S6_39564155 |
| EF42 | *S6_39727469 |
| EF43 | *S6_40279835 |
| EF44 | *S6_40343840 |
| EF45 | *S6_40763275 |
| EF46 | *S6_41052206 |
| EF47 | *S6_41242050 |
| EF48 | *S6_42049249 |
| EF49 | *S6_42073668 |
| EF50 | *S6_42073684 |
| EF51 | *S6_42073685 |
| EF52 | *S6_42141087 |
| EF53 | *S6_42203658 |
| EF54 | *S6_42213351 |
| EF55 | *S6_42536106 |
| EF56 | *S6_42552290 |
| EF57 | *S6_43670253 |
| EF58 | *S6_43966525 |
| EF59 | *S6_44050463 |
| EF60 | *S6_44532517 |
| EF61 | *S6_44532540 |
| EF62 | *S6_44675314 |
| EF63 | *S6_44740218 |
| EF64 | *S6_44754285 |
| EF65 | *S6_44767076 |
| EF66 | *S6_44767077 |
| EF67 | *S6_44863723 |
| EF68 | *S6_45510053 |
| EF69 | *S6_45907199 |
| EF70 | *S6_45964326 |
| EF71 | *S6_46341205 |
| EF72 | *S6_46473748 |
| EF73 | *S6_47249244 |
| EF74 | *S6_48093867 |
| EF75 | *S6_49454984 |
| EF76 | *S6_49478202 |
| EF77 | *S6_50083373 |
| EF78 | *S6_51228994 |
| EF79 | *S6_52019242 |
| EF80 | *S6_52113675 |
| EF81 | *S6_52813559 |
| EF82 | *S6_53553573 |
| EF83 | *S6_53587329 |
| EF84 | *S6_53590580 |
| EF85 | *S6_53602984 |
| EF86 | *S6_53758993 |
| EF87 | *S6_53850259 |
| EF88 | *S6_53850261 |
| EF89 | *S6_54596615 |
| EF90 | *S6_54874603 |
| EF91 | *S6_55714965 |
| EF92 | *S6_55714982 |
| EF93 | *S6_55753263 |
| EF94 | *S6_55926220 |
| EF95 | *S6_55952113 |
| EF96 | *S6_56770651 |
| EF97 | *S6_56770662 |
| EF98 | *S6_57093891 |
| EF99 | *S6_57159653 |
| EF100 | *S6_57315490 |
| EF101 | *S6_57618671 |
| EF102 | *S6_58252257 |
| EF103 | *S6_58793472 |
| EF104 | *S6_58798195 |
| EF105 | *S6_58839274 |
| EF106 | *S6_59126966 |
| EF107 | *S6_59333998 |
| EF108 | *S6_59640250 |
| EF109 | *S6_59687545 |
| EF110 | *S6_60009817 |
| EF111 | *S6_61123394 |
| EF112 | *S6_61130699 |
| EF113 | *S6_61950135 |
| EF114 | *S6_772940 |
| EF115 | *S6_1512676 |
| EF116 | *S6_1990718 |
| EF117 | *S6_2326188 |
| EF118 | *S6_5993437 |
| EF119 | *S6_6718698 |
| EF120 | *S6_6718722 |
| EF121 | *S6_7640458 |
| EF122 | *S6_9809022 |
| EF123 | *S6_10578793 |
| EF124 | *S6_10984005 |
| EF125 | *S6_11791108 |
| EF126 | *S6_15109353 |
| EF127 | *S6_16411832 |
| EF128 | *S6_18598023 |
| EF129 | *S6_18796480 |
| EF130 | *S6_19118064 |
| EF131 | *S6_19118066 |
| EF132 | *S6_26112877 |
| EF133 | *S6_27945315 |
| EF134 | *S6_30795967 |
| EF135 | *S6_32185455 |
| EF136 | *S6_32185463 |
| EF137 | *S6_32185465 |
| EF138 | *S6_32571266 |
| EF139 | *S6_32737583 |
| EF140 | *S6_33734578 |
| EF141 | *S6_38141886 |
| EF142 | *S6_38155513 |
| EF143 | *S6_41973901 |
| EF144 | *S6_42203715 |
| EF145 | *S6_42536114 |
| EF146 | *S6_42741378 |
| EF147 | *S6_43942081 |
| EF148 | *S6_43966575 |
| EF149 | *S6_44130964 |
| EF150 | *S6_44561177 |
| EF151 | *S6_44598070 |
| EF152 | *S6_44740246 |
| EF153 | *S6_44894209 |
| EF154 | *S6_45897827 |
| EF155 | *S6_45937254 |
| EF156 | *S6_45937256 |
| EF157 | *S6_45939897 |
| EF158 | *S6_46132974 |
| EF159 | *S6_46327218 |
| EF160 | *S6_46408612 |
| EF161 | *S6_46413017 |
| EF162 | *S6_46473737 |
| EF163 | *S6_46473750 |
| EF164 | *S6_46939051 |
| EF165 | *S6_48093853 |
| EF166 | *S6_48093871 |
| EF167 | *S6_48093874 |
| EF168 | *S6_48093877 |
| EF169 | *S6_48412866 |
| EF170 | *S6_48433672 |
| EF171 | *S6_48461973 |
| EF172 | *S6_48623339 |
| EF173 | *S6_48825076 |
| EF174 | *S6_48848856 |
| EF175 | *S6_49518385 |
| EF176 | *S6_49605273 |
| EF177 | *S6_50140549 |
| EF178 | *S6_50140930 |
| EF179 | *S6_50886529 |
| EF180 | *S6_51034079 |
| EF181 | *S6_51091263 |
| EF182 | *S6_52112826 |
| EF183 | *S6_52141708 |
| EF184 | *S6_52637707 |
| EF185 | *S6_52741775 |
| EF186 | *S6_52742381 |
| EF187 | *S6_52813553 |
| EF188 | *S6_52959751 |
| EF189 | *S6_53349007 |
| EF190 | *S6_53419860 |
| EF191 | *S6_53534381 |
| EF192 | *S6_53599301 |
| EF193 | *S6_53668372 |
| EF194 | *S6_53849573 |
| EF195 | *S6_54151194 |
| EF196 | *S6_55714987 |
| EF197 | *S6_55926210 |
| EF198 | *S6_55926217 |
| EF199 | *S6_55926219 |
| EF200 | *S6_56158581 |
| EF201 | *S6_57315506 |
| EF202 | *S6_58381826 |
| EF203 | *S6_58550251 |
| EF204 | *S6_58612252 |
| EF205 | *S6_58923853 |
| EF206 | *S6_60110636 |
| EF207 | *S6_60215347 |
| EF208 | *S6_60219728 |
| EF209 | *S6_60247210 |
| EF210 | *S6_60253958 |
| EF211 | *S6_45290959 |
| EF212 | *S6_59773080 |
| EF213 | *S6_1279163 |
| EF214 | *S6_1930502 |
| EF215 | *S6_2326185 |
| EF216 | *S6_2326202 |
| EF217 | *S6_2689894 |
| EF218 | *S6_3861740 |
| EF219 | *S6_3950853 |
| EF220 | *S6_3969257 |
| EF221 | *S6_7141910 |
| EF222 | *S6_8713098 |
| EF223 | *S6_9425401 |
| EF224 | *S6_9809024 |
| EF225 | *S6_9809043 |
| EF226 | *S6_10275349 |
| EF227 | *S6_10578285 |
| EF228 | *S6_11817228 |
| EF229 | *S6_15109357 |
| EF230 | *S6_16706879 |
| EF231 | *S6_17354192 |
| EF232 | *S6_17646448 |
| EF233 | *S6_18598019 |
| EF234 | *S6_20954189 |
| EF235 | *S6_25518556 |
| EF236 | *S6_26210169 |
| EF237 | *S6_27700910 |
| EF238 | *S6_29465641 |
| EF239 | *S6_30635621 |
| EF240 | *S6_30813078 |
| EF241 | *S6_31031536 |
| EF242 | *S6_31431838 |
| EF243 | *S6_32185437 |
| EF244 | *S6_32501000 |
| EF245 | *S6_33734603 |
| EF246 | *S6_35884557 |
| EF247 | *S6_37949828 |
| EF248 | *S6_38141478 |
| EF249 | *S6_38141895 |
| EF250 | *S6_38823536 |
| EF251 | *S6_38869751 |
| EF252 | *S6_39952632 |
| EF253 | *S6_40440109 |
| EF254 | *S6_40917830 |
| EF255 | *S6_41241055 |
| EF256 | *S6_41973878 |
| EF257 | *S6_42073681 |
| EF258 | *S6_42203544 |
| EF259 | *S6_42535810 |
| EF260 | *S6_42764790 |
| EF261 | *S6_43942078 |
| EF262 | *S6_43966566 |
| EF263 | *S6_44576684 |
| EF264 | *S6_44711070 |
| EF265 | *S6_44786145 |
| EF266 | *S6_44959725 |
| EF267 | *S6_45937255 |
| EF268 | *S6_45937260 |
| EF269 | *S6_46064847 |
| EF270 | *S6_46306149 |
| EF271 | *S6_46306172 |
| EF272 | *S6_46306178 |
| EF273 | *S6_46473733 |
| EF274 | *S6_46473736 |
| EF275 | *S6_46518911 |
| EF276 | *S6_46589196 |
| EF277 | *S6_47241945 |
| EF278 | *S6_48093849 |
| EF279 | *S6_48461948 |
| EF280 | *S6_48701474 |
| EF281 | *S6_48848864 |
| EF282 | *S6_49943798 |
| EF283 | *S6_49946596 |
| EF284 | *S6_49964784 |
| EF285 | *S6_49973827 |
| EF286 | *S6_50089063 |
| EF287 | *S6_50140940 |
| EF288 | *S6_50140944 |
| EF289 | *S6_50140946 |
| EF290 | *S6_51091294 |
| EF291 | *S6_51228955 |
| EF292 | *S6_51228959 |
| EF293 | *S6_51249704 |
| EF294 | *S6_52813555 |
| EF295 | *S6_52813557 |
| EF296 | *S6_52813562 |
| EF297 | *S6_52937937 |
| EF298 | *S6_52994278 |
| EF299 | *S6_53246068 |
| EF300 | *S6_53385710 |
| EF301 | *S6_53385712 |
| EF302 | *S6_53599335 |
| EF303 | *S6_53849566 |
| EF304 | *S6_54874580 |
| EF305 | *S6_54874587 |
| EF306 | *S6_54874600 |
| EF307 | *S6_54874601 |
| EF308 | *S6_55407099 |
| EF309 | *S6_55714978 |
| EF310 | *S6_55714990 |
| EF311 | *S6_55714993 |
| EF312 | *S6_55926222 |
| EF313 | *S6_55928441 |
| EF314 | *S6_56770560 |
| EF315 | *S6_57121609 |
| EF316 | *S6_57314419 |
| EF317 | *S6_57315471 |
| EF318 | *S6_57315473 |
| EF319 | *S6_57315474 |
| EF320 | *S6_57602781 |
| EF321 | *S6_57618674 |
| EF322 | *S6_58247716 |
| EF323 | *S6_58739030 |
| EF324 | *S6_58790832 |
| EF325 | *S6_58839242 |
| EF326 | *S6_59126967 |
| EF327 | *S6_59197458 |
| EF328 | *S6_59229224 |
| EF329 | *S6_59256611 |
| EF330 | *S6_59256615 |
| EF331 | *S6_59295915 |
| EF332 | *S6_59600605 |
| EF333 | *S6_60103937 |
| EF334 | *S6_60247235 |
| EF335 | *S6_60254355 |
| EF336 | *S6_60948282 |
| EF337 | *S6_61049913 |
| EF338 | *S6_358917 |
| EF339 | *S6_1279361 |
| EF340 | *S6_1990734 |
| EF341 | *S6_2689893 |
| EF342 | *S6_3242285 |
| EF343 | *S6_4662279 |
| EF344 | *S6_6574228 |
| EF345 | *S6_7178871 |
| EF346 | *S6_9809015 |
| EF347 | *S6_10275362 |
| EF348 | *S6_10276876 |
| EF349 | *S6_10553831 |
| EF350 | *S6_15101937 |
| EF351 | *S6_16379801 |
| EF352 | *S6_18036445 |
| EF353 | *S6_18892151 |
| EF354 | *S6_19784859 |
| EF355 | *S6_27945269 |
| EF356 | *S6_29492844 |
| EF357 | *S6_31798285 |
| EF358 | *S6_33729154 |
| EF359 | *S6_35877146 |
| EF360 | *S6_38141491 |
| EF361 | *S6_38155502 |
| EF362 | *S6_39652413 |
| EF363 | *S6_39652557 |
| EF364 | *S6_39952671 |
| EF365 | *S6_40763293 |
| EF366 | *S6_42073682 |
| EF367 | *S6_42508250 |
| EF368 | *S6_42535851 |
| EF369 | *S6_42736362 |
| EF370 | *S6_42741535 |
| EF371 | *S6_43223624 |
| EF372 | *S6_43942051 |
| EF373 | *S6_44130948 |
| EF374 | *S6_44553810 |
| EF375 | *S6_44676810 |
| EF376 | *S6_45464314 |
| EF377 | *S6_45897834 |
| EF378 | *S6_45909647 |
| EF379 | *S6_45939957 |
| EF380 | *S6_45964332 |
| EF381 | *S6_46327221 |
| EF382 | *S6_46413016 |
| EF383 | *S6_46473298 |
| EF384 | *S6_46597330 |
| EF385 | *S6_48028488 |
| EF386 | *S6_48461984 |
| EF387 | *S6_48684570 |
| EF388 | *S6_48684573 |
| EF389 | *S6_48685677 |
| EF390 | *S6_48717157 |
| EF391 | *S6_49822968 |
| EF392 | *S6_49871830 |
| EF393 | *S6_50089079 |
| EF394 | *S6_51004379 |
| EF395 | *S6_51034057 |
| EF396 | *S6_51074599 |
| EF397 | *S6_51091295 |
| EF398 | *S6_51228960 |
| EF399 | *S6_51228985 |
| EF400 | *S6_52019244 |
| EF401 | *S6_52065463 |
| EF402 | *S6_52807467 |
| EF403 | *S6_52813554 |
| EF404 | *S6_52813602 |
| EF405 | *S6_52937916 |
| EF406 | *S6_53166651 |
| EF407 | *S6_53349014 |
| EF408 | *S6_53382605 |
| EF409 | *S6_53385781 |
| EF410 | *S6_53599327 |
| EF411 | *S6_53848121 |
| EF412 | *S6_54117301 |
| EF413 | *S6_54117807 |
| EF414 | *S6_55427456 |
| EF415 | *S6_55496931 |
| EF416 | *S6_55714963 |
| EF417 | *S6_55714981 |
| EF418 | *S6_55714988 |
| EF419 | *S6_55926216 |
| EF420 | *S6_55926221 |
| EF421 | *S6_56981792 |
| EF422 | *S6_57004935 |
| EF423 | *S6_57159671 |
| EF424 | *S6_57621779 |
| EF425 | *S6_57800381 |
| EF426 | *S6_58498695 |
| EF427 | *S6_58581422 |
| EF428 | *S6_58783753 |
| EF429 | *S6_59294981 |
| EF430 | *S6_59303254 |
| EF431 | *S6_59600614 |
| EF432 | *S6_59619131 |
| EF433 | *S6_60020084 |
| EF434 | *S6_60220115 |
| EF435 | *S6_60247182 |
| EF436 | *S6_60535458 |
| EF437 | *S6_60936931 |
| EF438 | *S6_61055793 |
| EF439 | *S6_61253235 |
| EF440 | *S6_61950098 |
| EF441 | *S6_39652554 |
| GH12 | *S7_129398 |
| GH13 | *S7_386947 |
| GH14 | *S7_854471 |
| GH15 | *S7_913871 |
| GH16 | *S7_966352 |
| GH17 | *S7_1630207 |
| GH18 | *S7_3785207 |
| GH19 | *S7_3867720 |
| GH20 | *S7_3953213 |
| GH21 | *S7_7242359 |
| GH22 | *S7_7467485 |
| GH23 | *S7_7467487 |
| GH24 | *S7_8637373 |
| GH25 | *S7_8853160 |
| GH26 | *S7_9969777 |
| GH27 | *S7_9969816 |
| GH28 | *S7_12355692 |
| GH29 | *S7_17020795 |
| GH30 | *S7_21666644 |
| GH31 | *S7_22870722 |
| GH32 | *S7_35989356 |
| GH33 | *S7_38080235 |
| GH34 | *S7_38103509 |
| GH35 | *S7_38361575 |
| GH36 | *S7_38388388 |
| GH37 | *S7_39350297 |
| GH38 | *S7_39992212 |
| GH39 | *S7_42622209 |
| GH40 | *S7_44518318 |
| GH41 | *S7_44518335 |
| GH42 | *S7_48071204 |
| GH43 | *S7_48359636 |
| GH44 | *S7_48977293 |
| GH45 | *S7_49535506 |
| GH46 | *S7_53533106 |
| GH47 | *S7_53534384 |
| GH48 | *S7_54267331 |
| GH49 | *S7_55188117 |
| GH50 | *S7_56273823 |
| GH51 | *S7_57519849 |
| GH52 | *S7_58074437 |
| GH53 | *S7_58171875 |
| GH54 | *S7_58185716 |
| GH55 | *S7_59485872 |
| GH56 | *S7_59504314 |
| GH57 | *S7_59575589 |
| GH58 | *S7_59715818 |
| GH59 | *S7_60253043 |
| GH60 | *S7_60277646 |
| GH61 | *S7_61125687 |
| GH62 | *S7_61233731 |
| GH63 | *S7_61678445 |
| GH64 | *S7_61966197 |
| GH65 | *S7_62037462 |
| GH66 | *S7_62374745 |
| GH67 | *S7_62434017 |
| GH68 | *S7_62456533 |
| GH69 | *S7_62548356 |
| GH70 | *S7_62548411 |
| GH71 | *S7_62793617 |
| GH72 | *S7_62802228 |
| GH73 | *S7_62858132 |
| GH74 | *S7_62907345 |
| GH75 | *S7_62917764 |
| GH76 | *S7_63260415 |
| GH77 | *S7_63594700 |
| GH78 | *S7_63641077 |
| GH79 | *S7_63738074 |
| GH80 | *S7_63870518 |
| GH81 | *S7_64067105 |
| GH82 | *S7_64223276 |
| GH83 | *S7_64242497 |
| GH84 | *S7_64242513 |
| GH85 | *S7_3875125 |
| GH86 | *S7_386930 |
| GH87 | *S7_386951 |
| GH88 | *S7_421674 |
| GH89 | *S7_719717 |
| GH90 | *S7_910930 |
| GH91 | *S7_913882 |
| GH92 | *S7_913912 |
| GH93 | *S7_1105552 |
| GH94 | *S7_2131992 |
| GH95 | *S7_2235021 |
| GH96 | *S7_2675772 |
| GH97 | *S7_2675774 |
| GH98 | *S7_2681421 |
| GH99 | *S7_3769612 |
| GH100 | *S7_3785168 |
| GH101 | *S7_3785206 |
| GH102 | *S7_3785216 |
| GH103 | *S7_3861690 |
| GH104 | *S7_4074643 |
| GH105 | *S7_4530653 |
| GH106 | *S7_4723173 |
| GH107 | *S7_4723184 |
| GH108 | *S7_4977424 |
| GH109 | *S7_5185284 |
| GH110 | *S7_5879174 |
| GH111 | *S7_5935565 |
| GH112 | *S7_5952131 |
| GH113 | *S7_6521837 |
| GH114 | *S7_7079956 |
| GH115 | *S7_7242345 |
| GH116 | *S7_7467447 |
| GH117 | *S7_7467454 |
| GH118 | *S7_7467463 |
| GH119 | *S7_7467484 |
| GH120 | *S7_8853113 |
| GH121 | *S7_9908182 |
| GH122 | *S7_9985141 |
| GH123 | *S7_10384051 |
| GH124 | *S7_10384061 |
| GH125 | *S7_10384104 |
| GH126 | *S7_14196203 |
| GH127 | *S7_14196545 |
| GH128 | *S7_14444058 |
| GH129 | *S7_14444059 |
| GH130 | *S7_17020843 |
| GH131 | *S7_19586174 |
| GH132 | *S7_22295773 |
| GH133 | *S7_23026654 |
| GH134 | *S7_24643226 |
| GH135 | *S7_25488783 |
| GH136 | *S7_26026494 |
| GH137 | *S7_35375241 |
| GH138 | *S7_35449458 |
| GH139 | *S7_38740011 |
| GH140 | *S7_39350302 |
| GH141 | *S7_39992169 |
| GH142 | *S7_49160558 |
| GH143 | *S7_50877420 |
| GH144 | *S7_53534382 |
| GH145 | *S7_54738645 |
| GH146 | *S7_54744818 |
| GH147 | *S7_54988859 |
| GH148 | *S7_55242118 |
| GH149 | *S7_55366415 |
| GH150 | *S7_55378399 |
| GH151 | *S7_55766995 |
| GH152 | *S7_55892589 |
| GH153 | *S7_56624614 |
| GH154 | *S7_56624965 |
| GH155 | *S7_57116581 |
| GH156 | *S7_57116583 |
| GH157 | *S7_57116587 |
| GH158 | *S7_57325958 |
| GH159 | *S7_57876443 |
| GH160 | *S7_58178505 |
| GH161 | *S7_58185681 |
| GH162 | *S7_58198932 |
| GH163 | *S7_58733153 |
| GH164 | *S7_59228090 |
| GH165 | *S7_59340338 |
| GH166 | *S7_59483361 |
| GH167 | *S7_59503132 |
| GH168 | *S7_59751741 |
| GH169 | *S7_60983676 |
| GH170 | *S7_61034924 |
| GH171 | *S7_61075807 |
| GH172 | *S7_61125680 |
| GH173 | *S7_61233729 |
| GH174 | *S7_61621785 |
| GH175 | *S7_61621787 |
| GH176 | *S7_61890764 |
| GH177 | *S7_61928052 |
| GH178 | *S7_61928054 |
| GH179 | *S7_61966171 |
| GH180 | *S7_61966199 |
| GH181 | *S7_62012741 |
| GH182 | *S7_62434021 |
| GH183 | *S7_62551903 |
| GH184 | *S7_62560012 |
| GH185 | *S7_62793619 |
| GH186 | *S7_62858111 |
| GH187 | *S7_62968693 |
| GH188 | *S7_62968697 |
| GH189 | *S7_63841230 |
| GH190 | *S7_63893085 |
| GH191 | *S7_64163018 |
| GH192 | *S7_129403 |
| GH193 | *S7_171848 |
| GH194 | *S7_386904 |
| GH195 | *S7_386964 |
| GH196 | *S7_484319 |
| GH197 | *S7_504985 |
| GH198 | *S7_517937 |
| GH199 | *S7_2101527 |
| GH200 | *S7_2101528 |
| GH201 | *S7_2998506 |
| GH202 | *S7_3049684 |
| GH203 | *S7_3230966 |
| GH204 | *S7_3867834 |
| GH205 | *S7_3867882 |
| GH206 | *S7_4119839 |
| GH207 | *S7_4533400 |
| GH208 | *S7_4594199 |
| GH209 | *S7_5137384 |
| GH210 | *S7_5137390 |
| GH211 | *S7_5935562 |
| GH212 | *S7_5952104 |
| GH213 | *S7_7022057 |
| GH214 | *S7_8853122 |
| GH215 | *S7_9625031 |
| GH216 | *S7_9666372 |
| GH217 | *S7_9996426 |
| GH218 | *S7_13801876 |
| GH219 | *S7_15947367 |
| GH220 | *S7_16474458 |
| GH221 | *S7_25261749 |
| GH222 | *S7_25488751 |
| GH223 | *S7_38384208 |
| GH224 | *S7_38505837 |
| GH225 | *S7_38774236 |
| GH226 | *S7_39680832 |
| GH227 | *S7_39903260 |
| GH228 | *S7_40275026 |
| GH229 | *S7_52684443 |
| GH230 | *S7_53533062 |
| GH231 | *S7_53533140 |
| GH232 | *S7_53533164 |
| GH233 | *S7_53608370 |
| GH234 | *S7_54237230 |
| GH235 | *S7_55202510 |
| GH236 | *S7_55371380 |
| GH237 | *S7_55463753 |
| GH238 | *S7_55766990 |
| GH239 | *S7_55767025 |
| GH240 | *S7_55967308 |
| GH241 | *S7_56594636 |
| GH242 | *S7_56695848 |
| GH243 | *S7_56758852 |
| GH244 | *S7_57116599 |
| GH245 | *S7_57627040 |
| GH246 | *S7_58080017 |
| GH247 | *S7_58174750 |
| GH248 | *S7_58178527 |
| GH249 | *S7_58595811 |
| GH250 | *S7_58733195 |
| GH251 | *S7_58795431 |
| GH252 | *S7_59179199 |
| GH253 | *S7_59241922 |
| GH254 | *S7_59947023 |
| GH255 | *S7_60182649 |
| GH256 | *S7_60183504 |
| GH257 | *S7_60277652 |
| GH258 | *S7_60713313 |
| GH259 | *S7_61125688 |
| GH260 | *S7_61517000 |
| GH261 | *S7_61541440 |
| GH262 | *S7_61561166 |
| GH263 | *S7_61677243 |
| GH264 | *S7_61907067 |
| GH265 | *S7_61912326 |
| GH266 | *S7_61944722 |
| GH267 | *S7_62099626 |
| GH268 | *S7_62495034 |
| GH269 | *S7_62551904 |
| GH270 | *S7_62551906 |
| GH271 | *S7_62603320 |
| GH272 | *S7_62861226 |
| GH273 | *S7_62968695 |
| GH274 | *S7_62968696 |
| GH275 | *S7_63394597 |
| GH276 | *S7_63746534 |
| GH277 | *S7_64160398 |
| GH278 | *S7_64163011 |
| GH279 | *S7_64185791 |
| GH280 | *S7_64242503 |
| GH281 | *S7_64242518 |
| GH282 | *S7_59949371 |
| GH283 | *S7_20761657 |
| GH284 | *S7_380809 |
| GH285 | *S7_386941 |
| GH286 | *S7_466347 |
| GH287 | *S7_466540 |
| GH288 | *S7_504990 |
| GH289 | *S7_505982 |
| GH290 | *S7_1252902 |
| GH291 | *S7_2161297 |
| GH292 | *S7_2206636 |
| GH293 | *S7_3049631 |
| GH294 | *S7_3769098 |
| GH295 | *S7_3865141 |
| GH296 | *S7_3865142 |
| GH297 | *S7_3867721 |
| GH298 | *S7_3867844 |
| GH299 | *S7_3867890 |
| GH300 | *S7_4594249 |
| GH301 | *S7_5646042 |
| GH302 | *S7_6402311 |
| GH303 | *S7_7022045 |
| GH304 | *S7_7022135 |
| GH305 | *S7_7242353 |
| GH306 | *S7_7467471 |
| GH307 | *S7_7467510 |
| GH308 | *S7_8871617 |
| GH309 | *S7_9639287 |
| GH310 | *S7_12220116 |
| GH311 | *S7_12348535 |
| GH312 | *S7_14156326 |
| GH313 | *S7_16078239 |
| GH314 | *S7_16734781 |
| GH315 | *S7_19122331 |
| GH316 | *S7_22295765 |
| GH317 | *S7_22295800 |
| GH318 | *S7_22356909 |
| GH319 | *S7_29051873 |
| GH320 | *S7_29069880 |
| GH321 | *S7_35989324 |
| GH322 | *S7_38421543 |
| GH323 | *S7_38774244 |
| GH324 | *S7_38805503 |
| GH325 | *S7_39903288 |
| GH326 | *S7_40976377 |
| GH327 | *S7_41712976 |
| GH328 | *S7_42488789 |
| GH329 | *S7_47751816 |
| GH330 | *S7_48837070 |
| GH331 | *S7_48988930 |
| GH332 | *S7_49906676 |
| GH333 | *S7_53533142 |
| GH334 | *S7_53608366 |
| GH335 | *S7_54778890 |
| GH336 | *S7_55196075 |
| GH337 | *S7_55480180 |
| GH338 | *S7_55576248 |
| GH339 | *S7_57116589 |
| GH340 | *S7_57133161 |
| GH341 | *S7_57390315 |
| GH342 | *S7_57532550 |
| GH343 | *S7_58301965 |
| GH344 | *S7_59088035 |
| GH345 | *S7_59091597 |
| GH346 | *S7_59228091 |
| GH347 | *S7_59504266 |
| GH348 | *S7_59759249 |
| GH349 | *S7_61683973 |
| GH350 | *S7_61928053 |
| GH351 | *S7_61928071 |
| GH352 | *S7_62438587 |
| GH353 | *S7_62505004 |
| GH354 | *S7_62525898 |
| GH355 | *S7_62525901 |
| GH356 | *S7_62548412 |
| GH357 | *S7_62548600 |
| GH358 | *S7_62551896 |
| GH359 | *S7_62793620 |
| GH360 | *S7_62793622 |
| GH361 | *S7_62835527 |
| GH362 | *S7_62835572 |
| GH363 | *S7_63183755 |
| GH364 | *S7_63519927 |
| GH365 | *S7_63738016 |
| GH366 | *S7_64220344 |
| GH367 | *S7_56145255 |
| JK11 | *S8_675364 |
| JK12 | *S8_747044 |
| JK13 | *S8_749491 |
| JK14 | *S8_3176563 |
| JK15 | *S8_3450164 |
| JK16 | *S8_3709167 |
| JK17 | *S8_3709427 |
| JK18 | *S8_3895459 |
| JK19 | *S8_4306997 |
| JK20 | *S8_4307000 |
| JK21 | *S8_4307005 |
| JK22 | *S8_4307007 |
| JK23 | *S8_4307008 |
| JK24 | *S8_4599655 |
| JK25 | *S8_4599656 |
| JK26 | *S8_5028090 |
| JK27 | *S8_5348970 |
| JK28 | *S8_5414911 |
| JK29 | *S8_5512280 |
| JK30 | *S8_5597619 |
| JK31 | *S8_5922377 |
| JK32 | *S8_5935863 |
| JK33 | *S8_6112798 |
| JK34 | *S8_6198421 |
| JK35 | *S8_6317425 |
| JK36 | *S8_6317427 |
| JK37 | *S8_6949088 |
| JK38 | *S8_7589250 |
| JK39 | *S8_7744294 |
| JK40 | *S8_8717553 |
| JK41 | *S8_9443687 |
| JK42 | *S8_9486409 |
| JK43 | *S8_17082694 |
| JK44 | *S8_18149886 |
| JK45 | *S8_23402332 |
| JK46 | *S8_25059454 |
| JK47 | *S8_27000541 |
| JK48 | *S8_31979335 |
| JK49 | *S8_35010996 |
| JK50 | *S8_35114227 |
| JK51 | *S8_38580875 |
| JK52 | *S8_40413588 |
| JK53 | *S8_42220292 |
| JK54 | *S8_42366635 |
| JK55 | *S8_42484226 |
| JK56 | *S8_43252194 |
| JK57 | *S8_43660714 |
| JK58 | *S8_43660741 |
| JK59 | *S8_43810766 |
| JK60 | *S8_44317555 |
| JK61 | *S8_44410560 |
| JK62 | *S8_44868554 |
| JK63 | *S8_44895433 |
| JK64 | *S8_48876418 |
| JK65 | *S8_49763923 |
| JK66 | *S8_49835698 |
| JK67 | *S8_50253156 |
| JK68 | *S8_50763114 |
| JK69 | *S8_51557158 |
| JK70 | *S8_51595429 |
| JK71 | *S8_51816599 |
| JK72 | *S8_52116821 |
| JK73 | *S8_52163953 |
| JK74 | *S8_52286828 |
| JK75 | *S8_52747459 |
| JK76 | *S8_52747463 |
| JK77 | *S8_52905782 |
| JK78 | *S8_53012894 |
| JK79 | *S8_53012931 |
| JK80 | *S8_53012949 |
| JK81 | *S8_53597033 |
| JK82 | *S8_53935448 |
| JK83 | *S8_54817927 |
| JK84 | *S8_54945177 |
| JK85 | *S8_55141031 |
| JK86 | *S8_55150698 |
| JK87 | *S8_55210120 |
| JK88 | *S8_55270659 |
| JK89 | *S8_55296517 |
| JK90 | *S8_55296520 |
| JK91 | *S8_55400831 |
| JK92 | *S8_630076 |
| JK93 | *S8_634115 |
| JK94 | *S8_749489 |
| JK95 | *S8_843646 |
| JK96 | *S8_2386773 |
| JK97 | *S8_2989128 |
| JK98 | *S8_3681688 |
| JK99 | *S8_3894511 |
| JK100 | *S8_3894512 |
| JK101 | *S8_3894513 |
| JK102 | *S8_4172210 |
| JK103 | *S8_4336298 |
| JK104 | *S8_4336319 |
| JK105 | *S8_4336322 |
| JK106 | *S8_4336323 |
| JK107 | *S8_4336329 |
| JK108 | *S8_4336335 |
| JK109 | *S8_4599637 |
| JK110 | *S8_4599654 |
| JK111 | *S8_4849890 |
| JK112 | *S8_5034481 |
| JK113 | *S8_5077247 |
| JK114 | *S8_5095248 |
| JK115 | *S8_5348948 |
| JK116 | *S8_5477013 |
| JK117 | *S8_5512300 |
| JK118 | *S8_5597621 |
| JK119 | *S8_5597624 |
| JK120 | *S8_6057127 |
| JK121 | *S8_6196402 |
| JK122 | *S8_6198420 |
| JK123 | *S8_6228057 |
| JK124 | *S8_6317623 |
| JK125 | *S8_6448105 |
| JK126 | *S8_6828395 |
| JK127 | *S8_6828454 |
| JK128 | *S8_7783679 |
| JK129 | *S8_7818674 |
| JK130 | *S8_8168044 |
| JK131 | *S8_9146364 |
| JK132 | *S8_11342132 |
| JK133 | *S8_16329684 |
| JK134 | *S8_16760688 |
| JK135 | *S8_16929657 |
| JK136 | *S8_23644806 |
| JK137 | *S8_30568133 |
| JK138 | *S8_34230453 |
| JK139 | *S8_37266103 |
| JK140 | *S8_38300606 |
| JK141 | *S8_38356592 |
| JK142 | *S8_40529184 |
| JK143 | *S8_41212672 |
| JK144 | *S8_41273241 |
| JK145 | *S8_41924700 |
| JK146 | *S8_42048049 |
| JK147 | *S8_42235517 |
| JK148 | *S8_42264937 |
| JK149 | *S8_42291420 |
| JK150 | *S8_42402887 |
| JK151 | *S8_42852057 |
| JK152 | *S8_42986341 |
| JK153 | *S8_43178413 |
| JK154 | *S8_43810764 |
| JK155 | *S8_43935634 |
| JK156 | *S8_43975018 |
| JK157 | *S8_43975062 |
| JK158 | *S8_44410543 |
| JK159 | *S8_44713995 |
| JK160 | *S8_44714000 |
| JK161 | *S8_46514787 |
| JK162 | *S8_46778485 |
| JK163 | *S8_47120555 |
| JK164 | *S8_47807610 |
| JK165 | *S8_48876389 |
| JK166 | *S8_49225134 |
| JK167 | *S8_49284631 |
| JK168 | *S8_49643701 |
| JK169 | *S8_50085786 |
| JK170 | *S8_51595434 |
| JK171 | *S8_52053595 |
| JK172 | *S8_52117348 |
| JK173 | *S8_52286861 |
| JK174 | *S8_52286910 |
| JK175 | *S8_52310547 |
| JK176 | *S8_52747461 |
| JK177 | *S8_52747462 |
| JK178 | *S8_52747464 |
| JK179 | *S8_52905746 |
| JK180 | *S8_52957753 |
| JK181 | *S8_53012897 |
| JK182 | *S8_53137116 |
| JK183 | *S8_53510880 |
| JK184 | *S8_53600991 |
| JK185 | *S8_53646343 |
| JK186 | *S8_53831082 |
| JK187 | *S8_53937570 |
| JK188 | *S8_54023532 |
| JK189 | *S8_54023533 |
| JK190 | *S8_54023536 |
| JK191 | *S8_54023538 |
| JK192 | *S8_54414177 |
| JK193 | *S8_54474351 |
| JK194 | *S8_54474361 |
| JK195 | *S8_54647165 |
| JK196 | *S8_54647168 |
| JK197 | *S8_54647171 |
| JK198 | *S8_54654404 |
| JK199 | *S8_54686244 |
| JK200 | *S8_54686266 |
| JK201 | *S8_54686295 |
| JK202 | *S8_54719394 |
| JK203 | *S8_54872632 |
| JK204 | *S8_54889758 |
| JK205 | *S8_55182814 |
| JK206 | *S8_8998043 |
| JK207 | *S8_644157 |
| JK208 | *S8_670328 |
| JK209 | *S8_685987 |
| JK210 | *S8_747060 |
| JK211 | *S8_747348 |
| JK212 | *S8_1978490 |
| JK213 | *S8_3153893 |
| JK214 | *S8_3274095 |
| JK215 | *S8_3424340 |
| JK216 | *S8_3894514 |
| JK217 | *S8_3895460 |
| JK218 | *S8_3895486 |
| JK219 | *S8_4210787 |
| JK220 | *S8_4236026 |
| JK221 | *S8_4307003 |
| JK222 | *S8_4307004 |
| JK223 | *S8_4336314 |
| JK224 | *S8_4336321 |
| JK225 | *S8_4599636 |
| JK226 | *S8_4599638 |
| JK227 | *S8_4920622 |
| JK228 | *S8_4920655 |
| JK229 | *S8_5028083 |
| JK230 | *S8_5086770 |
| JK231 | *S8_5086809 |
| JK232 | *S8_5597622 |
| JK233 | *S8_6057130 |
| JK234 | *S8_6198479 |
| JK235 | *S8_6198491 |
| JK236 | *S8_7341357 |
| JK237 | *S8_7449688 |
| JK238 | *S8_7608135 |
| JK239 | *S8_7783644 |
| JK240 | *S8_8189326 |
| JK241 | *S8_8437994 |
| JK242 | *S8_11342134 |
| JK243 | *S8_15903231 |
| JK244 | *S8_15908914 |
| JK245 | *S8_16329653 |
| JK246 | *S8_16480428 |
| JK247 | *S8_16929703 |
| JK248 | *S8_22836805 |
| JK249 | *S8_28536792 |
| JK250 | *S8_28677123 |
| JK251 | *S8_32111273 |
| JK252 | *S8_32264036 |
| JK253 | *S8_33316336 |
| JK254 | *S8_35875680 |
| JK255 | *S8_37784195 |
| JK256 | *S8_38300595 |
| JK257 | *S8_38580862 |
| JK258 | *S8_38580886 |
| JK259 | *S8_39071155 |
| JK260 | *S8_39071164 |
| JK261 | *S8_39419685 |
| JK262 | *S8_41621163 |
| JK263 | *S8_41622040 |
| JK264 | *S8_41756003 |
| JK265 | *S8_42264897 |
| JK266 | *S8_42984764 |
| JK267 | *S8_43765622 |
| JK268 | *S8_43765626 |
| JK269 | *S8_43810740 |
| JK270 | *S8_43917069 |
| JK271 | *S8_43935611 |
| JK272 | *S8_43975063 |
| JK273 | *S8_43991205 |
| JK274 | *S8_44714002 |
| JK275 | *S8_44734488 |
| JK276 | *S8_45081225 |
| JK277 | *S8_45132157 |
| JK278 | *S8_46531829 |
| JK279 | *S8_46531857 |
| JK280 | *S8_46971950 |
| JK281 | *S8_47476075 |
| JK282 | *S8_49287477 |
| JK283 | *S8_49324392 |
| JK284 | *S8_50082857 |
| JK285 | *S8_50085770 |
| JK286 | *S8_50085795 |
| JK287 | *S8_51061776 |
| JK288 | *S8_51061778 |
| JK289 | *S8_51263977 |
| JK290 | *S8_51291828 |
| JK291 | *S8_51726600 |
| JK292 | *S8_51810020 |
| JK293 | *S8_52092534 |
| JK294 | *S8_52117347 |
| JK295 | *S8_52219100 |
| JK296 | *S8_52286829 |
| JK297 | *S8_52286913 |
| JK298 | *S8_52302185 |
| JK299 | *S8_52393326 |
| JK300 | *S8_52399884 |
| JK301 | *S8_52747454 |
| JK302 | *S8_53012947 |
| JK303 | *S8_53106507 |
| JK304 | *S8_53319882 |
| JK305 | *S8_53926746 |
| JK306 | *S8_53926751 |
| JK307 | *S8_54132498 |
| JK308 | *S8_54321884 |
| JK309 | *S8_54398769 |
| JK310 | *S8_54417665 |
| JK311 | *S8_54417673 |
| JK312 | *S8_54434866 |
| JK313 | *S8_54434869 |
| JK314 | *S8_54647166 |
| JK315 | *S8_54647169 |
| JK316 | *S8_54680686 |
| JK317 | *S8_54719395 |
| JK318 | *S8_54719414 |
| JK319 | *S8_54945186 |
| JK320 | *S8_54989996 |
| JK321 | *S8_55111093 |
| JK322 | *S8_55130931 |
| JK323 | *S8_55172882 |
| JK324 | *S8_55400830 |
| JK325 | *S8_2117491 |
| JK326 | *S8_3513493 |
| JK327 | *S8_8998040 |
| JK328 | *S8_8998042 |
| JK329 | *S8_930721 |
| JK330 | *S8_3173873 |
| JK331 | *S8_3176568 |
| JK332 | *S8_4306998 |
| JK333 | *S8_4307006 |
| JK334 | *S8_4336325 |
| JK335 | *S8_4336332 |
| JK336 | *S8_4642875 |
| JK337 | *S8_4920623 |
| JK338 | *S8_5095283 |
| JK339 | *S8_5414912 |
| JK340 | *S8_5721341 |
| JK341 | *S8_6057126 |
| JK342 | *S8_6057128 |
| JK343 | *S8_6111905 |
| JK344 | *S8_6196432 |
| JK345 | *S8_6198481 |
| JK346 | *S8_6198482 |
| JK347 | *S8_6226242 |
| JK348 | *S8_6642715 |
| JK349 | *S8_7818695 |
| JK350 | *S8_7970814 |
| JK351 | *S8_9178352 |
| JK352 | *S8_9182130 |
| JK353 | *S8_9301648 |
| JK354 | *S8_9435152 |
| JK355 | *S8_16329669 |
| JK356 | *S8_16929709 |
| JK357 | *S8_17134609 |
| JK358 | *S8_17372246 |
| JK359 | *S8_26161386 |
| JK360 | *S8_31979369 |
| JK361 | *S8_33801177 |
| JK362 | *S8_34230498 |
| JK363 | *S8_35875652 |
| JK364 | *S8_37784223 |
| JK365 | *S8_37806448 |
| JK366 | *S8_38299390 |
| JK367 | *S8_41604685 |
| JK368 | *S8_41621196 |
| JK369 | *S8_42220332 |
| JK370 | *S8_42264904 |
| JK371 | *S8_42291049 |
| JK372 | *S8_42402866 |
| JK373 | *S8_42402871 |
| JK374 | *S8_42679446 |
| JK375 | *S8_44074091 |
| JK376 | *S8_44210888 |
| JK377 | *S8_44582306 |
| JK378 | *S8_44876065 |
| JK379 | *S8_46531815 |
| JK380 | *S8_46998525 |
| JK381 | *S8_49031245 |
| JK382 | *S8_50082654 |
| JK383 | *S8_50082660 |
| JK384 | *S8_50085764 |
| JK385 | *S8_50244656 |
| JK386 | *S8_51061803 |
| JK387 | *S8_51279010 |
| JK388 | *S8_51279094 |
| JK389 | *S8_52021946 |
| JK390 | *S8_52092792 |
| JK391 | *S8_52117345 |
| JK392 | *S8_52286837 |
| JK393 | *S8_52286894 |
| JK394 | *S8_52339438 |
| JK395 | *S8_52450447 |
| JK396 | *S8_52450451 |
| JK397 | *S8_52747456 |
| JK398 | *S8_52747479 |
| JK399 | *S8_52885876 |
| JK400 | *S8_52897322 |
| JK401 | *S8_52905789 |
| JK402 | *S8_52953238 |
| JK403 | *S8_52992873 |
| JK404 | *S8_53098802 |
| JK405 | *S8_53106512 |
| JK406 | *S8_53117743 |
| JK407 | *S8_53193766 |
| JK408 | *S8_53326025 |
| JK409 | *S8_53597039 |
| JK410 | *S8_53597042 |
| JK411 | *S8_53661225 |
| JK412 | *S8_54023535 |
| JK413 | *S8_54321891 |
| JK414 | *S8_54434867 |
| JK415 | *S8_54474352 |
| JK416 | *S8_54647164 |
| JK417 | *S8_54647167 |
| JK418 | *S8_54647170 |
| JK419 | *S8_54924334 |
| JK420 | *S8_55172897 |
| JK421 | *S8_55309094 |
| JK422 | *S8_55400832 |
| JK423 | *S8_55400834 |
| BF05 | *S9_97075 |
| BF06 | *S9_368807 |
| BF07 | *S9_512306 |
| BF08 | *S9_572973 |
| BF09 | *S9_928458 |
| BF10 | *S9_979845 |
| BF11 | *S9_983412 |
| BF12 | *S9_1804231 |
| BF13 | *S9_1804255 |
| BF14 | *S9_1844995 |
| BF15 | *S9_1960269 |
| BF16 | *S9_2452736 |
| BF17 | *S9_2513275 |
| BF18 | *S9_2710319 |
| BF19 | *S9_2769681 |
| BF20 | *S9_2778589 |
| BF21 | *S9_2990415 |
| BF22 | *S9_3016194 |
| BF23 | *S9_5616727 |
| BF24 | *S9_6276921 |
| BF25 | *S9_10779994 |
| BF26 | *S9_15761650 |
| BF27 | *S9_26051199 |
| BF28 | *S9_35842882 |
| BF29 | *S9_35842887 |
| BF30 | *S9_36881078 |
| BF31 | *S9_36881092 |
| BF32 | *S9_39925352 |
| BF33 | *S9_42299438 |
| BF34 | *S9_42772348 |
| BF35 | *S9_43223764 |
| BF36 | *S9_44108072 |
| BF37 | *S9_45778336 |
| BF38 | *S9_47946262 |
| BF39 | *S9_50072511 |
| BF40 | *S9_50364886 |
| BF41 | *S9_50387175 |
| BF42 | *S9_50387813 |
| BF43 | *S9_50446611 |
| BF44 | *S9_51079595 |
| BF45 | *S9_51381549 |
| BF46 | *S9_51838000 |
| BF47 | *S9_51969120 |
| BF48 | *S9_52092255 |
| BF49 | *S9_52097811 |
| BF50 | *S9_52876216 |
| BF51 | *S9_52876220 |
| BF52 | *S9_52876221 |
| BF53 | *S9_52876222 |
| BF54 | *S9_52883259 |
| BF55 | *S9_52988355 |
| BF56 | *S9_53122205 |
| BF57 | *S9_53578793 |
| BF58 | *S9_54267985 |
| BF59 | *S9_54557385 |
| BF60 | *S9_54668240 |
| BF61 | *S9_54836651 |
| BF62 | *S9_54878726 |
| BF63 | *S9_54946597 |
| BF64 | *S9_55342131 |
| BF65 | *S9_55378398 |
| BF66 | *S9_55406152 |
| BF67 | *S9_55562556 |
| BF68 | *S9_56346239 |
| BF69 | *S9_56420674 |
| BF70 | *S9_56420676 |
| BF71 | *S9_56517324 |
| BF72 | *S9_56670945 |
| BF73 | *S9_56884983 |
| BF74 | *S9_57144661 |
| BF75 | *S9_57144670 |
| BF76 | *S9_57404844 |
| BF77 | *S9_57418456 |
| BF78 | *S9_57770922 |
| BF79 | *S9_57773092 |
| BF80 | *S9_57858123 |
| BF81 | *S9_57975392 |
| BF82 | *S9_57982585 |
| BF83 | *S9_58078019 |
| BF84 | *S9_58655786 |
| BF85 | *S9_58659436 |
| BF86 | *S9_58821952 |
| BF87 | *S9_58821960 |
| BF88 | *S9_58856468 |
| BF89 | *S9_58949178 |
| BF90 | *S9_58974963 |
| BF91 | *S9_59369436 |
| BF92 | *S9_59527209 |
| BF93 | *S9_59533253 |
| BF94 | *S9_59587124 |
| BF95 | *S9_57975442 |
| BF96 | *S9_290536 |
| BF97 | *S9_486428 |
| BF98 | *S9_538905 |
| BF99 | *S9_775883 |
| BF100 | *S9_775909 |
| BF101 | *S9_775932 |
| BF102 | *S9_928456 |
| BF103 | *S9_928476 |
| BF104 | *S9_928480 |
| BF105 | *S9_928488 |
| BF106 | *S9_928489 |
| BF107 | *S9_1099509 |
| BF108 | *S9_1099801 |
| BF109 | *S9_1200883 |
| BF110 | *S9_1759841 |
| BF111 | *S9_1840324 |
| BF112 | *S9_2472604 |
| BF113 | *S9_2513277 |
| BF114 | *S9_2745689 |
| BF115 | *S9_3549806 |
| BF116 | *S9_3731791 |
| BF117 | *S9_3940796 |
| BF118 | *S9_3984204 |
| BF119 | *S9_4129570 |
| BF120 | *S9_5564501 |
| BF121 | *S9_7168646 |
| BF122 | *S9_10169916 |
| BF123 | *S9_16327369 |
| BF124 | *S9_17676963 |
| BF125 | *S9_22805033 |
| BF126 | *S9_25290161 |
| BF127 | *S9_36881099 |
| BF128 | *S9_39925341 |
| BF129 | *S9_39925350 |
| BF130 | *S9_39925355 |
| BF131 | *S9_40148567 |
| BF132 | *S9_40545413 |
| BF133 | *S9_40715674 |
| BF134 | *S9_40715771 |
| BF135 | *S9_42123273 |
| BF136 | *S9_45545363 |
| BF137 | *S9_47301301 |
| BF138 | *S9_47700794 |
| BF139 | *S9_47742092 |
| BF140 | *S9_47946230 |
| BF141 | *S9_47946254 |
| BF142 | *S9_49795729 |
| BF143 | *S9_50446579 |
| BF144 | *S9_51377163 |
| BF145 | *S9_51969117 |
| BF146 | *S9_52052553 |
| BF147 | *S9_52092058 |
| BF148 | *S9_52876217 |
| BF149 | *S9_52876230 |
| BF150 | *S9_52978464 |
| BF151 | *S9_53115382 |
| BF152 | *S9_53115388 |
| BF153 | *S9_53115393 |
| BF154 | *S9_53238565 |
| BF155 | *S9_53359150 |
| BF156 | *S9_54225919 |
| BF157 | *S9_54866560 |
| BF158 | *S9_54879336 |
| BF159 | *S9_55378361 |
| BF160 | *S9_55407107 |
| BF161 | *S9_55562524 |
| BF162 | *S9_55562560 |
| BF163 | *S9_56306289 |
| BF164 | *S9_56346212 |
| BF165 | *S9_56371890 |
| BF166 | *S9_56411803 |
| BF167 | *S9_56420677 |
| BF168 | *S9_56420679 |
| BF169 | *S9_56467620 |
| BF170 | *S9_56517355 |
| BF171 | *S9_56637921 |
| BF172 | *S9_56667818 |
| BF173 | *S9_56667827 |
| BF174 | *S9_56818378 |
| BF175 | *S9_56889749 |
| BF176 | *S9_56997650 |
| BF177 | *S9_57158497 |
| BF178 | *S9_57231813 |
| BF179 | *S9_57492768 |
| BF180 | *S9_57516530 |
| BF181 | *S9_57605944 |
| BF182 | *S9_57982636 |
| BF183 | *S9_58045285 |
| BF184 | *S9_58085552 |
| BF185 | *S9_58085631 |
| BF186 | *S9_58659453 |
| BF187 | *S9_58821950 |
| BF188 | *S9_58867543 |
| BF189 | *S9_59500589 |
| BF190 | *S9_56850165 |
| BF191 | *S9_56850191 |
| BF192 | *S9_159843 |
| BF193 | *S9_928486 |
| BF194 | *S9_1099506 |
| BF195 | *S9_1099802 |
| BF196 | *S9_1104111 |
| BF197 | *S9_1209452 |
| BF198 | *S9_1846566 |
| BF199 | *S9_1953727 |
| BF200 | *S9_1960260 |
| BF201 | *S9_2083811 |
| BF202 | *S9_2513273 |
| BF203 | *S9_2513276 |
| BF204 | *S9_2557086 |
| BF205 | *S9_2700336 |
| BF206 | *S9_2700354 |
| BF207 | *S9_3001489 |
| BF208 | *S9_3534305 |
| BF209 | *S9_3534311 |
| BF210 | *S9_3887049 |
| BF211 | *S9_4241103 |
| BF212 | *S9_4697915 |
| BF213 | *S9_5278492 |
| BF214 | *S9_5388239 |
| BF215 | *S9_10226265 |
| BF216 | *S9_10652885 |
| BF217 | *S9_35842883 |
| BF218 | *S9_39925353 |
| BF219 | *S9_39925362 |
| BF220 | *S9_40148573 |
| BF221 | *S9_40899212 |
| BF222 | *S9_42090710 |
| BF223 | *S9_42513985 |
| BF224 | *S9_47725958 |
| BF225 | *S9_47779426 |
| BF226 | *S9_47946226 |
| BF227 | *S9_49919072 |
| BF228 | *S9_50014117 |
| BF229 | *S9_50025508 |
| BF230 | *S9_50090470 |
| BF231 | *S9_50225732 |
| BF232 | *S9_50251629 |
| BF233 | *S9_51670308 |
| BF234 | *S9_51724859 |
| BF235 | *S9_52084087 |
| BF236 | *S9_52213065 |
| BF237 | *S9_52213072 |
| BF238 | *S9_52842814 |
| BF239 | *S9_52876214 |
| BF240 | *S9_52883258 |
| BF241 | *S9_52883260 |
| BF242 | *S9_52931532 |
| BF243 | *S9_52983077 |
| BF244 | *S9_53115390 |
| BF245 | *S9_53185523 |
| BF246 | *S9_53253073 |
| BF247 | *S9_53442340 |
| BF248 | *S9_53750007 |
| BF249 | *S9_54406443 |
| BF250 | *S9_54557430 |
| BF251 | *S9_54642105 |
| BF252 | *S9_54672900 |
| BF253 | *S9_54866551 |
| BF254 | *S9_55052216 |
| BF255 | *S9_55071888 |
| BF256 | *S9_55293755 |
| BF257 | *S9_55407308 |
| BF258 | *S9_55677895 |
| BF259 | *S9_55804757 |
| BF260 | *S9_56244127 |
| BF261 | *S9_56346216 |
| BF262 | *S9_56359167 |
| BF263 | *S9_56411816 |
| BF264 | *S9_56631727 |
| BF265 | *S9_56650618 |
| BF266 | *S9_56665746 |
| BF267 | *S9_56889753 |
| BF268 | *S9_57104209 |
| BF269 | *S9_57144649 |
| BF270 | *S9_57144651 |
| BF271 | *S9_57144660 |
| BF272 | *S9_57272027 |
| BF273 | *S9_57594058 |
| BF274 | *S9_57770907 |
| BF275 | *S9_57978373 |
| BF276 | *S9_57982627 |
| BF277 | *S9_58119175 |
| BF278 | *S9_58715133 |
| BF279 | *S9_58821955 |
| BF280 | *S9_58867541 |
| BF281 | *S9_59332526 |
| BF282 | *S9_58982608 |
| BF283 | *S9_159841 |
| BF284 | *S9_775885 |
| BF285 | *S9_928481 |
| BF286 | *S9_928484 |
| BF287 | *S9_928491 |
| BF288 | *S9_1066736 |
| BF289 | *S9_1074934 |
| BF290 | *S9_1817971 |
| BF291 | *S9_1845041 |
| BF292 | *S9_1846540 |
| BF293 | *S9_1846569 |
| BF294 | *S9_1960233 |
| BF295 | *S9_2472595 |
| BF296 | *S9_2513274 |
| BF297 | *S9_2565580 |
| BF298 | *S9_2773867 |
| BF299 | *S9_2890831 |
| BF300 | *S9_3001571 |
| BF301 | *S9_3001579 |
| BF302 | *S9_3165775 |
| BF303 | *S9_3200428 |
| BF304 | *S9_3955513 |
| BF305 | *S9_4010202 |
| BF306 | *S9_6304319 |
| BF307 | *S9_7185215 |
| BF308 | *S9_10652458 |
| BF309 | *S9_10659080 |
| BF310 | *S9_12723773 |
| BF311 | *S9_12723776 |
| BF312 | *S9_18590236 |
| BF313 | *S9_21216129 |
| BF314 | *S9_22074865 |
| BF315 | *S9_23174727 |
| BF316 | *S9_39925347 |
| BF317 | *S9_39925348 |
| BF318 | *S9_39925358 |
| BF319 | *S9_39925361 |
| BF320 | *S9_40715175 |
| BF321 | *S9_47713647 |
| BF322 | *S9_47779497 |
| BF323 | *S9_49812200 |
| BF324 | *S9_50063784 |
| BF325 | *S9_50173991 |
| BF326 | *S9_50387817 |
| BF327 | *S9_51079589 |
| BF328 | *S9_51079611 |
| BF329 | *S9_51381548 |
| BF330 | *S9_51381550 |
| BF331 | *S9_51381553 |
| BF332 | *S9_51664809 |
| BF333 | *S9_51725607 |
| BF334 | *S9_51943651 |
| BF335 | *S9_51969141 |
| BF336 | *S9_52176904 |
| BF337 | *S9_52213055 |
| BF338 | *S9_52530016 |
| BF339 | *S9_52883261 |
| BF340 | *S9_53018039 |
| BF341 | *S9_53115384 |
| BF342 | *S9_53115391 |
| BF343 | *S9_53208495 |
| BF344 | *S9_53238552 |
| BF345 | *S9_53324846 |
| BF346 | *S9_53442876 |
| BF347 | *S9_53443020 |
| BF348 | *S9_53597878 |
| BF349 | *S9_53707034 |
| BF350 | *S9_53735335 |
| BF351 | *S9_54315662 |
| BF352 | *S9_54373557 |
| BF353 | *S9_54557381 |
| BF354 | *S9_54722188 |
| BF355 | *S9_55320227 |
| BF356 | *S9_55407152 |
| BF357 | *S9_55407316 |
| BF358 | *S9_56338307 |
| BF359 | *S9_56359152 |
| BF360 | *S9_56420678 |
| BF361 | *S9_56593640 |
| BF362 | *S9_56631726 |
| BF363 | *S9_56631728 |
| BF364 | *S9_56650615 |
| BF365 | *S9_56665747 |
| BF366 | *S9_56667817 |
| BF367 | *S9_56667851 |
| BF368 | *S9_56670874 |
| BF369 | *S9_56727656 |
| BF370 | *S9_56993494 |
| BF371 | *S9_57175755 |
| BF372 | *S9_57210016 |
| BF373 | *S9_57210268 |
| BF374 | *S9_57424803 |
| BF375 | *S9_57503444 |
| BF376 | *S9_57585046 |
| BF377 | *S9_57782915 |
| BF378 | *S9_57858119 |
| BF379 | *S9_58077975 |
| BF380 | *S9_58119197 |
| BF381 | *S9_58659099 |
| BF382 | *S9_58762782 |
| BF383 | *S9_58865234 |
| BF384 | *S9_58865236 |
| BF385 | *S9_58865237 |
| BF386 | *S9_59125572 |
| BF387 | *S9_59125575 |
| BF388 | *S9_59369418 |
| BF389 | *S9_59425185 |
| BF390 | *S9_59452821 |
| BF391 | *S9_59452826 |
| BF392 | *S9_59502796 |
| BF393 | *S9_6004334 |
| CPS02 | *S10_415238 |
| CPS03 | *S10_420674 |
| CPS04 | *S10_420684 |
| CPS05 | *S10_644011 |
| CPS06 | *S10_715143 |
| CPS07 | *S10_780225 |
| CPS08 | *S10_794365 |
| CPS09 | *S10_984099 |
| CPS10 | *S10_1148656 |
| CPS11 | *S10_1188858 |
| CPS12 | *S10_1188861 |
| CPS13 | *S10_1188866 |
| CPS14 | *S10_1188870 |
| CPS15 | *S10_1230147 |
| CPS16 | *S10_1793888 |
| CPS17 | *S10_2470112 |
| CPS18 | *S10_2662732 |
| CPS19 | *S10_2857408 |
| CPS20 | *S10_3167522 |
| CPS21 | *S10_3200515 |
| CPS22 | *S10_3214686 |
| CPS23 | *S10_4157883 |
| CPS24 | *S10_4192317 |
| CPS25 | *S10_5374354 |
| CPS26 | *S10_5700918 |
| CPS27 | *S10_5903100 |
| CPS28 | *S10_5970339 |
| CPS29 | *S10_6438747 |
| CPS30 | *S10_6606364 |
| CPS31 | *S10_6781696 |
| CPS32 | *S10_6851098 |
| CPS33 | *S10_6938044 |
| CPS34 | *S10_6941945 |
| CPS35 | *S10_7377104 |
| CPS36 | *S10_7847258 |
| CPS37 | *S10_8246875 |
| CPS38 | *S10_8524839 |
| CPS39 | *S10_8721168 |
| CPS40 | *S10_8721200 |
| CPS41 | *S10_8804175 |
| CPS42 | *S10_8828770 |
| CPS43 | *S10_9163505 |
| CPS44 | *S10_9181175 |
| CPS45 | *S10_9195714 |
| CPS46 | *S10_9359123 |
| CPS47 | *S10_9421647 |
| CPS48 | *S10_9615618 |
| CPS49 | *S10_9615622 |
| CPS50 | *S10_10457987 |
| CPS51 | *S10_10677839 |
| CPS52 | *S10_10992066 |
| CPS53 | *S10_11025721 |
| CPS54 | *S10_12720806 |
| CPS55 | *S10_13013939 |
| CPS56 | *S10_13384074 |
| CPS57 | *S10_14817512 |
| CPS58 | *S10_15068088 |
| CPS59 | *S10_15371099 |
| CPS60 | *S10_15810795 |
| CPS61 | *S10_18201666 |
| CPS62 | *S10_18544172 |
| CPS63 | *S10_18640784 |
| CPS64 | *S10_18640792 |
| CPS65 | *S10_21761675 |
| CPS66 | *S10_21786867 |
| CPS67 | *S10_24857141 |
| CPS68 | *S10_25048953 |
| CPS69 | *S10_28037741 |
| CPS70 | *S10_33814501 |
| CPS71 | *S10_34428853 |
| CPS72 | *S10_37970421 |
| CPS73 | *S10_39979752 |
| CPS74 | *S10_39979757 |
| CPS75 | *S10_40037932 |
| CPS76 | *S10_40037980 |
| CPS77 | *S10_43199191 |
| CPS78 | *S10_43273163 |
| CPS79 | *S10_45364838 |
| CPS80 | *S10_46207408 |
| CPS81 | *S10_46589343 |
| CPS82 | *S10_47617478 |
| CPS83 | *S10_47834766 |
| CPS84 | *S10_48901592 |
| CPS85 | *S10_49098542 |
| CPS86 | *S10_49342256 |
| CPS87 | *S10_49435781 |
| CPS88 | *S10_49639849 |
| CPS89 | *S10_50707442 |
| CPS90 | *S10_51141983 |
| CPS91 | *S10_53832815 |
| CPS92 | *S10_54032086 |
| CPS93 | *S10_55146993 |
| CPS94 | *S10_55360870 |
| CPS95 | *S10_55376812 |
| CPS96 | *S10_56237838 |
| CPS97 | *S10_56736551 |
| CPS98 | *S10_57052570 |
| CPS99 | *S10_57190744 |
| CPS100 | *S10_57307301 |
| CPS101 | *S10_57341007 |
| CPS102 | *S10_57592933 |
| CPS103 | *S10_58154910 |
| CPS104 | *S10_58345049 |
| CPS105 | *S10_58460662 |
| CPS106 | *S10_58538418 |
| CPS107 | *S10_59082132 |
| CPS108 | *S10_59221674 |
| CPS109 | *S10_59336041 |
| CPS110 | *S10_59342820 |
| CPS111 | *S10_59723940 |
| CPS112 | *S10_59820267 |
| CPS113 | *S10_60283151 |
| CPS114 | *S10_60324252 |
| CPS115 | *S10_60507675 |
| CPS116 | *S10_60561096 |
| CPS117 | *S10_60561099 |
| CPS118 | *S10_415257 |
| CPS119 | *S10_424088 |
| CPS120 | *S10_439509 |
| CPS121 | *S10_584833 |
| CPS122 | *S10_959430 |
| CPS123 | *S10_1073152 |
| CPS124 | *S10_1079281 |
| CPS125 | *S10_1156686 |
| CPS126 | *S10_1158839 |
| CPS127 | *S10_1221862 |
| CPS128 | *S10_1230117 |
| CPS129 | *S10_1567848 |
| CPS130 | *S10_1651361 |
| CPS131 | *S10_2102463 |
| CPS132 | *S10_2662700 |
| CPS133 | *S10_2662716 |
| CPS134 | *S10_2662720 |
| CPS135 | *S10_2684257 |
| CPS136 | *S10_3128451 |
| CPS137 | *S10_4136973 |
| CPS138 | *S10_4188096 |
| CPS139 | *S10_4192320 |
| CPS140 | *S10_4313545 |
| CPS141 | *S10_4564892 |
| CPS142 | *S10_5495775 |
| CPS143 | *S10_5613719 |
| CPS144 | *S10_5640454 |
| CPS145 | *S10_5640462 |
| CPS146 | *S10_5640481 |
| CPS147 | *S10_5887374 |
| CPS148 | *S10_5944728 |
| CPS149 | *S10_5970326 |
| CPS150 | *S10_5970327 |
| CPS151 | *S10_6439925 |
| CPS152 | *S10_6581747 |
| CPS153 | *S10_6941921 |
| CPS154 | *S10_6945120 |
| CPS155 | *S10_6957504 |
| CPS156 | *S10_7157854 |
| CPS157 | *S10_7377052 |
| CPS158 | *S10_7377304 |
| CPS159 | *S10_7847276 |
| CPS160 | *S10_8458541 |
| CPS161 | *S10_8458860 |
| CPS162 | *S10_8769159 |
| CPS163 | *S10_8871646 |
| CPS164 | *S10_8884297 |
| CPS165 | *S10_8884300 |
| CPS166 | *S10_9195695 |
| CPS167 | *S10_9195708 |
| CPS168 | *S10_9195716 |
| CPS169 | *S10_9196232 |
| CPS170 | *S10_9395088 |
| CPS171 | *S10_9395089 |
| CPS172 | *S10_9395096 |
| CPS173 | *S10_9421579 |
| CPS174 | *S10_10458007 |
| CPS175 | *S10_10589487 |
| CPS176 | *S10_10589682 |
| CPS177 | *S10_10992063 |
| CPS178 | *S10_11670672 |
| CPS179 | *S10_12631888 |
| CPS180 | *S10_12631920 |
| CPS181 | *S10_13342536 |
| CPS182 | *S10_14817508 |
| CPS183 | *S10_14940305 |
| CPS184 | *S10_15092279 |
| CPS185 | *S10_17985641 |
| CPS186 | *S10_18297719 |
| CPS187 | *S10_18640838 |
| CPS188 | *S10_19288265 |
| CPS189 | *S10_25130029 |
| CPS190 | *S10_25343155 |
| CPS191 | *S10_26862218 |
| CPS192 | *S10_32567838 |
| CPS193 | *S10_34870500 |
| CPS194 | *S10_36899760 |
| CPS195 | *S10_39307682 |
| CPS196 | *S10_39704900 |
| CPS197 | *S10_40040280 |
| CPS198 | *S10_40040293 |
| CPS199 | *S10_40087803 |
| CPS200 | *S10_40091141 |
| CPS201 | *S10_42455170 |
| CPS202 | *S10_43265369 |
| CPS203 | *S10_45553756 |
| CPS204 | *S10_46065919 |
| CPS205 | *S10_46066519 |
| CPS206 | *S10_46207407 |
| CPS207 | *S10_46207409 |
| CPS208 | *S10_46576831 |
| CPS209 | *S10_47834765 |
| CPS210 | *S10_47939467 |
| CPS211 | *S10_48654661 |
| CPS212 | *S10_49098541 |
| CPS213 | *S10_49098545 |
| CPS214 | *S10_49098547 |
| CPS215 | *S10_49359825 |
| CPS216 | *S10_49569951 |
| CPS217 | *S10_49569954 |
| CPS218 | *S10_50429222 |
| CPS219 | *S10_50768462 |
| CPS220 | *S10_50809263 |
| CPS221 | *S10_51140875 |
| CPS222 | *S10_51141996 |
| CPS223 | *S10_52516301 |
| CPS224 | *S10_52797931 |
| CPS225 | *S10_53777814 |
| CPS226 | *S10_54101946 |
| CPS227 | *S10_55146978 |
| CPS228 | *S10_55376809 |
| CPS229 | *S10_55376810 |
| CPS230 | *S10_55599561 |
| CPS231 | *S10_55601753 |
| CPS232 | *S10_56234592 |
| CPS233 | *S10_56249757 |
| CPS234 | *S10_56249773 |
| CPS235 | *S10_56485894 |
| CPS236 | *S10_56736550 |
| CPS237 | *S10_57453669 |
| CPS238 | *S10_57592932 |
| CPS239 | *S10_58154909 |
| CPS240 | *S10_59192301 |
| CPS241 | *S10_59192307 |
| CPS242 | *S10_59202679 |
| CPS243 | *S10_59418920 |
| CPS244 | *S10_59419647 |
| CPS245 | *S10_59859596 |
| CPS246 | *S10_60283114 |
| CPS247 | *S10_60302682 |
| CPS248 | *S10_60302700 |
| CPS249 | *S10_60315730 |
| CPS250 | *S10_49079792 |
| CPS251 | *S10_234260 |
| CPS252 | *S10_386434 |
| CPS253 | *S10_424087 |
| CPS254 | *S10_445674 |
| CPS255 | *S10_550591 |
| CPS256 | *S10_550615 |
| CPS257 | *S10_615886 |
| CPS258 | *S10_679206 |
| CPS259 | *S10_794320 |
| CPS260 | *S10_871722 |
| CPS261 | *S10_960601 |
| CPS262 | *S10_964528 |
| CPS263 | *S10_964529 |
| CPS264 | *S10_964530 |
| CPS265 | *S10_973220 |
| CPS266 | *S10_1148647 |
| CPS267 | *S10_1188871 |
| CPS268 | *S10_1221866 |
| CPS269 | *S10_1230034 |
| CPS270 | *S10_1651372 |
| CPS271 | *S10_2411678 |
| CPS272 | *S10_2437219 |
| CPS273 | *S10_2469363 |
| CPS274 | *S10_2662713 |
| CPS275 | *S10_2857420 |
| CPS276 | *S10_3202202 |
| CPS277 | *S10_3205545 |
| CPS278 | *S10_3667127 |
| CPS279 | *S10_3922531 |
| CPS280 | *S10_4192325 |
| CPS281 | *S10_4192327 |
| CPS282 | *S10_4192328 |
| CPS283 | *S10_4192339 |
| CPS284 | *S10_5700924 |
| CPS285 | *S10_5941029 |
| CPS286 | *S10_5970347 |
| CPS287 | *S10_6330467 |
| CPS288 | *S10_6439898 |
| CPS289 | *S10_6439900 |
| CPS290 | *S10_6568111 |
| CPS291 | *S10_6904978 |
| CPS292 | *S10_6945116 |
| CPS293 | *S10_6957918 |
| CPS294 | *S10_6957921 |
| CPS295 | *S10_7377067 |
| CPS296 | *S10_7434843 |
| CPS297 | *S10_7435656 |
| CPS298 | *S10_7756878 |
| CPS299 | *S10_7830891 |
| CPS300 | *S10_7830903 |
| CPS301 | *S10_7847260 |
| CPS302 | *S10_7854001 |
| CPS303 | *S10_8387562 |
| CPS304 | *S10_8524848 |
| CPS305 | *S10_8526778 |
| CPS306 | *S10_8776124 |
| CPS307 | *S10_8804149 |
| CPS308 | *S10_8959113 |
| CPS309 | *S10_8959126 |
| CPS310 | *S10_9163475 |
| CPS311 | *S10_9181174 |
| CPS312 | *S10_9195715 |
| CPS313 | *S10_9561204 |
| CPS314 | *S10_10082556 |
| CPS315 | *S10_10232757 |
| CPS316 | *S10_10589499 |
| CPS317 | *S10_11670675 |
| CPS318 | *S10_11734173 |
| CPS319 | *S10_12631886 |
| CPS320 | *S10_13719403 |
| CPS321 | *S10_15092270 |
| CPS322 | *S10_15194993 |
| CPS323 | *S10_15196768 |
| CPS324 | *S10_18199230 |
| CPS325 | *S10_18201667 |
| CPS326 | *S10_18640797 |
| CPS327 | *S10_18645206 |
| CPS328 | *S10_21563058 |
| CPS329 | *S10_21763179 |
| CPS330 | *S10_22918086 |
| CPS331 | *S10_25729427 |
| CPS332 | *S10_26862215 |
| CPS333 | *S10_34428856 |
| CPS334 | *S10_36899779 |
| CPS335 | *S10_37775943 |
| CPS336 | *S10_37784421 |
| CPS337 | *S10_37868660 |
| CPS338 | *S10_38273943 |
| CPS339 | *S10_38790525 |
| CPS340 | *S10_41189638 |
| CPS341 | *S10_42135201 |
| CPS342 | *S10_43409284 |
| CPS343 | *S10_46207405 |
| CPS344 | *S10_46550769 |
| CPS345 | *S10_47834783 |
| CPS346 | *S10_47939474 |
| CPS347 | *S10_47980222 |
| CPS348 | *S10_47980348 |
| CPS349 | *S10_48055547 |
| CPS350 | *S10_48084387 |
| CPS351 | *S10_48084405 |
| CPS352 | *S10_48865446 |
| CPS353 | *S10_49098543 |
| CPS354 | *S10_49098544 |
| CPS355 | *S10_49098546 |
| CPS356 | *S10_49098553 |
| CPS357 | *S10_49382641 |
| CPS358 | *S10_49569964 |
| CPS359 | *S10_49639848 |
| CPS360 | *S10_49672531 |
| CPS361 | *S10_49894492 |
| CPS362 | *S10_49934365 |
| CPS363 | *S10_50040584 |
| CPS364 | *S10_50171706 |
| CPS365 | *S10_50661736 |
| CPS366 | *S10_50809289 |
| CPS367 | *S10_52582870 |
| CPS368 | *S10_52676228 |
| CPS369 | *S10_54032213 |
| CPS370 | *S10_55146973 |
| CPS371 | *S10_55146981 |
| CPS372 | *S10_55376811 |
| CPS373 | *S10_55755202 |
| CPS374 | *S10_55755222 |
| CPS375 | *S10_55755256 |
| CPS376 | *S10_56234635 |
| CPS377 | *S10_56626763 |
| CPS378 | *S10_56858864 |
| CPS379 | *S10_58629139 |
| CPS380 | *S10_59418882 |
| CPS381 | *S10_60264959 |
| CPS382 | *S10_60324251 |
| CPS383 | *S10_60324265 |
| CPS384 | *S10_60491965 |
| CPS385 | *S10_54585048 |
| CPS386 | *S10_439578 |
| CPS387 | *S10_584839 |
| CPS388 | *S10_630483 |
| CPS389 | *S10_1188857 |
| CPS390 | *S10_1188859 |
| CPS391 | *S10_1188862 |
| CPS392 | *S10_1188867 |
| CPS393 | *S10_1188873 |
| CPS394 | *S10_1233173 |
| CPS395 | *S10_1240783 |
| CPS396 | *S10_2358550 |
| CPS397 | *S10_2857380 |
| CPS398 | *S10_3226622 |
| CPS399 | *S10_4192318 |
| CPS400 | *S10_4195092 |
| CPS401 | *S10_4598620 |
| CPS402 | *S10_4717918 |
| CPS403 | *S10_5887375 |
| CPS404 | *S10_5944735 |
| CPS405 | *S10_5970323 |
| CPS406 | *S10_5970329 |
| CPS407 | *S10_5970349 |
| CPS408 | *S10_5970353 |
| CPS409 | *S10_5970354 |
| CPS410 | *S10_6206246 |
| CPS411 | *S10_6439901 |
| CPS412 | *S10_6439905 |
| CPS413 | *S10_6567742 |
| CPS414 | *S10_6945117 |
| CPS415 | *S10_7162917 |
| CPS416 | *S10_7377049 |
| CPS417 | *S10_8483044 |
| CPS418 | *S10_8661845 |
| CPS419 | *S10_8769119 |
| CPS420 | *S10_8769152 |
| CPS421 | *S10_8871647 |
| CPS422 | *S10_8883782 |
| CPS423 | *S10_8884301 |
| CPS424 | *S10_9339171 |
| CPS425 | *S10_9395054 |
| CPS426 | *S10_9583214 |
| CPS427 | *S10_9790180 |
| CPS428 | *S10_10083023 |
| CPS429 | *S10_11119240 |
| CPS430 | *S10_12627531 |
| CPS431 | *S10_12627556 |
| CPS432 | *S10_12631904 |
| CPS433 | *S10_14628755 |
| CPS434 | *S10_14940255 |
| CPS435 | *S10_15072145 |
| CPS436 | *S10_15196753 |
| CPS437 | *S10_18199239 |
| CPS438 | *S10_18645679 |
| CPS439 | *S10_20923883 |
| CPS440 | *S10_21786890 |
| CPS441 | *S10_22392694 |
| CPS442 | *S10_22416376 |
| CPS443 | *S10_23243883 |
| CPS444 | *S10_23380716 |
| CPS445 | *S10_25690617 |
| CPS446 | *S10_33802674 |
| CPS447 | *S10_33841120 |
| CPS448 | *S10_38663434 |
| CPS449 | *S10_40087907 |
| CPS450 | *S10_41811694 |
| CPS451 | *S10_44252822 |
| CPS452 | *S10_44414729 |
| CPS453 | *S10_44659872 |
| CPS454 | *S10_45865300 |
| CPS455 | *S10_45865309 |
| CPS456 | *S10_46207383 |
| CPS457 | *S10_46207404 |
| CPS458 | *S10_47763773 |
| CPS459 | *S10_47780008 |
| CPS460 | *S10_47850431 |
| CPS461 | *S10_47980201 |
| CPS462 | *S10_47980351 |
| CPS463 | *S10_49084022 |
| CPS464 | *S10_49084088 |
| CPS465 | *S10_49098569 |
| CPS466 | *S10_49359785 |
| CPS467 | *S10_49640633 |
| CPS468 | *S10_50381897 |
| CPS469 | *S10_50595482 |
| CPS470 | *S10_50809283 |
| CPS471 | *S10_50810199 |
| CPS472 | *S10_51771609 |
| CPS473 | *S10_52396079 |
| CPS474 | *S10_52797875 |
| CPS475 | *S10_53810291 |
| CPS476 | *S10_53832841 |
| CPS477 | *S10_54032091 |
| CPS478 | *S10_54032212 |
| CPS479 | *S10_54086531 |
| CPS480 | *S10_55068494 |
| CPS481 | *S10_55068497 |
| CPS482 | *S10_55110289 |
| CPS483 | *S10_55146984 |
| CPS484 | *S10_55146990 |
| CPS485 | *S10_55671611 |
| CPS486 | *S10_56485883 |
| CPS487 | *S10_57294656 |
| CPS488 | *S10_58023929 |
| CPS489 | *S10_58621095 |
| CPS490 | *S10_59082143 |
| CPS491 | *S10_60324270 |
| CPS492 | *S10_60324273 |
| CPS493 | *S10_60349808 |
| CPS494 | *S10_60413870 |
| CPS495 | *S10_60413895 |
| CPS496 | *S10_60507685 |
